# Prevalence, lived experiences and user profiles in e-cigarette use: A mixed methods study among French college students

**DOI:** 10.1371/journal.pone.0297156

**Published:** 2024-02-09

**Authors:** Shérazade Kinouani, Héléna Da Cruz, Emmanuel Langlois, Christophe Tzourio

**Affiliations:** 1 University of Bordeaux, Inserm, Bordeaux Population Health Research Center, Team HEALTHY, UMR 1219, Bordeaux, France; 2 Department of General Practice, University of Bordeaux, Bordeaux, France; 3 University of Bordeaux, CNRS, Emile Durkheim Center, UMR 5116, Bordeaux, France; University of Montana, UNITED STATES

## Abstract

**Background:**

Little is known about e-cigarette use in French students. Our aims were to estimate the prevalence of e-cigarette experimentation and current e-cigarette use; describe the reasons for using e-cigarettes; explore the vaping experience and identify the profiles of e-cigarette users.

**Methods:**

We used a sequential, explanatory mixed methods design in a sample of French college students. Quantitative data was collected online for a cross-sectional analysis among 1698 students. Two separate analysis based on the thematic analysis and the Grounded Theory were also performed in 20 semi-structured interviews, focusing former and current smokers also current vapers.

**Results:**

The prevalence of e-cigarette experimentation was 39.3% (95% CI: 35.2–44.0) and 5.1% (95% CI: 3.2–8.0) of students were current e-cigarette users. Experimentation was opportunistic while current usage was rational, requiring to acquire a personal electronic device, getting used to its technicality, appreciating its availability, discretion, and learning the practice. In this context, three distinct groups of e-cigarette users were identified, based on assumed identity, tobacco and e-cigarette use, the functions assigned to e-cigarettes, and intentions with regards to vaping in the future.

**Conclusion:**

Despite some limitations mainly related to the participants self-selection, this research showed that while many smokers and former smokers have tried e-cigarettes in this student population, few have continued to use them continuously. Moreover, these current e-cigarette users were a heterogeneous group. Longitudinal studies are needed in young adult smokers for a better understanding of how their tobacco and e-cigarette use affect each other and change over time.

## Introduction

E-cigarette use (or vaping) has increased worldwide, particularly among young adults [1,2]. Although toxicological studies suggest it is less harmful than smoking, the risks of long-term on human health are as yet unknown [3,4]. Several observational studies have suggested that vaping could be associated with a later cigarette smoking in never smokers [5,6] or a later risk of relapse in former smokers [7,8]. It is probable that the risk-benefit balance of chronic vaping will always be disadvantageous in never smokers who vape, as well as in current smokers who do not quit smoking completely, engaging in dual use of tobacco and vaping [9,10]. Despite these uncertainties, e-cigarettes are reported to be the most popular tool among young Europeans to quit smoking. Among current tobacco smokers who participated to the Eurobarometer Survey in 2020, those aged 15–24 were less likely to have attempted to stop smoking compared with those aged ≥ 25 years. While nearly three quarters of Europeans ≥15 years old who tried to quit smoking did so without any help, 29% used a cessation aid. The most frequently used aids were: first pharmacotherapy and second e-cigarettes. Those aged 15–24 were less likely to use pharmacotherapy and more likely to use e-cigarettes than those aged ≥55 years [11]. These results are roughly comparable to what was described by the same survey in 2017 [12].

According to the French national public health agency *Santé Publique France*: 37% of French people aged 18–75 tried e-cigarettes in 2020; 5.4% were currently using them, of which three quarters (4.3%) were using them daily [13]. E-cigarettes are not considered as medical devices in France but consumer products whose use, sale and advertising remain strongly regulated. Vaping products (e-liquids and electronic devices) on the French market comply with European regulations since May 2016 setting maximum nicotine content in e-liquids at 20 mg/ml [14]. Vaping is authorized everywhere, except in places frequented by minors, closed spaces or places where use is prohibited by the internal rules of the establishment. Selling to people under 18 is also prohibited, even online and advertising for vaping products has been banned. Compared to other countries, the French regulatory context can be seen as “moderate” regarding e-cigarettes. Unlike the United Kingdom, neither the legislative framework nor the health agencies promote e-cigarettes as a smoking cessation tool. However, they are not considered tobacco products, as in Mexico or Turkey. The French position on the regulation of e-cigarette use should be interpreted in the light of the evolution of tobacco use in recent years. In 2020, the prevalence of daily tobacco smoking was 25% among French adults [13]. Although this prevalence has decreased over the last 20 years, it remains among the highest in Europe [15].

Our study aimed to understand how e-cigarettes were used and perceived by French college students in their particular context. First, we wanted to estimate the levels of experimentation and current use of e-cigarettes in this population, which were not known. The prevalence were estimated in the whole sample and then according to smoking status. Second, we wanted to interpret these prevalence by taking into account initial intentions to vaping, the evolution of these intentions over time, and the experience of students who have maintained continuous use of e-cigarettes over a few months. Our general aim was to describe e-cigarette use in a French student population. The specific objectives were: i) estimating prevalence of e-cigarette experimentation and current e-cigarette use; ii) describing reasons for using e-cigarettes; iii) exploring vaping experiences and identifying e-cigarette user profiles in current and former smokers.

## Materials and methods

We conducted a sequential explanatory mixed methods study: QUANT → qual [16]. This article follows STROBE recommendations for reporting observational studies [17] and COREQ for reporting qualitative research [18].

### Data sources and participants in the quantitative phase

We carried out an online quantitative study among students who had already participated in the i-Share research project (Internet-based Students Health Research Enterprise), an ongoing e-cohort of French speaking students (French speaking students with French nationality like French speaking international students): www.i-share.fr. All students included in the i-Share project between February 2013 and January 2016 were contacted by e-mail to participate in our ancillary study on vaping. To be included in our analysis, the volunteers had to be at least 18, know how to read and understand French, and declare themselves enrolled in a higher education institution from one French university (University of Bordeaux). Data were collected between February and April 2016.

### Data collection and participants in the qualitative phase

Students at University of Bordeaux were invited by an advertisement via university social networks or e-mails. Those who answered the quantitative phase were also contacted by e-mail if they had reported trying e-cigarettes. Finally, we asked interviewed students if they knew other users who might be interested in participating (snowball strategy). To be included, volunteers had to be current e-cigarette users or have used them frequently for at least two continuous months, be studying at Bordeaux University, and be current or former smokers. We obtained a purposive sample based on three criteria: gender, field of study and smoking status. Smoking status included recent former smokers (had quit less than one year before), older former smokers (had quit for at least one year or more), occasional smokers (smoked less than one cigarette per day) and daily smokers.

Semi-structured individual interviews took place between April 2016 and June 2017. Two trained medical students involved in the research team led all interviews because of their proximity in age to the respondents. Interviews were audio-recorded and then transcribed. The initial guide was drafted by the research team based on the literature and the first results of the quantitative phase. It was then modified as interviews were conducted, and new hypotheses emerged. The final version is available as supporting information (S1 and S2 Files). At the end of the eighteenth interview, we seemed to have reached data saturation. Two other interviews were added, without any new themes or categories emerging.

### Analyses

#### Quantitative component

The main outcomes were experimentation with e-cigarettes (defined as trying at least once in a lifetime) and current use of e-cigarettes (defined as daily or occasional use of e-cigarettes). We analyzed several sociodemographic, economic, academic, and medical characteristics from the i-Share baseline questionnaire when they were available (S3 and S4 Files). We also analyzed ancillary study data on smoking status and e-cigarette use (age of first try, reasons for trying e-cigarettes, current use). In current e-cigarette users, we explored reasons for using e-cigarettes, use frequency, nicotine use in e-liquids, vaping places and times. We described continuous variables using median and interquartile range (IQR) and categorical variables using numbers and proportions. Bivariate comparison was performed by chi-square test. First, we described the sociodemographic, economic, academic, and medical characteristics in the whole sample. Secondly, we estimated the prevalence of e-cigarette experimentation and current e-cigarette use with 95% confidence interval (95% CI). We calculated them before and after weighting by calibration on the known margins of the student population at the University for the 2015–2016 academic year [19]. This calibration was carried out with a program developed by the French National Institute for Statistics and Economic Studies designed to take into account the non-response bias: the macroSAS CALMAR® [20]. Calibration variables were gender, age, and study fields. Thirdly, we described e-cigarette use in those students who have experimented with it and associations between e-cigarette use and smoking status were analyzed in this subsample. All p-values were two-tailed and we considered p < 0.05 to be statistically significant. All statistical analyses were performed with R® (version 4.0.2).

#### Qualitative component

A pseudonym was assigned to each participant during interview transcription and used when attributing quotes. We carried out two separate but concomitant analyses of the same data. First, we performed a thematic analysis on reasons for using e-cigarettes, starting at the end of data collection of the quantitative phase [21]. An analysis based on Grounded Theory was also performed to understand the lived experiences and user profiles [22,23]. Thematic analysis is an analytical method allowing to both test the motives for using e-cigarettes identified in the quantitative phase and to highlight convergences and divergences between the motives for experimenting and those for continuing to use electronic cigarettes. On the other hand, the analysis inspired by Grounded Theory seemed more appropriate to bring out the conceptualizing categories describing the lived experiences of vapers or their user’s profile. Analyses were carried out by five trained researchers, either manually or using Nvivo 10 ®. Each interview was coded individually by at least two of the five researchers with iterative pooling times, until data saturation. The themes or categories obtained as the analyses progressed and final theorizing about user profiles were discussed by all co-authors. A synthesis of the study, its results and their interpretation were e-mailed to all interviewed students in April 2018 for comments.

#### Integrative mixed methods analysis

We adopted a building approach to the results [16], beginning by collecting and analyzing quantitative data and then using these findings to guide data collection and analysis in the qualitative phase. The quantitative phase described the prevalence and reasons for using e-cigarettes in our student population. Reasons identified in the quantitative component were investigated during the thematic analysis of data from the qualitative component. An analysis based on Grounded Theory focused in parallel on lived experiences of vaping among university students who regularly used e-cigarettes (for at least two continuous months), whether they were dual users, former smokers and relapsing smokers.

### Ethics

This research was conducted in accordance with the Declaration of Helsinki. All subjects in the quantitative phase gave their informed consent before participating in the i-Share project and ancillary studies. The i-Share project protocol was approved by the *Commission Informatique et Libertés*, the national authority that ensures that data collection in research does not violate freedoms, rights, and human privacy (number: DR-2013-019). Students participating in the online quantitative study on e-cigarettes received points that can be exchanged for cinema tickets or fruit and vegetable hampers. The qualitative phase received ethical approval from the ethics committee of Bordeaux University Hospital, France (number: GP-CE 2018/17). All subjects gave their oral consent at the beginning of the audio-recording.

## Results

### Quantitative phase

**Fig 1:**
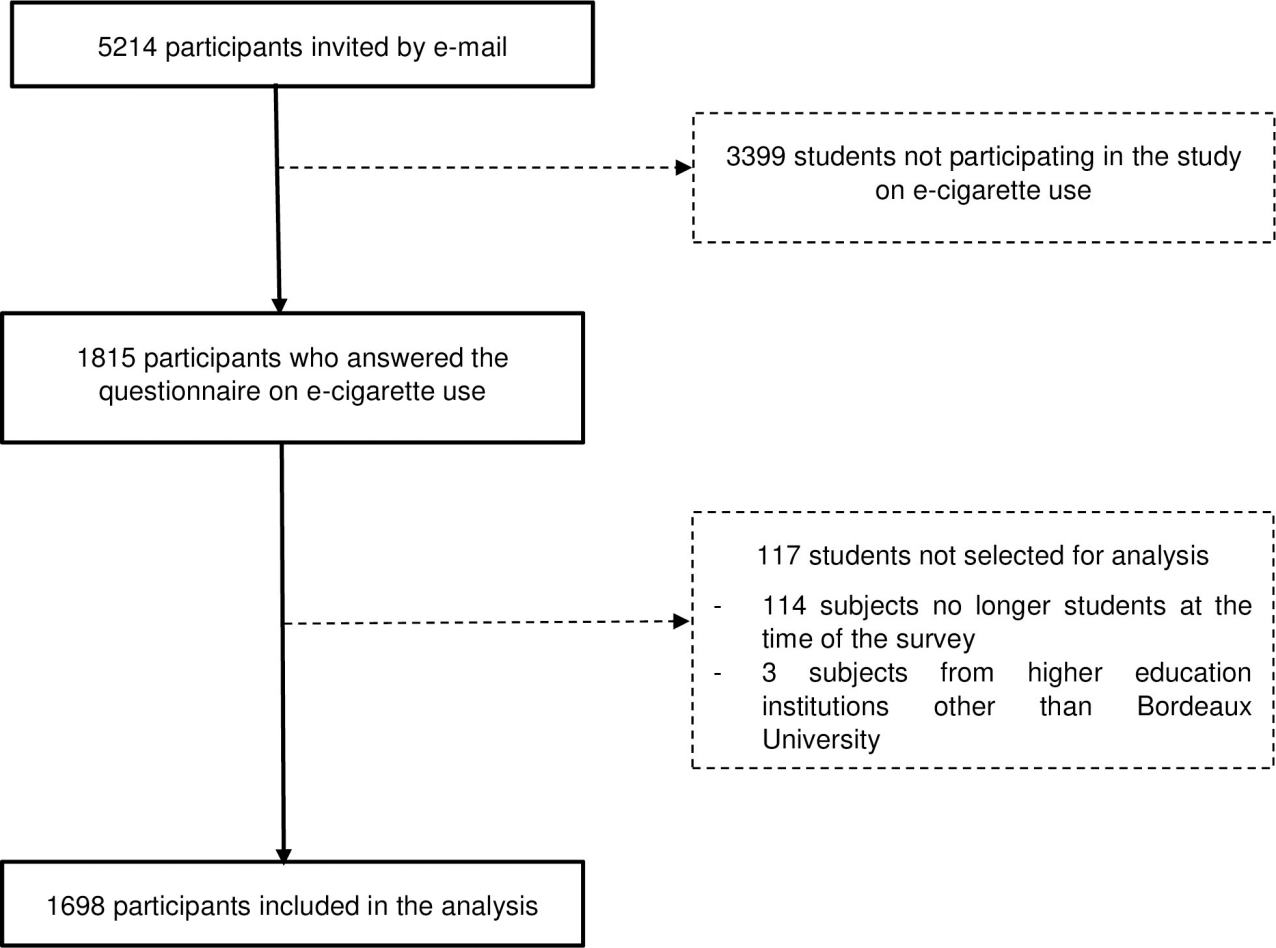
i-Share project students participating in the ancillary quantitative study on e-cigarette use at the University of Bordeaux (France) in 2016.

The quantitative component comprised 5214 students invited to participate in the ancillary study on e-cigarette use; 1815 subjects answered (response rate: 34.8%) and 1698 students were finally kept for the analyses (Fig 1).

More than 3/4 of these students were female. More than two out of five students were freshmen and 90% were 24 or under (median age: 21; IQR: 19.0–23.0). Just under half (46%) were in the healthcare field and the parents of 53% of them had had higher education. More than 80% of them perceived their health as good or very good. Table 1 summarizes their characteristics.

**Table 1:** Characteristics of college students who participated in the quantitative study on e-cigarette use at the University of Bordeaux in 2016, N = 1698.

Characteristics	n	%
Duration between the inclusion in i-Share project and those in the ancillary study, in weeks (median, IQR*)	60	25–102
Gender ^α^		
• Men	375	22.1
• Women	1323	77.9
Age, in years ^α^		
• 18–20	775	45.7
• 21–24	768	45.2
• 25 and over	155	9.1
Academic study fields ^α^		
• Healthcare	774	45.6
• Literature arts, Humanities and social sciences	391	23.0
• Sciences	214	12.6
• Economics, management and law	133	7.8
• Other	186	11.0
Academic year of study ^α^		
• 1^st^ year	754	44.4
• 2^nd^ year	340	20.0
• 3^rd^ year	234	13.8
• Beyond 3^rd^ year	342	20.2
• Other	28	1.6
Students’ living conditions ^α^		
• In apartment: couple, or colocation	462	27.2
• In apartment, alone	576	33.9
• Parents’ home	409	24.1
• University residence	183	10.8
• Other	68	4.0
Student economic resources, in multiple choice ^α^		
• Family	1386	81.6
• Scholarship	753	44.3
• Paid job (including summer job, paid internship)	677	39.9
• Other	103	6.1
Parents’ educational level ^α^		
• Higher education, university	897	52.8
• High school or vocational study	762	44.9
• Primary education	10	0.6
• I don’t know	29	1.7
Self-rated of current health^α^		
• Very good to good	1380	81.3
• Fair	275	16.2
• Poor to very poor	43	2.5
Self-rated of sleep quality over the past 3 months^α^		
• Good	953	56.1
• Neither good nor poor	420	24.7
• Poor	325	19.2
Diagnosis previously made by a physician ^α^		
• Headaches	355	20.9
• Asthma	334	19.7
• Anxiety and phobic disorders	236	13.9
• Depression	174	10.2
Smoking status ^β^		
• Current smokers	833	49.3
• Former smokers	164	9.7
• Never smokers	694	41.0
Current alcohol use frequency ^α^		
• Never	100	5.8
• ≤ Monthly	429	25.3
• > Monthly but not weekly	842	49.2
• > Weekly but not daily	319	18.8
• Daily	8	0.5
Cannabis use at least once in lifetime ^α^	884	52.1

^*^ *IQR*: *Interquartile range;*

^*α*^ *Data collected at baseline in the i-Share project between February 2013 and January 2016;*

^*β*^*Data collected in the ancillary quantitative study on e-cigarette use*.

The weighted prevalence of e-cigarette experimentation in the student population was 39.3%, 95% CI: 35.2–44.0 (Table 2). The median age of first trying e-cigarettes was 20, IQR: 18.0–21.0. There was more experimentation in former and current smokers than in never smokers (Table 3). Curiosity, the opportunity to try and the diversity of flavors were the main reasons for experimenting with them (S1 Table).

**Table 2:** Prevalence of e-cigarette use among college students at the University of Bordeaux in 2016, N = 1694*.

E-cigarette use	n	Crude %	95% CI^β^	Weighted % ^α^	95% CI^β^
No experiment ^σ^	991	58.5	56.1–60.9	55.6	51.3–60.0
Experiment ^γ^	645	38.1	35.8–40.4	39.3	35.2–44.0
Current use ^ω^	58	3.4	2.6–4.4	5.1	3.2–8.0
• Occasional use	28	1.6	1.1–2.4	2.4	1.2–5.0
• Daily use	30	1.6	1.2–2.5	2.7	1.5–5.0

*1698 subjects included in analysis but only data concerning 1694 subjects were available on e-cigarette use and calibration variables.

^*α*^ *Weighting by calibration on margins*, *using the MacroSAS Calmar® program (raking ratio method)*. *The calibration variables were*: *Gender*, *age and the study fields;*

^*β*^ *95% confidence interval;*

^*σ*^ *Never tried to use e-cigarettes;*

^*γ*^ *Having tried at least once to use e-cigarettes;*

^*ω*^ *Occasional (<1 time per day) or daily (≥ 1 time per day) use of e-cigarettes*.

**Table 3:** Prevalence of e-cigarette use according to smoking status among college students at the University of Bordeaux in 2016, N = 1691*.

E-cigarette use	n	Crude %	p-value^β^	Weighted % ^α^	p-value^β^
No experiment ^σ^	989	58.5	<0.0001	55.6	<0.0001
• Never smokers	593	85.4		82.4	
• Former smokers	47	28.7		29.9	
• Current smokers	349	41.9		40.7	
Experiment ^γ^	644	38.1	<0.0001	39.3	<0.0001
• Never smokers	99	14.3		17.4	
• Former smokers	97	59.1		55.5	
• Current smokers	448	53.8		52.6	
Current use ^ω^	58	3.4	<0.0001	5.1	<0.0001
• Never smokers	2	0.3		0.2	
• Former smokers	20	12.2		14.6	
• Current smokers	36	4.3		6.7	

*1698 subjects included in analysis but only data concerning 1691 subjects were available on e-cigarette use, smoking status and calibration variables.

^*α*^ *Weighting by calibration on margins*, *using the MacroSAS Calmar® program (raking ratio method)*. *The calibration variables were*: *Gender*, *age and the study fields;*

^*β*^ *Chi square test;*

^*σ*^ *Never tried to use e-cigarettes;*

^*γ*^ *Having tried at least once to use e-cigarettes;*

^*ω*^ *Occasional (<1 time per day) or daily (≥ 1 time per day) use of e-cigarettes*.

The weighted prevalence of current use in the student population was 5.1%, (95% CI: 3.2–8.0) (Table 2). Current use was most frequent in former smokers, followed by current smokers, but was rare in never smokers (Table 3). The majority of the 58 current users in the quantitative study vaped e-liquids containing nicotine, but five did not know if their e-liquids contained nicotine (S1 Table). There rarely seemed to be a single reason to continue using e-cigarettes. The four predominant reasons for current use reported by students were either related to their ease of use in time and space or related to the management of addiction symptoms (S1 Table).

### Qualitative phase

#### Sample

As shown in Table 4, 20 students were interviewed in the qualitative component, 11 men and 9 women (median age: 26; IQR: 23.7–28.0). The interviews lasted on average 55.25 minutes. Fourteen students were former smokers and six were dual users, combining vaping with smoking. One of these dual users was a relapsed tobacco user.

**Table 4:** Characteristics of college students participating in the qualitative study on e-cigarette use at the University of Bordeaux in 2016–2017, N = 20.

Characteristics	n (%)
Age, in years	
• 19–25	10 (50)
• 26–29	10 (50)
Gender	
• Men	11 (55)
• Women	9 (45)
Participants in i-Share project ^α^	7 (35)
Academic study fields	
• Healthcare	11
	*Medicine*: *9*
	*Public health*: *1*
	*Pharmacy*: *1*
• Humanities and social sciences	2
• Sciences	3
	*Life sciences*: *2*
	*Information technology*: *1*
• Economics, management and law	2
• Literature and arts	2
Smoking status	
• Former smokers, > 1 year	8 (40)
• Former smokers, ≤ 1 year	6 (30)
• Daily smokers (≥ 1 cigarette per day)	3 (15)
• Occasional smokers (< 1 cigarette per day)	3 (15)
E-cigarette use	
• Former users	4 (20)
• Daily users (≥ 1 inhalation per day)	14 (70)
• Occasional users (<1 inhalation per day)	2 (10)

^*α*^ *Seven college students participating in i-Share project answered the ancillary quantitative study about e-cigarette use and also accepted to be interviewed in the qualitative phase*.

### Thematic analysis in the qualitative phase

**Fig 2:**
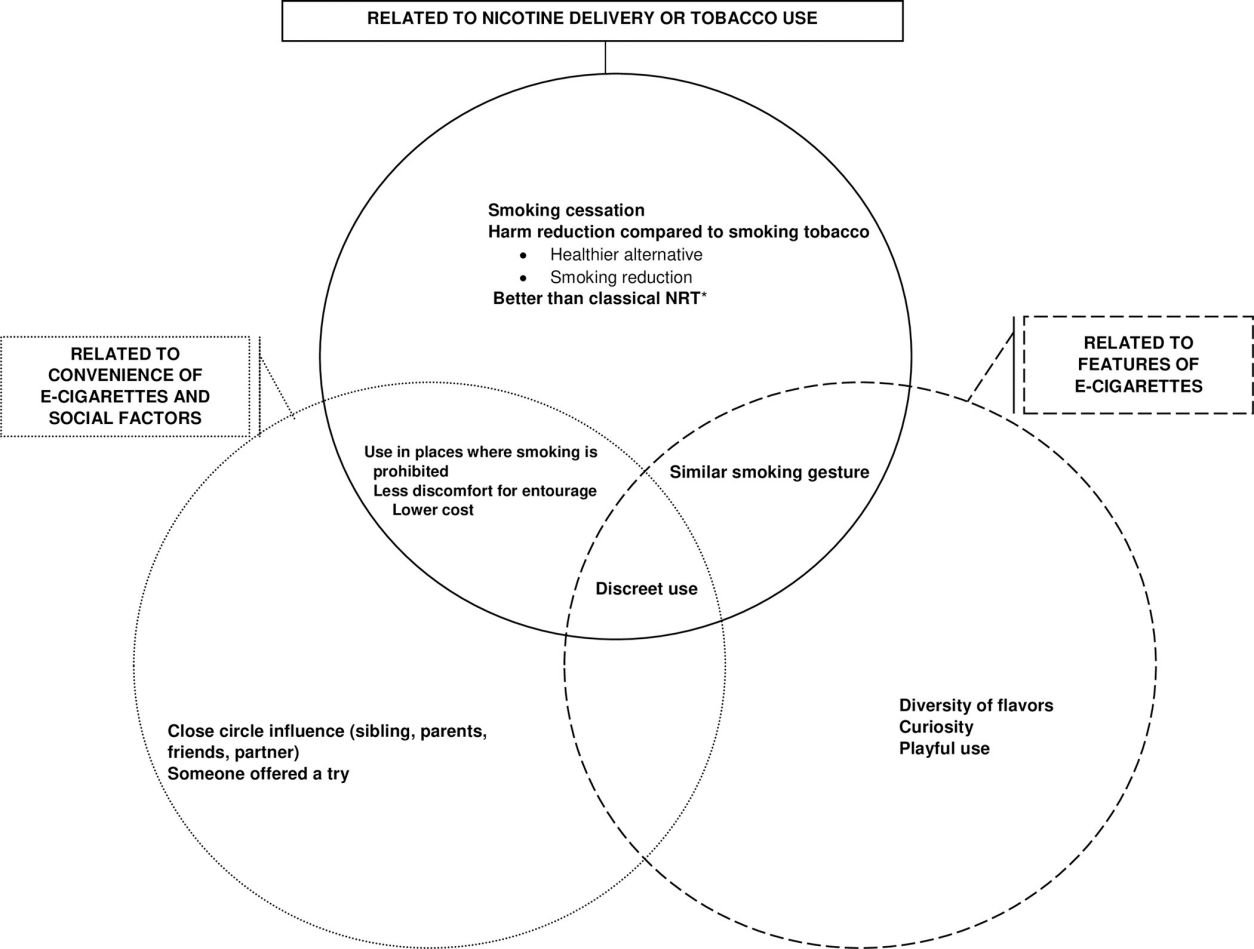
Reasons for trying e-cigarette use among current and former tobacco smokers of the University of Bordeaux, N = 20. *NRT: Nicotine replacement therapy.

Overall, three main themes were identified as reasons for experimenting with e-cigarettes (Fig 2): reasons related to their features; reasons related to nicotine delivery or tobacco use; and reasons related to the convenience of vaping and social interactions. The opportunity to try and reasons related to the features of e-cigarettes had a major influence on the first try, even in those who finally quit smoking (S1 Table). Moreover, vaping had seemed less expensive than smoking or easier and more pleasant than pharmacotherapy (S1 Table). Friends, family or a partner who already used e-cigarettes greatly contributed to their initiation.

**Fig 3:**
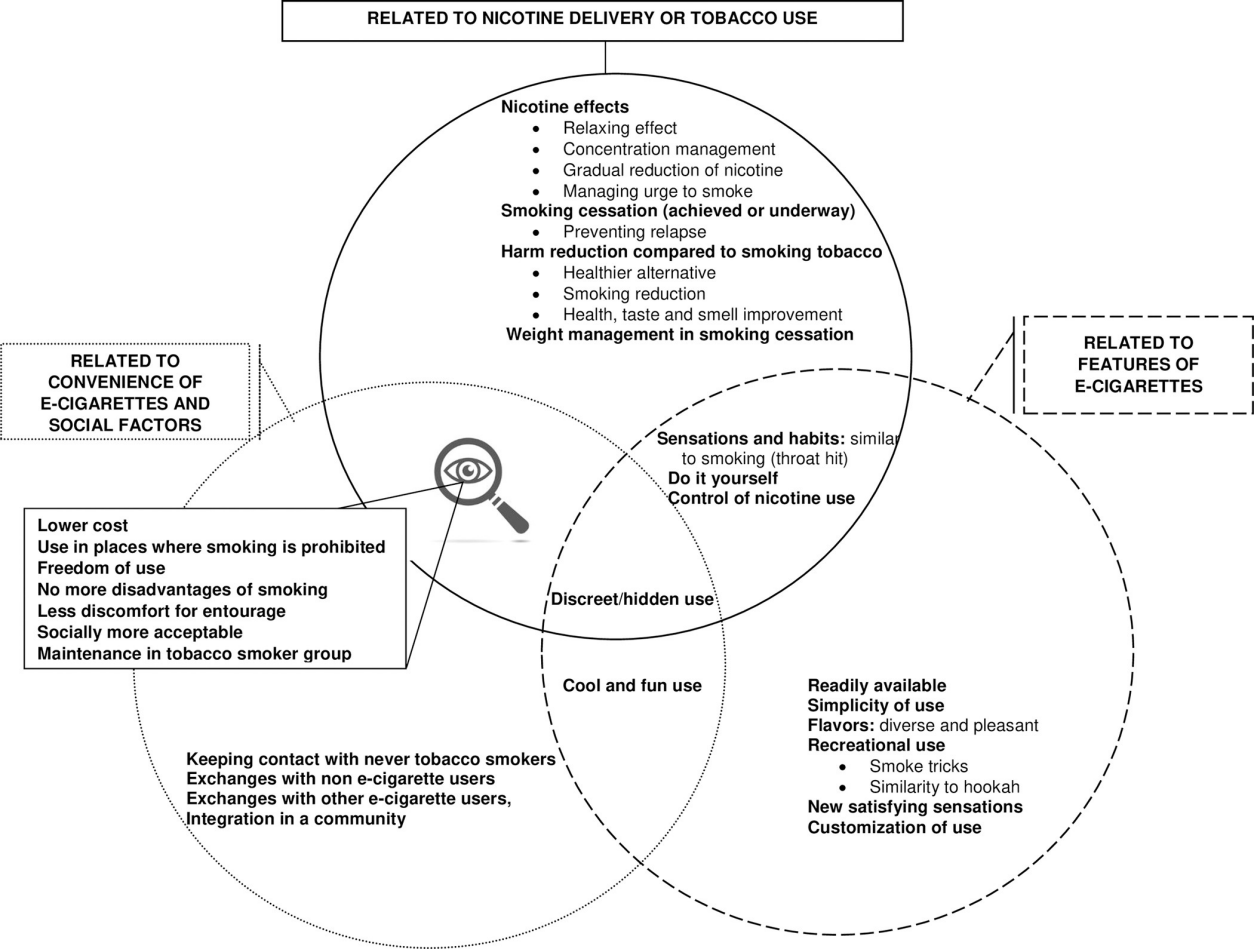
Reasons for pursuing e-cigarette use among current and former tobacco smokers of the University of Bordeaux, N = 20.

The same three main themes were found as reasons for pursuing e-cigarette use (Fig 3). However, their description was richer than for the experiment: the reported reasons were here multiple for each student. Whether dual users or former smokers, e-cigarette current use was explained mainly by comparing to tobacco (Fig 3, S1 Table). It was a way for some students to maintain their smoking habits, even when no longer smoking tobacco. Some said it helped break the day up. Others said it allowed them to continue enjoying the same gestures and sensations. It also produces the psychotropic effects of nicotine and helps users focus while working, but also relax. Many downsides of smoking were circumvented by e-cigarette use. Some saw e-cigarettes as less troublesome for those around them, owing to the lack of smell, or as a way around the ban on smoking in public places. Because they provide nicotine, e-cigarettes were also seen as a good substitute allowing a gradual reduction in tobacco or nicotine use, sometimes until cessation. Other reasons were unrelated to tobacco use, such as the possibility to customize use or the fact that vaping becomes a pleasure or leisure behavior (Fig 3).

### Grounded theory method applied in the qualitative phase: Vaping but not necessarily being a vaper

*Becoming a persevering e-cigarette user*. Three categories emerged: investment in a personal electronic device; seeking information; electronic device properties.

Students sometimes used a friend’s or relative’s device during experimentation but had to have their own device for regular use. They made their first purchase in stores or on Internet; their first personal e-cigarette was rarely a gift. Whatever its origin, becoming current use meant having its own device. Over time, some students changed devices and opted for technically more efficient models, offering better rendering of flavors, aerosol density, throat hit or battery life.

Current e-cigarette users often showed little interest in knowing more about vaping. When they did inquire, they mainly wanted information about the health effects, variety of flavors and ways to improve device performances or reduce the cost of use. The lack of interest in staying informed was claimed by some students as a way to keep a distance from their own usage and other users (the vaping community).

Three properties mainly characterized their electronic devices: discretion, availability, and above all, technicality. Discretion is ensured by less smoke production, no smell on clothing, or the small size of devices. It is easy to go to a store for devices, spare parts or e-liquid refills, or to buy them online.

"*I find that either ordering on Internet or going to a real store where you can try several tastes and talk to the seller*… *well*, *it’s more in the spirit of electronic cigarettes than going to buy an electronic cigarette and e-liquid from the tobacconist* " (Tao: man, former smoker and e-cigarette user for 4 months).

The technicality of devices was both a strength and weakness. Users needed to learn how to inhale the aerosol correctly for the expected effects, as with tobacco cigarettes, and also how to set and maintain their electronic device. This generated recurring malfunctions, repeated maintenance, and a personal effort to acquire technical skills, which discouraged some. Levels of personal interest in knowing more about vaping and electronic devices resulted in distinct attitudes. Either partial or total smoking substitution by vaping, conserving smoking habits (frequency, times and places) as much as possible; or adopting vaping as a new behavior, different from smoking and perceived as enjoyable.

*Self-image and social interactions of current users*. Vaping allowed some former smokers to manage their tobacco addiction. They had succeeded in replacing cigarettes with e-cigarettes and felt in control again. Other former smokers saw a lack of freedom, however, as e-cigarettes fed their addiction.

"*I think in a way yes*, *they (e-cigarettes) allowed me this first stop*, *and that made me say*: *well you can stop that (tobacco)"* (Anna: woman, former smoker who also quit e-cigarettes).

"*Well*, *you don’t stress out about your pack of cigarettes anymore but you stress out*… *if your thing (her e-cigarette) is loaded*. *Well*, *it’s*… *it’s the same as it is*… *we’re just so addicted to something all the time*. *And then it remains the same substance*: *nicotine* " (Bea: woman, former smoker and e-cigarette user for 2 years).

Users felt that vaping drew attention to themselves, and this was variably received. While some saw vaping as a way of giving a trendy image of themselves, others thought it made them look ridiculous, weak (vaping instead of smoking a real cigarette) or naive (vaping a dangerous product, regardless of health risk).

Non-users often showed surprise and curiosity during social interactions. They were tolerant and even wanted to know more about e-cigarette use: vaping was perceived overall as less harmful than smoking. Vaping favored social interactions between e-cigarette users. They shared knowledge, helped each other solve technical problems, talked about experiences of quitting smoking, etc. Some comments suggested membership of a subculture, notably via use of specific technical vocabulary to describe their device and practice, in particular among former smokers with a very personalized use of e-cigarettes. They were also those who described themselves as “vapers”.

**Fig 4:**
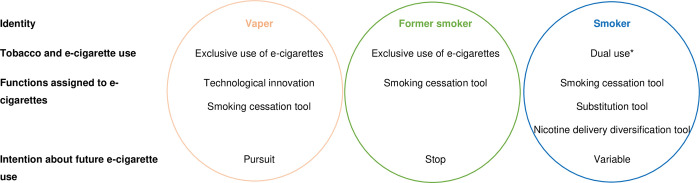
Relationship between user identity, the function assigned to e-cigarettes and the change in tobacco and e-cigarette use, among current and former smokers of the University of Bordeaux N = 20. * Concomitant use of tobacco and e-cigarettes.

*Current user profiles*. Three groups of students were distinguished, based on the four categories emerging from the analysis (Fig 4): function assigned to e-cigarettes; concomitant use of tobacco and e-cigarettes; intention about future e-cigarette use; identity.

Firstly, some users had succeeded in stopping smoking through e-cigarettes and saw them primarily as a technological innovation. Accumulating vaping knowledge and technical skills contributed to constructing their vaper identity. Vaping was a whole new experience, both personally and socially. They knowingly continued to use e-cigarettes after quitting smoking. Secondly, other former smokers rejected the vaper label. E-cigarettes were only a tool to stop smoking. They planned to quit smoking and achieved it through e-cigarettes or nicotine replacement therapies (if vaping was unsuccessful). These former smokers did not want vaping to participate in the construction of their identity, seeing vaping as a temporary stage. Many stopped using e-cigarettes after quitting smoking. Others continued to use e-cigarettes mainly for fear of relapse. Unlike the first two groups, common characteristics of the third group were their smoker identity and dual use. Based on the function assigned to e-cigarettes, three kinds of dual users were observed. Some perceived e-cigarettes as a tool to stop smoking and hoped that dual use was a step towards smoking cessation. Other dual users replaced tobacco by vaping except in circumstances where they felt that smoking could not be replaced, such as stressful events or evenings. The last dual users were not at all quitting or switching. E-cigarettes were an additional way of diversifying their nicotine use, particularly because of its practicality, the possibility of testing flavors, and so on.

### Integrative phase: From opportunistic experimentation to rational current use

According to the quantitative phase, experimentation with e-cigarettes was common among student former and current smokers. Although lesser, it also existed among never smokers. Quantitative and qualitative phases both suggested that experimentation with e-cigarettes was opportunistic. It was mainly favored by the student curiosity, a close circle who already used e-cigarettes, the diversity of flavors, or the playful aspect of practice. Only one in 20 students reported current e-cigarette use in the quantitative phase. According to the qualitative phase, this current use required: acquiring one’s own electronic device, getting used to its technicality, appreciating its availability, discretion, and learning the practice. Such personal investment explained why current use was marginal among student never smokers in our quantitative phase. To persevere in vaping, the experimenter indeed had to find enough advantages in use of e-cigarettes. After comparing vaping to smoking, former and current smokers all found many reasons to continue using them. Finally, vaping did not necessarily mean considering yourself to be a vaper. Students chose to pursue long-term e-cigarette use based on their assumed identity of smoker, former smoker, or vaper.

## Discussion

The quantitative phase showed that two in five students have tried e-cigarettes, but occasional or daily use was reported by only 5% of them. Reasons for using e-cigarettes changed from experimentation to current use. While the experimentation was common and opportunistic, the current use was less frequent but rational. By focusing on e-cigarette users for at least two continuous months, the qualitative phase also showed that e-cigarettes were used by former smokers as a means either to switch to a new, lasting and pleasant behavior, or as a transitory step towards stopping smoking. Dual users, on the other hand, formed a heterogeneous group. They partly replaced tobacco use by vaping but with various perspectives, not necessarily to quit smoking.

Our prevalence estimates of e-cigarette use were close to those described in adult populations in other high-income countries with moderately restrictive e-cigarette policies [24]. A recent study suggests that binding regulations on e-cigarettes could influence use in the adult population [25]. However, it has not been established whether they also impact current use of e-cigarettes among young adults [26]. Interpretation of e-cigarette use levels should also take tobacco control policies into account. According to the 2014 Eurobarometer survey, e-cigarette experimentation and current use among subjects aged 15 or over were higher in European countries that have increased tobacco taxes or promoted aid for smoking cessation [27]. But the effectiveness of tobacco control policies was not similar in all high-income countries. While they have succeeded in reducing the smoking rates in Canada or Australia, it remains at a high level in other countries such as France or Romania. For policymakers, e-cigarettes could be seen as detrimental to efforts that have helped reduce smoking rates, except in countries where those remain high: they may be more tolerated there as harm reduction tools [28].

Our results suggested that becoming a current user was a choice supported by many reasons identified by students. Similar results have been described in a qualitative study led among Hawaiian young adults who were daily e-cigarette users. They reported the same variety of reasons for regular use of e-cigarettes: smoking cessation/reduction, health improvement, sensory satisfaction, self-regulation induced by nicotine psychotropic effects, convenience of indoor smoking, cleaner alternative to cigarette smoking, discreet use, recreational use, social enhancement, etc. [29]. The role played by nicotine addiction in pursuit of vaping was not obscured by our results. They only underlined the multifactorial nature of the installation in this practice in young adult population, like other studies. Smoking, e-cigarette use and nicotine addiction were measured for four years in a longitudinal study among US adults aged 19–23 [30]. Among never smokers at baseline, e-cigarette use was not significantly associated with subsequent tobacco use, either directly or mediated by nicotine addiction. In contrast, tobacco use at baseline was associated with subsequent e-cigarette use in smokers, both directly and through nicotine addiction. The transition from smoking to e-cigarette use was therefore only partially mediated by nicotine addiction.

Our results showed three e-cigarette user profiles among students. Identical profiles were described in another study conducted among UK vapers aged 19 to 69 [31]. A similar regulatory framework in the United Kingdom and France concerning e-cigarettes (European Tobacco Products Directive) partly explained the convergence of results. Although several studies described profiles of e-cigarette users in various national contexts [31–36], few focused specifically on young adults [35,36]. One was a qualitative study of 20 Americans aged 21 to 27 in Massachusetts, a smoke-free state with a lot of restrictions on vaping [36]. Authors identified four e-cigarette user profiles according to personal and social purposes. This study also included vapers who had never smoked before, unlike ours.

Our analyses had some limitations. Tobacco and e-cigarette use were self-reported in the quantitative phase, with a risk of underestimating prevalence due to memorization bias or social desirability. The low response rate, the predominance of women or freshmen among participants suggested selection bias. The predominance in the sample of students whose parents had a high level of education and were the main source of income also suggested that most of participants had a favorable socio-economic level. It was not possible to weight the prevalence estimators on variables allowing to appreciate the socio-economic level of the students because this information was not available about the target population. Moreover, we did not take into account the regulatory framework on the use of tobacco or e-cigarettes in the country of origin of the international students included. In the qualitative phase, more than half of participants were healthcare students. Being future health professionals might have influenced their discourse in favor of smoking cessation benefits. Moreover, no information to assess the socio-economic level of students was collected in the qualitative phase. The profiles of e-cigarette users in our analysis might appear frozen, but some prospective observational quantitative studies suggest that vaping is a more dynamic process in smokers, even in young adults [37–39]. Where follow-up was long enough (≥ 12 months), multiple trajectories were observed with transitions from smoker to former smoker, from smoker to vaper, and so on. Finally, our studies were conducted in just one French university, limiting extrapolation to all French students or to young adults from countries with different e-cigarette regulations. Despite these weaknesses, our analysis seems to be the first to explore the relationship between e-cigarette and tobacco use in depth in a French student population, with a moderately restrictive regulation regarding e-cigarettes. With the quantitative study, we were able to estimate the weighted prevalence of experimentation and current use. The calibration method was used to reduce the effect of potential self-selection bias related to the voluntary participation of students which lead to an over-representation of women and freshmen in the quantitative phase. The qualitative study allowed us to propose profiles focusing on the lived experience of e-cigarette use.

## Conclusions

E-cigarette experimentation was frequent in this French student population, especially among smokers and former smokers. Current use was only reported by 5% of students. It was more reported by former and current smokers. The mixed approach provided a better understanding of the gap between this high level of experimentation and the relatively low level of current use. Moving from opportunistic experimentation to current use of e-cigarettes required having identified several arguments supporting this decision. Three distinct groups of users were identified: smoker (or dual user), former smoker and vaper. We also found that the current e-cigarette use was rare among never smoker students. Their reasons for continuing to use cigarettes and their identity characteristics were not explored in our qualitative study. They could deserve to be specifically studied in the “moderate” French regulatory context. Longitudinal quantitative or qualitative studies over more than one year in young adult smokers also appear necessary to understand the dynamics of their tobacco and e-cigarette use, in particular changes in identity.

## Supporting information

S1 Table Vaping among college students who had tried e-cigarettes in a quantitative phase^α^ (N = 704) and qualitative phase^β^ (N = 20) from a mixed methods study carried out at the University of Bordeaux (France), 2016–2017. *IQR: Interquartile range; ^α^Data collected in the online quantitative study on e-cigarette use; ^β^Data collected in qualitative research among former and current smokers who had used e-cigarettes for at least two continuous months.
(PDF)

S1 File Guide final en français. (PDF)

S2 File Final guide in English. (PDF)

S3 File Questionnaire d’inclusion dans le volet quantitatif en français. (PDF)

S4 File Baseline questionnaire in the quantitative phase in English. (PDF)

## References

[1] Laverty AA, Filippidis FT, Vardavas CI. Patterns, trends and determinants of e-cigarette use in 28 European Union Member States 2014–2017. Prev Med. 2018;116:13–8. doi: 10.1016/j.ypmed.2018.08.028 30144487

[2] Spears CA, Jones DM, Weaver SR, Huang J, Yang B, Pechacek TF, et al. Sociodemographic correlates of Electronic Nicotine Delivery Systems (ENDS) use in the United States, 2016–2017. Am J Public Health. 2019;109(9):1224–32. doi: 10.2105/AJPH.2019.305158 31318599 PMC6687271

[3] Helen GS, Eaton DL. Public Health Consequences of e-Cigarette Use. JAMA Intern Med. 2018;178(7):984–6. doi: 10.1001/jamainternmed.2018.1600 29801158 PMC6260959

[4] Hariri LP, Flashner BM, Kanarek DJ, O ‘Donnell Walter J., Soskis A, Ziehr DR, et al. E-cigarette use, small airway fibrosis, and constrictive bronchiolitis. NEJM Evid. 2022;1(6):evidoa2100051. doi: 10.1056/evidoa2100051 37122361 PMC10137322

[5] Pierce JP, Chen R, Leas EC, White MM, Kealey S, Stone MD, et al. Use of e-cigarettes and other tobacco products and progression to daily cigarette smoking. Pediatrics. 2021;147(2):e2020025122. doi: 10.1542/peds.2020-025122 33431589 PMC7849197

[6] Khouja JN, Suddell SF, Peters SE, Taylor AE, Munafò MR. Is e-cigarette use in non-smoking young adults associated with later smoking? A systematic review and meta-analysis. Tob Control. 2020;30(1):8–15. doi: 10.1136/tobaccocontrol-2019-055433 32156694 PMC7803902

[7] Everard CD, Silveira ML, Kimmel HL, Marshall D, Blanco C, Compton WM. Association of Electronic Nicotine Delivery System use with cigarette smoking relapse among former smokers in the United States. JAMA Netw Open. 2020;3(6):e204813. doi: 10.1001/jamanetworkopen.2020.4813 32501492 PMC7275247

[8] Gomajee R, El-Khoury F, Goldberg M, Zins M, Lemogne C, Wiernik E, et al. Association between electronic cigarette use and smoking reduction in France. JAMA Intern Med.2019;179(9):1193–1200. doi: 10.1001/jamainternmed.2019.1483 31305860 PMC6632120

[9] Bhatta DN, Glantz SA. Association of e-cigarette use with respiratory disease among adults: a longitudinal analysis. Am J Prev Med. 2020;58(2):182–90. doi: 10.1016/j.amepre.2019.07.028 31859175 PMC6981012

[10] Hair EC, Barton AA, Perks SN, Kreslake J, Xiao H, Pitzer L, et al. Association between e-cigarette use and future combustible cigarette use: evidence from a prospective cohort of youth and young adults, 2017–2019. Addict Behav. 2021;112:106593. doi: 10.1016/j.addbeh.2020.106593 32927247

[11] El Asmar ML, Laverty AA, Vardavas CI, Filippidis FT. How do Europeans quit using tobacco, e-cigarettes and heated tobacco products? A cross-sectional analysis in 28 European countries. BMJ Open. 2022;12(4):e059068. doi: 10.1136/bmjopen-2021-059068 35487758 PMC9058771

[12] Filippidis FT, Laverty AA, Mons U, Jimenez-Ruiz C, Vardavas CI. Changes in smoking cessation assistance in the European Union between 2012 and 2017: pharmacotherapy versus counselling versus e-cigarettes. Tob Control. 2019;28(1):95–100. doi: 10.1136/tobaccocontrol-2017-054117 29563220 PMC6317445

[13] Pasquereau A, Andler R, Guignard R, Soullier N, Gautier A, Richard JB, Nguyen-Thanh V. Consommation de tabac parmi les adultes en 2020: résultats du Baromètre de Santé publique France. Bull Epidemiol Hebd. 2021;(8):132–9. Available at: http://beh.santepubliquefrance.fr/beh/2021/8/2021_8_1.html

[14] European Commission. Tobacco—Product regulation. Available from: https://ec.europa.eu/health/tobacco/products_en. Accessed 27 November 2020.

[15] Dai X, Gakidou E, Lopez AD. Evolution of the global smoking epidemic over the past half century: strengthening the evidence base for policy action. Tob Control. 2022;31(2):129–37. doi: 10.1136/tobaccocontrol-2021-056535 35241576

[16] Creswell JW, Clark VLP. Designing and conducting mixed methods research. Thousand Oaks, CA, US: Sage Publications, Inc; 2007.

[17] von Elm E, Altman DG, Egger M, Pocock SJ, Gøtzsche PC, Vandenbroucke JP, et al. Strengthening the Reporting of Observational Studies in Epidemiology (STROBE) statement: guidelines for reporting observational studies. BMJ. 2007;335(7624):806–8. doi: 10.1136/bmj.39335.541782.AD 17947786 PMC2034723

[18] Tong A, Sainsbury P, Craig J. Consolidated criteria for reporting qualitative research (COREQ): a 32-item checklist for interviews and focus groups. Int J Qual Health Care. 2007;19(6):349–57. doi: 10.1093/intqhc/mzm042 17872937

[19] Deville JC, Särndal CE, Sautory O. Generalized raking procedures in survey sampling. J Am Stat Assoc. 1993;88(423):1013–20.

[20] INSEE. La macro SAS CALMAR. Available from: https://www.insee.fr/fr/information/2021902. Accessed 28 november 2020.

[21] Braun V, Clarke V. Using thematic analysis in psychology. Qual Res Psychol. 2006;3(2):77–101.

[22] Corbin J, Strauss A. Basics of qualitative research: Techniques and procedures for developing grounded theory. 3rd edition. Thousand Oaks, CA, US: Sage Publications, Inc; 2008.

[23] Glaser BG, Strauss AL. The discovery of grounded theory: strategies for qualitative research. 5th edition. New Brunswick, New Jersey: Aldine Transaction; 2010.

[24] Gravely S, Driezen P, Ouimet J, Quah ACK, Cummings KM, Thompson ME, et al. Prevalence of awareness, ever-use and current use of nicotine vaping products (NVPs) among adult current smokers and ex-smokers in 14 countries with differing regulations on sales and marketing of NVPs: cross-sectional findings from the ITC Project. Addiction. 2019;114(6):1060–73. doi: 10.1111/add.14558 30681215 PMC6510648

[25] Du Y, Liu B, Xu G, Rong S, Sun Y, Wu Y, et al. Association of electronic cigarette regulations with electronic cigarette use among adults in the United States. JAMA Netw Open. 2020;3(1):e1920255. doi: 10.1001/jamanetworkopen.2019.20255 32003818 PMC7042861

[26] Lee C, Yong HH, Borland R, McNeill A, Hitchman SC. Acceptance and patterns of personal vaporizer use in Australia and the United Kingdom: Results from the International Tobacco Control survey. Drug Alcohol Depend. 2018;185:142–8. doi: 10.1016/j.drugalcdep.2017.12.018 29448147 PMC5889728

[27] La Torre G, Mipatrini D. Country-level correlates of e-cigarette use in the European Union. Int J Public Health. 2016;61(2):269–75. doi: 10.1007/s00038-016-0792-1 26874833

[28] Vardavas CI, Agaku IT. Electronic cigarettes: the Issues behind the moral quandary. In: Loddenkemper R, Kreuter M, editors. Progress in Respiratory Research. Basel: Karger; 2015. Vol. 42; p. 258–67.

[29] Pokhrel P, Herzog TA, Muranaka N, Fagan P. Young adult e-cigarette users’ reasons for liking and not liking e-cigarettes: A qualitative study. Psychol Health. 2015;30(12):1450–69. doi: 10.1080/08870446.2015.1061129 26074148 PMC4657726

[30] Selya AS, Rose JS, Dierker L, Hedeker D, Mermelstein RJ. Evaluating the mutual pathways among electronic cigarette use, conventional smoking and nicotine dependence. Addiction. 2018;113(2):325–33. doi: 10.1111/add.14013 28841780 PMC5760290

[31] Farrimond H. A typology of vaping: identifying differing beliefs, motivations for use, identity and political interest amongst e-cigarette users. Int J Drug Policy. 2017;48:81–90. doi: 10.1016/j.drugpo.2017.07.011 28810158

[32] Notley C, Ward E, Dawkins L, Holland R. User pathways of e-cigarette use to support long term tobacco smoking relapse prevention: a qualitative analysis. Addiction. 2021;116(3):596–605. doi: 10.1111/add.15226 33463849

[33] McCausland K, Jancey J, Leaver T, Wolf K, Freeman B, Maycock B. Motivations for use, identity and the vaper subculture: a qualitative study of the experiences of Western Australian vapers. BMC Public Health. 2020;20(1):1552. doi: 10.1186/s12889-020-09651-z 33059652 PMC7559168

[34] Tokle R, Pedersen W. ‘Cloud chasers’ and ‘substitutes’: e-cigarettes, vaping subcultures and vaper identities. Sociol Health Illn. 2019;41(5):917–32. doi: 10.1111/1467-9566.12854 30677161

[35] Hoek J, Thrul J, Ling P. Qualitative analysis of young adult ENDS users’ expectations and experiences. BMJ Open. 2017;7(3):e014990. doi: 10.1136/bmjopen-2016-014990 28270392 PMC5353280

[36] Yule JA, Tinson JS. Youth and the sociability of ‘Vaping’. J Consum Behav. 2017;16(1):3–14.

[37] Baig SA, Giovenco DP. Behavioral heterogeneity among cigarette and e-cigarette dual-users and associations with future tobacco use: Findings from the Population Assessment of Tobacco and Health Study. Addict Behav. 2020;104:106263. doi: 10.1016/j.addbeh.2019.106263 32028096 PMC7092617

[38] Niaura R, Rich I, Johnson AL, Villanti AC, Romberg AR, Hair EC, et al. Young adult tobacco and e-cigarette use transitions: examining stability using multistate modeling. Nicotine Tob Res. 2020;22(5):647–54. doi: 10.1093/ntr/ntz030 30820566

[39] Sweet L, Brasky TM, Cooper S, Doogan N, Hinton A, Klein EG, et al. Quitting behaviors among dual cigarette and e-cigarette users and cigarette smokers enrolled in the tobacco user adult cohort. Nicotine Tob Res. 2019;21(3):278–84. doi: 10.1093/ntr/nty222 30346585 PMC6379027

