# Supplementary material for: Prevalence, lived experiences and user profiles in e-cigarette use: A mixed methods study among French college students
Source: PLoS One. 2024 Feb 9;19(2):e0297156. doi: 10.1371/journal.pone.0297156 (PMC10857705; doi:10.1371/journal.pone.0297156)
Supplement: S1 Table — *IQR: Interquartile range; αData collected in the online quantitative study on e-cigarette use; βData collected in qualitative research among former and current smokers who had used e-cigarettes for at least two continuous months. (PDF) [file pone.0297156.s001.pdf]

S1 Table. Vaping among college students who had tried e-cigarettes in a quantitative phase [α] (N=704) and qualitative phase [β] (N=20) from a mixed methods study carried out at the University of Bordeaux (France), 2016-2017

**Characteristics**	**n**	**% unweighted**	**Representative quotes about reasons for using e-cigarettes [β]**
**Experiment with e-cigarettes**			
Age of first e-cigarette test in years, n=704 (median, IQR*) [α]	20	18 - 21	
Reasons for trying e-cigarettes, in multiple choice (n=704)			
• Curiosity [α]	542	77.0	*"It was mostly out of curiosity […] It was 3 years ago, it was really more for uh ... to play, to see what it was like. A friend had one, he gave me a try"* (Sam: man, former tobacco smoker and e-cigarette user for 3 years)
• Someone offered a try [α]	450	63.9	
• Because of flavors [α]	163	23.2	
• To quit (or try to quit) smoking [α]	74	10.5	*"I wanted to quit smoking because I smoked too much […] and then I imagined that with a gradual decrease of nicotine it would be easier to quit"* (Julie: woman, former tobacco smoker and e-cigarette user for 1.5 years)
• To reduce smoking but without stopping [α]	60	8.5	*"When I switched to e-cig, I didn't necessarily want to quit smoking, it wasn't necessarily my first desire. It was more to move on, ... to not to smell of tobacco anymore"* (Ann: woman, former tobacco smoker and e-cigarette user for 1.5 years)
• E-cigarettes are less harmful to health than tobacco [α]	51	7.2	*"At the time, I thought it was good because, it was less harmful to other people's health, or ... and mine too"* (Ce: woman, dual user who had stopped smoking and then relapsed while continuing to vape)
• E-cigarettes can be used in places where smoking is prohibited [α]	47	6.7	
• E-cigarettes are cheaper than smoking tobacco [α]	43	6.1	*"This is really the main reason: it's more expensive to buy the first time, but in the long run, it's clearly cheaper. That's really what attracted me "* (Téo Da: man, former tobacco smoker and e-cigarette user for 3 years)

• Others reasons $^{\beta}$: keeping gestures of the smoking act, less discomfort for relatives, discreet use that can be hidden from parents, failure of nicotine replacement therapies, playful use	/	/	*"The other ways to quit, I was not convinced, let's say. […] I tried gum: I didn't like the taste; I didn't like the feel. And I think switching from cigarettes to e-cigarettes was the easiest"* (Ann: woman, former smoker and e-cigarette user for 1.5 years).
**Current use of e-cigarettes**			
Frequency of e-cigarette current use (n=58) $^{\alpha}$			
• Occasionally (<1 per day)	28	48.3	
• Daily (≥ 1 per day)	30	51.7	
Reasons to currently use e-cigarettes, in multiple choice (n=58)			
• E-cigarettes can be used in places where smoking is prohibited $^{\alpha}$	21	36.2	*"For example, when I am in a bar, and everyone goes out to smoke, I can stay inside with my electronic cigarette and the non-smokers"* (Ce: woman, dual user who had stopped smoking and then relapsed while continuing to vape)
• To never start smoking $^{\alpha}$	19	32.8	
• To take few puffs without having to finish a cigarette $^{\alpha}$	19	32.8	*"What I like about them? Being able to use them more, 'to be freer to use them at home or in other places, and not have to stay 5 minutes to finish my ciggy or say "I'm not going in there, I don't want to waste it", I need, I only need 2 drags, but nothing will be wasted …"* (Ann: woman, former smoker and e-cigarette user for 1.5 years)
• To relieve an urge to smoke in certain circumstances $^{\alpha}$	18	31.0	*"With that, even if you want a cigarette [...] you will pull two or three drags on your e-cig and it will pass. You're not going to crack"* (Tim Bo: man, dual user for 4 months)
• E-cigarettes are cheaper than smoking tobacco $^{\alpha}$	14	24.1	"*It is much cheaper for sure. I smoked a pack of cigarettes a day so it can hardly be more expensive ..."* (Paul: man, dual user for 3 months)
• The throat hit (sensation felt in the throat after a drag) during vaping is more intense than with tobacco $^{\alpha}$	13	22.4	"*I like it when it's quite dense when there is a big smoke, ... that there is a slight hit, this thing that keeps me from constantly shooting on*" (Tao: man, former smoker and e-cigarette user for 4 months)

• To quit smoking $^{\alpha}$	13	22.4	"*At the beginning I wanted to say "I'm proud of myself", but it was finally so easy that I didn't feel I had achieved much*" (Ann: woman, former smoker and e-cigarette user for 1.5 years)
• E-cigarettes are less addictive than tobacco $^{\alpha}$	13	22.4	"*This is not a new addiction, it is the continuation of a previous addiction by trying to make it less harmful*" (Paul: man, dual user for 3 months)
• Because of flavors $^{\alpha}$	11	19.0	*"You discuss in store with the seller what you want like taste: rather fruity, rather greedy rather, which resembles tobacco, etc. He will make you taste different e-liquids ... and after that, you take what you prefer"* (Tim Bo: man, dual user for 4 months)
• E-cigarettes are less harmful to health than tobacco $^{\alpha}$	11	19.0	"*I tell myself it's necessarily less bad. Now the future will tell if this is the case ... Personally, I am convinced that it cannot be worse than cigarettes*" (Paul: man, dual user for 3 months)
• For the conviviality of vaping (break time with tobacco smokers, meeting with e-cigarette users, keeping contact with never smokers) $^{\alpha}$	9	15.5	
• To reduce smoking but without stopping $^{\alpha}$	8	13.8	
• To avoid going outside when I want to smoke $^{\alpha}$	7	12.1	*"It's true that [...] where I work, you can vape in the rest room, whereas ... to smoke, you have to go out ..."* (Tim Bo: man, dual user for 4 months)
• Others reasons $^{\beta}$: disappearance of the physical inconveniences of smoking, enjoyment to use, maintenance of smoking habits, short-term improvement in physical health, convenience and availability, better social acceptance than smoking, ease of personalized use, weight control during smoking cessation, psychotropic effects (aid in concentration, relaxing effect)	/	/	"*No longer having your clothes or hair that smell like cigarettes at night is really: yeah! That's above all what felt good*" (Ann: woman, former smoker and e-cigarette user for 1.5 years)
Use of a personal electronic device (n=58) $^{\alpha}$	56	96.6	
Kind of e-liquids used (n=58) $^{\alpha}$			
• With nicotine	34	58.6	
• Without nicotine	11	19.0	
• Both	8	13.8	

• I don't know	5	8.6	
Vaping places, in multiple choice (n=58) [α]			
• Home	47	81.0	
• With friends or family	40	69.0	
• Outside	33	56.9	
• Inside cafes, restaurants, bars or on their terraces	33	56.9	
• At university	30	51.7	
• In the car	30	51.7	
• At work place and in professional meetings	11	19.0	
• Inside nightclubs and their terraces	11	19.0	
Vaping times (n=58) [α]			
• Weekends (Saturday and Sunday)	20	34.5	
• Weekdays (Monday to Friday)	3	5.2	
• Both	35	60.3	

[*] IQR: interquartile range; [α]Data collected in the online quantitative study on e-cigarette use; [β]Data collected in qualitative research among former and current smokers who had used e-cigarettes for at least two continuous months.
