# Supplementary material for: Prevalence, lived experiences and user profiles in e-cigarette use: A mixed methods study among French college students
Source: PLoS One. 2024 Feb 9;19(2):e0297156. doi: 10.1371/journal.pone.0297156 (PMC10857705; doi:10.1371/journal.pone.0297156)
Supplement: S1 File — (PDF) [file pone.0297156.s002.pdf]

Guide final en français

***- Présentation : pour commencer,** pourriez-vous vous présenter en quelques mots, votre prénom, votre âge, le domaine d'étude (Filière d'études à faire préciser si spontanément l'étudiant ne la signale pas en se présentant)* $^{\alpha}$
***- Question brise-glace :** si votre e-cigarette était un animal/un personnage, lequel serait-il ?* $^{\alpha}$

1/Le premier contact avec l'e-cigarette

- Comment avez-vous connu l'existence de la cigarette électronique ?

- Dans quel contexte (environnemental, social, psychologique) avez-vous été amené à l'expérimenter la 1 ère fois ?

- Quelles ont été les premières sensations dès la 1ère utilisation ?

- Qu'est-ce qui vous a attiré dans ce produit (éléments d'influence positive) ?

- Quelqu'un de votre entourage (famille, ami) utilisait-il une e-cigarette ?
  - Si oui, quelle influence de l'usage de cet entourage sur l'initiation personnelle ?

2/L'usage de tabac chez les vapoteurs

- Aviez-vous déjà fumé du tabac avant d'utiliser l'e-cigarette ?

- Aujourd'hui, utilisez-vous seulement du tabac, seulement l'e-cigarette, les deux ? Pourquoi ?

- Qu'est ce qui est différent entre la cigarette de tabac et l'e-cigarette (dans le geste, les habitudes, dans les sensations ressenties, etc.) ?

- Avez-vous déjà ressenti un manque (physique, comportemental ou psychologique) par rapport au tabac depuis que vous vapotez ? Dans quelles circonstances ?

→P<u>our les anciens fumeurs :</u>

- Pourquoi avez-vous favorisé l'e-cigarette au tabac (possibilité d'être acteur/personnaliser son utilisation : saveur, taux de nicotine, puissance du dispositif, ...) ?

- Au bout de combien de temps (à partir de la 1ère utilisation) avez-vous favorisé l'usage d'e-cigarette ? Pendant combien de temps ?

- Avant l'e-cigarette, vous aviez déjà tenté d'arrêter de fumer ? Avec quoi (sans aide, patchs, gommes, pastilles, varénicline, etc.) ? Quels avantages de la e-cigarette par rapport à ces expériences antérieures (explorer si ce n'est annoncé spontanément s'il y a un désir de contrôle du poids à l'arrêt du tabac par l'e-cigarette $^{\gamma}$) ? Quels inconvénients ?

3/L'usage actuel d'e-cigarette

- Comment vous-servez-vous de votre e-cigarette maintenant : quand (A quels moments vous l'utilisez dans la journée) ? Comment ? Où ? A quelle fréquence ? Et dans quel but (recherche de quels effets) ? (explorer la co-consommation avec l'alcool dans certaines circonstances) $^{\gamma}$

- Comment cet usage a-t-il évolué depuis le début (taux de nicotine, changement de matériel, etc.) ? $^{\alpha}$

- Comment vous approvisionnez-vous (origine de l'e-liquide, de la e-cigarette, etc.) ? $^{\beta}$

- De quoi est fait votre e-liquide (connaissance de la composition) ? $^{\beta}$

- Au fur et à mesure de l'évolution des e-cigarettes, pensez-vous que leur prix a une signification vis-à-vis des performances ou de leur qualité ? $^{\alpha}$

- (Avez-vous l'impression que) vous pourriez vous passer de l'e-cigarette ?

→ Pour les fumeurs (= vapoteurs dualistes) :

- Envisagez-vous de poursuivre l'utilisation du tabac ou, au contraire, d'arrêter à un moment donné ? Pourquoi ?

- Envisagez-vous de poursuivre l'utilisation d'e-cigarette ou, au contraire, d'arrêter à un moment donné ? Pourquoi ?

- Quelles différences/quels effets en passant de la cigarette à la e-cigarette (ou inversement) au niveau physique, psychologique (satisfaction, estime de soi, confiance...) ?

4/L'accès à l'information sur l'e-cigarette : passif (environnement/influence) OU actif (étudiant actif dans sa recherche)

- Connaissez-vous des publicités concernant ce produit ? Où les avez-vous vus ?

- Qu'est-ce que la publicité vous a appris concernant la e-cigarette ?

- Après/pendant la pub, votre utilisation de la e-cigarette ou ses composants a changé ?

  • Si oui, comment (achat d'une nouvelle saveur, modèle de e-cigarette, l'envie de fumer en voyant la pub...) ?

- Comment obtenez-vous des informations sur la e-cigarette ?

5/La vision de l'e-cigarette

- Conseillerez-vous l'utilisation de l'e-cigarette à quelqu'un ? Pourquoi ?

- Que diriez-vous à une personne qui s'interroge sur le fait d'utiliser ou non la e-cigarette ?

→Pour les fumeurs (= vapoteurs dualistes) : en utilisant l'e-cigarette, vous considérez vous fumeur (pourquoi) ? Vous-considérez-vous vapoteur (pourquoi) ?

→Pour les anciens fumeurs : est-ce que vous vous considérez (toujours) comme fumeur ? Vous-considérez-vous vapoteur (pourquoi) ?

- Pour vous, qu'est-ce finalement être un vapoteur (identité de vapoteur) ? [γ]

- Que pense votre entourage de l'e-cigarette ?

- Clôture de l'entretien

Notre discussion est terminée. Auriez—vous des choses à rajouter ?

Une nouvelle fois, merci d'avoir pris le temps d'échanger avec nous sur votre expérience.

Notes au sujet de l'évolution du guide

- Guide initial préparé en 2015 par SK et l'équipe de recherche, testé avant le premier entretien auprès d'étudiants (non inclus dans l'étude) et des vendeurs dans une boutique spécialisée de produits du vapotage.
- [α] Questions rajoutées après analyse des entretiens 1 et 2
- [β] Questions rajoutées après analyse de l'entretien 3
- [γ] Questions rajoutées après analyse des entretiens 4, 5, 6, 7 et 8
