# Supplementary material for: Prevalence, lived experiences and user profiles in e-cigarette use: A mixed methods study among French college students
Source: PLoS One. 2024 Feb 9;19(2):e0297156. doi: 10.1371/journal.pone.0297156 (PMC10857705; doi:10.1371/journal.pone.0297156)
Supplement: S2 File — (PDF) [file pone.0297156.s003.pdf]

Final guide in English

- ***Presentation:*** *to start, could you introduce yourself in a few words, your first name, your age, the field of study (The field of study must be specified if the student does not spontaneously indicate it when introducing himself)* [α]
- ***Icebreaker question:*** *if your e-cigarette was an animal/character, which one would it be? Why?* [α]

1/The first contact with the e-cigarette

- How did you hear about the electronic cigarette?

- In what context (environmental, social, psychological) did you first experience it?

- What were the first sensations after the first use?

- What attracted you in this product (elements of positive influence)?

- Did anyone around you (family, friend) use an e-cigarette?

  - If so, what influence does the use of this environment have on personal initiation?

2/ Tobacco use among vapers

- Had you ever smoked tobacco before using the e-cigarette?

- Today, do you only use tobacco, only e-cigarettes, both? Why?

- What is different between the tobacco cigarette and the e-cigarette (in the gesture, the habits, in the sensations felt, etc.)?

- Have you ever felt withdrawal (physical, behavioral or psychological) in relation to tobacco since you vaped? In which circumstances?

→ <u>For former smokers:</u>

- Why did you choose the e-cigarette over tobacco (possibility of being an actor/customizing its use: flavor, nicotine level, device power, etc.)?

- After how long (from the first use) did you favour the use of e-cigarettes? For how long?

- Before the e-cigarette, had you ever tried to quit smoking? With what (without help, patches, gums, lozenges, varenicline, etc.)? What are the advantages of the e-cigarette compared to these previous experiences (explore if not spontaneously announced if there is a desire to control weight when quitting smoking with the e-cigarette [γ])? What disadvantages?

3/ Current use of e-cigarettes

- How do you use your e-cigarette now: when (at what times do you use it during the day)? How? Where? How often? And for what purpose (what effects are you looking for)? (explore co-consumption with alcohol in certain circumstances [γ])

- How has this use evolved since the beginning (nicotine level, change of device, etc.)? [α]

- How do you get your supplies (origin of the e-liquid, the e-cigarette, etc.)? [β]

- What is your e-liquid made of (knowledge of the composition)? [β]

- As e-cigarette evolve, do you think their price has any significance in terms of performance or quality? [α]

- (Do you feel that) you could do without the e-cigarette?

→ For smokers (= dual vapers):

- Do you plan to continue using tobacco or, on the contrary, to stop at some point? Why?

- Do you plan to continue using e-cigarettes or, on the contrary, to stop at some point? Why?

- What are the differences/ effects of switching from cigarettes to e-cigarettes (or vice versa) at the physical and psychological level (satisfaction, self-esteem, confidence, etc.)?

4/ Access to information on e-cigarettes: passive (environment/influence) OR active (student active in research)

- Do you know of any advertisements for this product? Where have you seen them?

- What have you learned about e-cigarettes from advertising?

- After/during the advert, has your use of the e-cigarette or its components changed?

    - If so, how (purchase of a new flavour, e-cigarette device, the urge to smoke when seeing the advert, etc.)?

- How do you get information on the e-cigarette?

5/The vision of the e-cigarette

- Would you advise anyone to use an e-cigarette? Why?

- What would you say to someone who is considering whether or not to use the e-cigarette?

→ For smokers (= dual vapers): when using the e-cigarette, do you consider yourself a smoker (why)? Do you consider yourself a vaper (why)?

→ For former smokers: do you (still) consider yourself to be a smoker? Do you consider yourself a vaper (why)?

- What does it ultimately mean to you to be a vaper (vaper identity)? [γ]

- What do those around you think about e-cigarettes?

- Closing the interview

Our discussion is over. Would you have anything to add?

Once again, thank you for taking the time to share your experience with us.

Notes on the evolution of the guide

- Initial guide prepared in 2015 by SK and the research team, tested before the first interview with students (not included in the study) and with sellers in a shop specializing in vaping products.
- [α] Questions added after analysis of interviews 1 and 2
- [β] Questions added after analysis of interview 3
- [γ] Questions added after analysis of interviews 4, 5, 6, 7 and 8
