# Supplementary material for: Prevalence, lived experiences and user profiles in e-cigarette use: A mixed methods study among French college students
Source: PLoS One. 2024 Feb 9;19(2):e0297156. doi: 10.1371/journal.pone.0297156 (PMC10857705; doi:10.1371/journal.pone.0297156)
Supplement: S3 File — (PDF) [file pone.0297156.s004.pdf]

i-Share

Internet-based **S**tudents **H**e**A**lth **R**esearch **E**nterprise

Questionnaire d'inclusion-

Qui suis-je ?

Mes études

q1. Baccalauréat obtenu

- ❒ Bac L
- ❒ Bac technologique
- ❒ Bac ES
- ❒ Bac professionnel
- ❒ Bac S
- ❒ Equivalent

q2. Année d'obtention du baccalauréat : |__|__|__|__|

q3. Vous êtes actuellement inscrit(e) à l'Université de (Université indiquée lors de l'inscription), merci de préciser votre filière d'inscription :

(affichage conditionné en fonction de l'université renseignée au moment de l'inscription en ligne))

❒ Université Bordeaux 1 Sciences et technologies

- Préciser la filière d'inscription :
  ❒ biologie ❒ chimie ❒ informatique ❒ mathématiques
  ❒ mathématiques appliquées ❒ physique Chimie ❒ Physique et ingénieries ❒ Sciences de la terre et de l'environnement
  ❒ Sciences exactes et naturelles ❒ Autre

❒ Université Bordeaux Segalen

- Préciser la filière d'inscription :
  ❒ Santé (❒ médecine / ❒ pharmacie / ❒ odontologie / ❒ PACES (1ère année commune aux études de santé)) ❒ Sciences de l'homme
  ❒ Sciences de la vie ❒ Sciences et modélisation ❒ STAPS
  ❒ Sciences humaines ❒ Psychologie ❒ Sociologie
  ❒ Autre

❒ Université Michel de Montaigne Bordeaux III

- Préciser la filière d'inscription :
  ❒ aménagement et urbanisme ❒ archéologie ❒ arts
  ❒ culture humaniste et scientifique ❒ histoire de l'art
  ❒ géographie ❒ histoire ❒ information et communication
  ❒ langues et civilisations ❒ langues étrangères appliquées
  ❒ lettres ❒ philosophie ❒ sciences du langage

❒ Autre

❒ Université Montesquieu Bordeaux IV

- Préciser la filière d'inscription :
  ❒ droit ❒ sciences politiques (IEP Institut d'études politiques)
  ❒ économie ❒ gestion ❒ éducation
  ❒ Autre

❒ Université Versailles Saint-Quentin, site de Versailles, UFR Sciences

- Préciser la filière d'inscription :
  ❒ Biologie ❒ Biologie/environnement ❒ chimie ❒ informatique
  ❒ Mathématiques ❒ mathématiques appliquées
  ❒ Physique ❒ Sciences pour l'ingénieur ❒ Sciences sociales
  ❒ Autre

❒ UVSQ, site de Guyancourt, UFR Sciences de la santé

- Préciser la filière d'inscription :
  ❒ Santé : ❒ médecine / ❒ pharmacie / ❒ odontologie / ❒ PACES (1ère année commune aux études de santé)

❒ UVSQ, site de Guyancourt, UFR Sciences sociales

- Préciser la filière d'inscription :
  ❒ Géographie ❒ Sociologie ❒ Economie Gestion
  ❒ Administration économique et sociale
  ❒ Licence professionnelle ABF ❒ Licence professionnelle Hôtellerie/Tourisme
  ❒ Autre

❒ UVSQ, site de Guyancourt, Faculté de droit et de sciences politiques

- Préciser la filière d'inscription :
  ❒ Droit ❒ Science politique
  ❒ Autre

❒ UVSQ, site de Guyancourt, Institut d'études culturelles

- Préciser la filière d'inscription :
  ❒ Histoire ❒ Lettres modernes ❒ LLCE Espagnol
  ❒ Musiques anciennes et monde contemporain
  ❒ Autre

❒ UVSQ, site de Guyancourt, Institut des langues et des études internationales (ILEI)

- Préciser la filière d'inscription :

  ❒ Droit et Anglais ❒ Sciences économiques/langues
  ❒ Licence études européennes et internationales (LEEI)
  ❒ Licence LLCE, Anglais

❒ Autre

❒ UVSQ, site deGuyancourt, Institut supérieur de management (ISM)

- Préciser la filière d'inscription :

  ❒ Gestion des ressources humaines (GRH) ❒ Sciences de gestion

  ❒ Autre

❒ IUT de Mantes en Yvelines, Mantes-la-Jolie

❒ Institut des sciences et techniques des Yvelines (ISTY), Vélizy

❒ IUT de Vélizy et son antenne de Rambouillet, Vélizy-Villacoublay

❒ Observatoire des sciences de l'univers de l'UVSQ (OVSQ), Guyancourt

q4. Année d'étude en cours :

❒ L1 (1°année IUT, PACES…)

❒ L2 (2°année IUT, PCEM2…)

❒ L3

❒ Autre

Précisez : ……………………………….

q5. Avant ce cursus, avez-vous entrepris des études dans une autre filière ?

❑ Oui ❑ Non

►**Si oui: étiez-vous en :**

❒ Droit- Sciences Politiques ❒ Economie ❒ Sciences Humaines

❒ Sciences ❒ STAPS ❒ Lettres ❒ Santé

❒ Classes préparatoires (CPGE)

❒ Autre Précisez : ……………………………….

q6. Précisez le niveau d'étude que vous souhaitez atteindre ?

❒ Licence

❒ Master

❒ Doctorat

❒ Autre

Ma famille, …, ma vie avant

q7. Avez-vous un ou plusieurs sœurs et frères ?

❒ Oui ❒ Non ❑ Ne souhaite pas répondre

►**Si oui**:

Nombre de frères: |__|__|

Nombre de sœurs: |__|__|

q8. Vos parents ont-ils divorcé, ou se sont-ils séparés ?

❑ Oui ❑ Non ❑ Ne souhaite pas répondre

►**Si oui**, pourriez-vous préciser votre âge au moment du divorce ou de la séparation :

|__|__| ans (*indiquez « 0 » si cela s'est produit avant votre naissance ou au cours de votre 1ère année de vie)*

q9. Pendant votre enfance et votre adolescence, avez-vous vécu principalement:

- ❑ Chez vos (ou l'un de vos) parents (ou parents adoptifs)
- ❑ Chez vos grands-parents
- ❑ Chez d'autres membres de votre famille (oncles, tantes, …)
- ❑ En famille d'accueil
- ❑ En Foyer
- ❑ Autre
- ❑ Ne souhaite pas répondre

q10. Pendant votre enfance et votre adolescence, vous êtes-vous senti soutenu et réconforté par votre famille ?

- ❑ Pas du tout
- ❑ Un peu
- ❑ Modérément
- ❑ Beaucoup
- ❑ Enormément
- ❑ Ne souhaite pas répondre

q11. Pouvez-vous indiquer le niveau d'études du ou des adulte(s) qui vous ont élevé(e) ?

(Vous pouvez ne compléter qu'une seule colonne)	**Adulte 1**	**Adulte 2**
**Ne sais pas**	❑	❑
**Ecole primaire**	❑	❑
**Etudes secondaires jusqu'au brevet**	❑	❑
**Etudes secondaires après brevet jusqu'au bac**	❑	❑
**Etudes supérieures post BAC**	❑	❑
**Diplômes professionnels (CAP, BEP)**	❑	❑

q12. Diriez-vous que la situation économique de la famille qui vous a élevé(e) durant votre enfance et adolescence était

❑ Très confortable
❑ Confortable
❑ Correcte
❑ Difficile
❑ Très difficile

q13. Durant votre enfance et/ou adolescence, avez-vous eu un animal de compagnie ?

❑ Oui ❑ Non

► Si oui, pourriez-vous préciser :

❑ Chat
❑ Chien
❑ Rongeur
❑ Autre

Mes conditions de vie aujourd'hui

q14. Actuellement, où vivez-vous ?

❒ Chez vos parents, ou l'un de vos parents
❒ En résidence universitaire ou en foyer
❒ Dans un appartement
Précisez :
❒ En couple
❒ Seul
❒ En colocation
❒ Autre Précisez : ………………………

q15. Avez-vous des enfants ?

❑ Oui ❑ Non ❑ Ne souhaite pas répondre

q16. A quelle fréquence rentrez-vous chez vos parents au cours de l'année universitaire ?

❒ 1 fois par semaine
❒ 1 fois par mois
❒ Au cours des vacances scolaires
❒ Une ou deux fois par an
❒ Jamais
❒ Autre : précisez : …………………….

q17. Quelles sont vos différentes sources de revenus pour le financement de votre année universitaire ?

❒ Famille
❒ Bourses de l'état sur critères sociaux
Merci de préciser à quel échelon :
❒ 0 (exonération du paiement des droits universitaires et cotisation de la sécu)
❒ 1 (1606€ annuels)
❒ 2 (2419 € annuels)
❒ 3 (3100€ annuels)
❒ 4 (3779€ annuels)
❒ 5 (4339€ annuels)
❒ 6 (4600 € annuels)

❐ Ne sais pas

❑ Ne souhaite pas répondre

❐ Bourse au mérite

❐ Activité(s) rémunérée(s) (stages rémunérés, jobs d'appoints ou sous contrat, pendant l'année ou les vacances …)

❐ Autre Précisez : ………….. ……………………

(questions 18 à 20 n'apparaissent que si « Activités rémunérées » coché à la question précédente)

q18. Vos activités rémunérées vous occupent-elles au total ?

❑ A temps plein

❑ A mi-temps

❑ Moins d'un mi-temps mais régulièrement

❑ Occasionnellement

❑ Seulement au cours des vacances scolaires (notamment les mois d'été)

❑ Stages rémunérés **(**externat pour médecine)

q19. Ces activités sont-elles

❑ Votre source principale de revenus

❑ Un complément financier mais indispensable pour vivre (logement, transports, …)

❑ Un complément pour vos loisirs (sorties, sport, …)

❑ Autre

q20. Ces activités ont-elles un impact sur vos études

❑ Plutôt positif ❑ Plutôt négatif

❑ Pas d'impact particulier (Neutre)

q21. Que diriez-vous aujourd'hui sur les aspects suivants de vos conditions de vie :

	Tout à fait satisfait	Plutôt satisfait	Satisfait	Plutôt insatisfait	Tout à fait insatisfait
Vos ressources	❑	❑	❑	❑	❑
Votre logement	❑	❑	❑	❑	❑
Vos possibilités de détente (loisirs…)	❑	❑	❑	❑	❑
Votre vie sociale	❑	❑	❑	❑	❑
Vos relations avec vos parents	❑	❑	❑	❑	❑

q22. Avez-vous le permis de conduire :

- **Voiture ? (Permis B)**
- ❒ Oui ❒ Non
  - Si oui en quelle année l'avez-vous obtenu ?
  - |__|__|__|__|
- **Moto ? (permis A)**
- ❒ Oui ❒ Non
  - Si oui en quelle année l'avez-vous obtenu ?
  - |__|__|__|__|

L'informatique, la télévision …

q23. Avez-vous à disposition à votre domicile un ordinateur fixe ou portable ?

❒ Oui ❒ Non

q24. Avez-vous une tablette ?

❒ Oui ❒ Non

q25. Avez-vous un téléphone mobile type Smartphone ?

❒ Oui ❒ Non

q26. **<u>Au cours de la semaine</u>, quelle est la durée moyenne quotidienne passée :**

**Par jour de semaine**	**Jamais**	**Moins de 30 minutes**	**De 30 min à 2h00**	**De 2h00 à 4h00**	**De 4 à 8h**	**Plus de 8h**
• Devant l'ordinateur/tablette pour travailler (recherches, enseignement en ligne…)	❑	❑	❑	❑	❑	❑
• Devant l'ordinateur/ tablette pour jouer (en réseau…)	❑	❑	❑	❑	❑	❑
• Devant l'ordinateur/ tablette sur Internet : réseaux sociaux, messageries,….	❑	❑	❑	❑	❑	❑
• Devant la télévision / ou télévision via l'ordinateur (séries, films, émissions…)	❑	❑	❑	❑	❑	❑
• Devant le Smartphone (recherche en ligne, réseaux sociaux, jeux…)	❑	❑	❑	❑	❑	❑

<u>Droitier ou Gaucher ?</u>

q27. **Dans les activités suivantes, veuillez indiquer la main que vous utilisez de façon préférée, en sélectionnant la case appropriée :**

	**Toujours gauche**	**Souvent gauche**	**Indifféremment gauche / droite**	**Souvent droite**	**Toujours droite**
**Ecrire**	❑	❑	❑	❑	❑
**Dessiner**	❑	❑	❑	❑	❑
**Lancer une balle**	❑	❑	❑	❑	❑
**Couper avec des ciseaux**	❑	❑	❑	❑	❑
**Se laver les dents**	❑	❑	❑	❑	❑
**Couper avec un couteau sans fourchette**	❑	❑	❑	❑	❑
**Utiliser une cuillère**	❑	❑	❑	❑	❑
**Craquer une allumette**	❑	❑	❑	❑	❑
**Utiliser la souris d'ordinateur**	❑	❑	❑	❑	❑

q28. Veuillez indiquer la préférence manuelle de vos parents

	Droitier	Gaucher	Gaucher contrarié*	Ne sais pas
**Père**	❑	❑	❑	❑
**Mère**	❑	❑	❑	❑

**individu naturellement gaucher, mais que l'on a contraint à utiliser la main droite pour écrire.*

q29. Combien avez-vous :

- De frères gauchers (ne pas inclure les demi-frères): |__|__| ou ❑ Ne sais pas
- De sœurs gauchères (ne pas inclure les demi-sœurs): |__|__| ou ❑ Ne sais pas

Question à conditionner en fonction du nombre de frères, sœurs déclarés …

Comment ça va ?

La prise en charge de ma santé

q30. Etes-vous affilié(e) à un centre de sécurité sociale étudiante ?

❒ Oui ❒ Non ❒ Ne sais pas

►**Si oui, précisez ?**

❑ A titre payant (âge > 20 ans)
❑ A titre gratuit (âge < 20 ans)

►***Si non, pourquoi ?***

❑ Vous êtes couvert par votre travail (CPAM)
❑ Parce que vous n'avez pas fait les démarches
❑ Par manque d'information
❑ Autre, précisez

q31. Avez-vous une complémentaire santé ?

❒ Oui ❒ Non ❒ Ne sais pas

►**Si oui, s'agit-il d'une :**

❑ Mutuelle étudiante (qui gère également la sécu)
❑ Mutuelle de vos parents
❑ Aide à l'acquisition d'une mutuelle par la CPAM
❑ Une assurance privée
❑ Autre mutuelle
❑ CMU complémentaire

►**Si non, pourquoi ?**

❑ Pour raisons financières
❑ Parce que vous n'avez pas fait les démarches
❑ Par manque d'information
❑ Autre
Précisez…………………………………

Ma santé

q32. Considérez-vous qu'actuellement votre santé est

- ❑ Très bonne
- ❑ Bonne
- ❑ Moyenne
- ❑ Mauvaise
- ❑ Très mauvaise

q33. Etes-vous vacciné(e) contre :

L'hépatite B : ❒ Oui ❒ Non ❒ Ne sais pas

La rougeole, les oreillons et la rubéole: ❒ Oui ❒ Non ❒ Ne sais pas

(Pour les filles uniquement)

Le papillomavirus humain (HPV) : ❒ Oui ❒ Non ❒ Ne sais pas
(Gardasil®, Ceravix®)

q34. Avez-vous votre carnet de santé en votre possession ou vous est-il aisé de le récupérer ?

❒ Oui ❒ Non ❒ Ne sais pas

q35. Au cours de votre vie, un médecin a-t-il déjà diagnostiqué chez vous :

	**Oui**	**Non**
**De l'asthme**	❑	❑
**Des allergies**	❑	❑
**De l'eczéma**	❑	❑
**Une sclérose en plaque**	❑	❑
**Un diabète de type-I**	❑	❑
**Des migraines**	❑	❑
**Des acouphènes (sifflements auditifs)**	❑	❑
**Une colite (maladie de Crohn, rectocolite hémorragique…)**	❑	❑
**Une arthrite juvénile**	❑	❑

(Pour chaque pathologie citée, si « Oui » coché, affichage des 3 questions suivantes)

Si oui

- Quel âge aviez-vous approximativement (au moment du 1er diagnostic)

  _____ ans

- Avez-vous été pris(e) en charge pour cette maladie (suivi médical régulier, prescription de traitements médicaux ou autre) ?

  ❑ Oui ❑ Non

- Avez-vous déjà été hospitalisé(e) pour cette maladie?

  ❑ Oui ❑ Non

q36. Au cours de votre vie, un médecin a-t-il déjà diagnostiqué chez vous :

	**Oui**	**Non**
**Un syndrome d'hyperactivité dans votre enfance**	❑	❑
**Des troubles anxieux, phobie**	❑	❑
**Une anorexie et/ou boulimie**	❑	❑
**Des TOC (troubles obsessionnels compulsifs)**	❑	❑
**Une dépression**	❑	❑

(Pour chaque pathologie citée, si « Oui » coché, affichage des 3 questions suivantes)

Si oui

- Quel âge aviez-vous approximativement (au moment du 1er diagnostic)

  _____ ans

- Avez-vous été pris(e) en charge pour cette maladie (suivi médical régulier, prescription de traitements médicaux ou autre, prise en charge psychologique) ?

  ❑ Oui ❑ Non

- Avez-vous déjà été hospitalisé(e) pour cette maladie?

  ❑ Oui ❑ Non

q37. Avez-vous une ou plusieurs autre(s) maladie(s) pour laquelle (lesquelles) vous êtes pris(e) en charge ?

❒ Oui ❒ Non

►**Si oui :**

**Précisez la(es)quelle(s) :**<br>***Plusieurs lignes possibles***
**……………………………………**

(Pour chaque pathologie citée, affichage des 3 questions suivantes

Si oui

- Quel âge aviez-vous approximativement (au moment du 1er diagnostic)

  _____ ans

- La maladie nuit-elle au bon déroulement de vos études ?

  ❑ Oui ❑ Non

- La prise en charge (traitement, suivi médical…) présente-t-elle des contraintes au bon déroulement de vos études ?

  ❑ Oui ❑ Non

q38. Avez-vous déjà eu des maux de tête ?

❒ Oui ❒ Non *(Si non, question suivante = q42)*

►**Si oui :**

q39. Au cours des 12 derniers mois, avez-vous eu des maux de tête se manifestant par crises de plusieurs heures ?

❒ Oui ❒ Non *(Si non, question suivante = q42)*

►**Si oui**

q40. Au cours de ces crises :

	**Oui**	**Non**	**Ne suis pas sûr(e)**
La douleur est tellement forte qu'elle vous gêne dans vos activités en cours	❒	❒	❒
La douleur prédomine d'un seul côté de la tête	❒	❒	❒
La douleur est battante, pulsatile	❒	❒	❒
La douleur est aggravée par des activités physiques comme marcher ou monter des escaliers	❒	❒	❒
Vous avez des nausées ou des maux d'estomac	❒	❒	❒
La lumière ou le bruit aggrave votre mal de tête	❒	❒	❒

q41. Quelque fois un mal de tête est associé avec des troubles visuels ou un engourdissement de certaines parties de votre corps, typiquement au début des crises. Avez-vous ressenti de tels troubles ?

❒ Oui ❒ Non

q42. Vous a-t-on déjà diagnostiqué un problème de dyslexie ?

❒ Oui ❒ Non

►**Si oui** :

Avez-vous suivi des séances d'orthophonie ?
❒ Oui ❒ Non

Bénéficiez-vous d'un aménagement des examens ?
❒ Oui ❒ Non

q43. Etes-vous porteur(se) d'un handicap ?

❒ Oui ❒ Non

►**Si oui** :

Précisez lequel :

❒ Handicap visuel ❒ Handicap auditif

❒ Handicap mental et psychique ❒ Handicap moteur

❒ Autre handicap, précisez : ……………………………..

Cela nuit-il au bon déroulement de vos études ?
❒ Oui ❒ Non

Bénéficiez-vous d'un aménagement des examens ?
❒ Oui ❒ Non

Mes évènements de santé récents

q44. Au cours des 12 derniers mois, avez-vous eu à plusieurs reprises les infections suivantes :

- rhinopharyngite (rhume) ❒ oui ❒ non
- angine ❒ oui ❒ non
- grippe ou syndrome grippal ❒ oui ❒ non
- gastroentérite ❒ oui ❒ non

q45. Avez-vous été pris(e) en charge à l'hôpital ou dans une clinique <u>au cours des derniers 12 mois</u> ?

❐ Oui ❐ Non

►**<u>Si oui,</u> précisez dans le tableau suivant :**

<u>(Autant de lignes pourront être affichées que le volontaire aura de prises en charge à déclarer)</u>

La cause	Le lieu	La durée totale pour cet évènement (quelques soient les différents services)
❐ Accident de sport ❐ Accident de la route ❐ Accident domestique<br>❐ Accident de loisirs<br>❐ Agression<br>❐ Maladie (infection, maladie chronique…)<br>❐ Autre ; Précisez : ………………….	❐ Urgences<br>❐ Urgences psychiatriques<br>❐ Service médical<br>❐ Service chirurgical<br>❐ Clinique	❐ Moins d'une journée<br>❐ Une journée<br>❐ De 1 à 3 jours<br>❐ De 3 jours à une semaine<br>❐ Plus d'une semaine

<u>Si « Accident de la route » coché</u> :

- **Etiez-vous :**
  - ❐ Piéton
  - ❐ En voiture Précisez : ❐ Conducteur ❐ Passager
  - ❐ En 2 roues motorisé Précisez : ❐ Conducteur ❐ Passager
  - ❐ En vélo Précisez : ❐ Conducteur ❐ Passager

Mes consultations

q46. <u>Au cours des 12 derniers mois</u>, avez-vous consulté un :

	Non	Oui	<u>Si oui</u>, combien de fois ?
**Médecin généraliste**	❐	❐	|__|__|
**Dentiste**	❐	❐	|__|__|
**Ophtalmologue**	❐	❐	|__|__|

<u>(Pour les filles uniquement)</u>

q47. <u>Au cours des 12 derniers mois</u>, avez-vous consulté un :

	Non	Oui	<u>Si oui</u>, combien de fois ?
**Gynécologue**	❐	❐	|__|__|

q48. **<u>Au cours des 12 derniers mois</u>, avez-vous consulté un autre spécialiste:**

❒ Oui ❒ Non

►**<u>Si oui,</u> précisez dans le tableau suivant :**

	Non	Oui	<u>Si oui</u>, combien de fois ?
**Dermatologue**	❒	❒	|__|__|
**Neurologue**	❒	❒	|__|__|
**Urologue**	❒	❒	|__|__|
**Gastroentérologue**	❒	❒	|__|__|
**Otorhinolaryngologiste (ORL)**	❒	❒	|__|__|
**Autre spécialiste**<br>**Précisez : ………………..**	❒	❒	|__|__|

q49. **<u>Au cours des 12 derniers mois</u>, avez-vous consulté un psychiatre, un psychologue ou psychothérapeute ?**

❒ Oui ❒ Non ❑ Ne souhaite pas répondre

►**<u>Si oui,</u> précisez le nombre total de fois :** |__|__|__|

q50. **<u>Si vous n'avez pas été consulter un dentiste au cours des 12 derniers mois</u>, quelle en était la(les) raison(s) ?**

- ❒ je ne pensais pas en avoir besoin (pas de problèmes dentaires)
- ❒ je n'en avais pas les moyens financiers
- ❒ mon emploi du temps ne le permettait pas
- ❒ j'appréhendais d'aller voir le dentiste
- ❒ je ne connais pas de dentiste
- ❒ autres raisons
- Précisez : ……………………………………………..

<u>(Pour les filles uniquement)</u>

q51. **<u>Si vous n'avez pas été consulter de gynécologue au cours des 12 derniers mois</u>, quelle en était la(les) raison(s) ?**

- ❒ je suis suivie par votre médecin généraliste
- ❒ je vais au planning familial ou au centre de planification
- ❒ je n'en avais pas les moyens financiers
- ❒ mon emploi du temps ne le permettait pas
- ❒ j'appréhendais d'aller voir le gynécologue
- ❒ je n'ai pas pensé que cela puisse être utile
- ❒ je ne connais pas de gynécologue
- ❒ je n'ai pas pu avoir de RDV
- ❒ autres raisons

- Précisez : ………………………………………..

q52. **<u>Au cours des 12 derniers mois</u>, malgré le besoin d'aller consulter un médecin (médecin généraliste, médecin spécialiste, ophtalmologue, ….) vous avez renoncé à y aller ?**

❒ Oui ❒ Non

►**<u>Si oui</u> précisez la(les) raison(s) :**

- ❒ délais d'attente trop longs
- ❒ je n'en avais pas les moyens financiers
- ❒ mon emploi du temps ne le permettait pas
- ❒ j'appréhendais d'aller voir le médecin, de faire des examens ou de me soigner
- ❒ j'ai préféré attendre que les choses aillent mieux d'elles-mêmes
- ❒ je ne connais pas de médecin
- ❒ je suis allé(e) aux urgences
- ❒ autres raisons
- Précisez : ………………………………………..

q53. **<u>Au cours des 12 derniers mois</u>, malgré le besoin d'aller consulter un dentiste, vous avez renoncé à y aller ?**

❒ Oui ❒ Non

►**<u>Si oui</u> précisez la(les) raison(s) :**

- ❒ délais d'attente trop longs
- ❒ je n'en avais pas les moyens financiers
- ❒ mon emploi du temps ne le permettait pas
- ❒ j'appréhendais d'aller voir le dentiste, de faire des examens ou de me soigner
- ❒ j'ai préféré attendre que les choses aillent mieux d'elles-mêmes
- ❒ je ne connais pas de dentiste
- ❒ je suis allé(e) aux urgences
- ❒ autres raisons
- Précisez : ………………………………………..

q54. **<u>Au cours des 12 derniers mois</u>, vous est-il arrivé de ne pas prendre un traitement en pharmacie prescrit par votre médecin (en partie ou en totalité)**

❒ Oui ❒ Non

- ►**<u>Si oui</u> précisez la(les) raison(s) :**
- ❒ je n'ai pas pris le temps
- ❒ je n'en avais pas les moyens financiers
  - o ❒ pour faire l'avance
  - o ❒ pour les traitements non remboursés
- ❒ mon emploi du temps ne le permettait pas
- ❒ je les avais déjà

- ❒ j'appréhendais de prendre le traitement
- ❒ j'ai préféré attendre que les choses aillent mieux d'elles-mêmes
- ❒ je n'ai pas pensé que cela puisse être utile
- ❒ autres raisons - Précisez : ………………………………………..

q55. **<u>Au cours des 12 derniers mois</u>, vous est-il arrivé de ne pas aller réaliser des examens complémentaires prescrits par un médecin (prise de sang, radio, …) ?**

❒ Oui ❒ Non

- ►**<u>Si oui</u> précisez la(les) raison(s) :**

- ❒ je n'en avais pas les moyens financiers pour faire l'avance
- ❒ je ne savais pas combien cela allait me coûter (peur que cela soit trop cher)
- ❒ mon emploi du temps ne le permettait pas
- ❒ j'appréhendais d'effectuer les examens
- ❒ j'ai préféré attendre que les choses aillent mieux d'elles-mêmes
- ❒ je n'ai pas pensé que cela puisse être utile
- ❒ autres raisons
- Précisez : ………………………………………..

Ma vue

q56. **Avez-vous besoin de porter des lunettes ou lentilles de contact ?**

❒ Oui ❒ Non

q57. **<u>Si oui,</u> au cours des 12 derniers mois, vous est-il arrivé de renoncer à aller acheter de nouvelles lunettes pour raisons financières ?**

❒ Oui ❒ Non

Mesures anthropométriques

q58. **Quels sont:**

Votre poids à la dernière mesure:

|__|__|__|kg

❑ Ne sais pas

Votre taille :

|__|__|__|cm

❑ Ne sais pas

q59. Indiquez la silhouette à laquelle vous considérez ressembler le plus actuellement ?

► ***Pour les femmes***

1 2 3 4 5 6 7 8 9

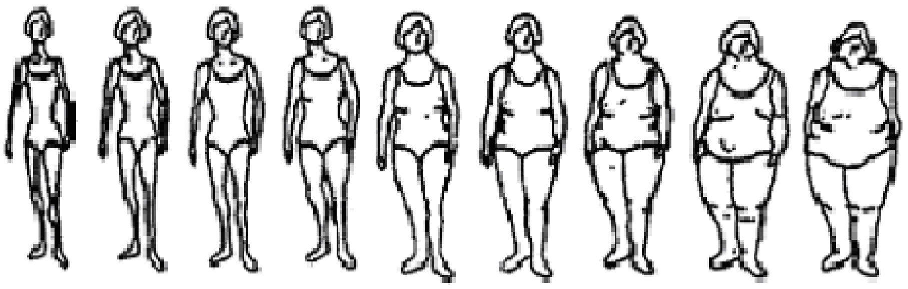

❑ ❑ ❑ ❑ ❑ ❑ ❑ ❑

► **Pour les hommes**

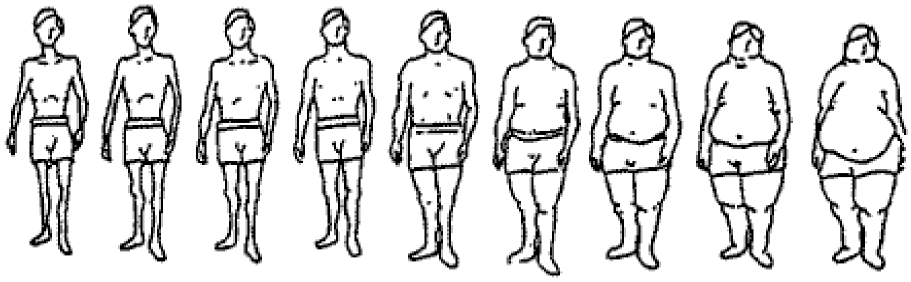

1 2 3 4 5 6 7 8 9

❑ ❑ ❑ ❑ ❑ ❑ ❑ ❑ ❑

q60. Indiquez à quelle silhouette vous souhaiteriez ressembler ?

(Avec les mêmes silhouettes)

La santé de ma famille

(Pour ceux ayant au moins un frère ou sœur)

q61. **L'un (ou plusieurs) de vos frères et sœurs sont peut-être décédés ?**

❒ Oui ❒ Non ❑ Ne souhaite pas répondre

►**Si oui,** merci de préciser la raison de son décès :

❒ Accident ❒ Cancer ❒ Autre Maladie ❒ Autre ❒ Suicide

❒ Ne sais pas ❑ Ne souhaite pas répondre

(Conditionner la question en fonction du nombre de frères et sœurs déclarés au début du questionnaire)

q62. **Votre père est-il en vie :**

❑ Oui

►**Si oui**, pourriez-vous préciser son âge : |__|__| ans ❒ Ne sais pas

❑ Non

►**Si non**, pourriez-vous préciser la raison de son décès :

❒ Accident ❒ Cancer ❒ Autre Maladie ❒ Autre ❒ Suicide

❒ Ne sais pas ❑ Ne souhaite pas répondre

►**Si non**, pourriez-vous préciser son âge au moment de son décès : |__|__| ans

❑ Ne sais pas

❑ Ne souhaite pas répondre

q63. **Votre mère est-elle en vie :**

❑ Oui

►**Si oui**, pourriez-vous préciser son âge : |__|__| ans ❒ Ne sais pas

❑ Non

►**Si non**, pourriez-vous préciser la raison de son décès :

❒ Accident ❒ Cancer ❒ Autre Maladie ❒ Autre ❒ Suicide

❒ Ne sais pas ❑ Ne souhaite pas répondre

►**Si non**, pourriez-vous préciser son âge au moment de son décès : |__|__| ans

❑ Ne sais pas

❑ Ne souhaite pas répondre

q64. A votre connaissance, vos parents ont-ils eu ou ont-ils actuellement

	Père	Mère
**Une maladie cardiovasculaire (infarctus du myocarde, angine de poitrine …)**	❑ Oui ❑ Non<br>❑ Ne sais pas	❑ Oui ❑ Non<br>❑ Ne sais pas
**Un accident vasculaire cérébral (AVC)**	❑ Oui ❑ Non<br>❑ Ne sais pas	❑ Oui ❑ Non<br>❑ Ne sais pas
**Un Cancer**	❑ Oui ❑ Non<br>❑ Ne sais pas	❑ Oui ❑ Non<br>❑ Ne sais pas
**Des problèmes de dépression, d'anxiété**	❑ Oui ❑ Non<br>❑ Ne sais pas	❑ Oui ❑ Non<br>❑ Ne sais pas
**Des maux de tête sévères, régulièrement**	❑ Oui ❑ Non<br>❑ Ne sais pas	❑ Oui ❑ Non<br>❑ Ne sais pas
**Des problèmes d'alcool**	❑ Oui ❑ Non<br>❑ Ne sais pas	❑ Oui ❑ Non<br>❑ Ne sais pas

Ou

❑ Ne souhaite pas répondre (à l'ensemble des items)

Comment je me sens ?

Mes activités physiques, culturelles

q65. En moyenne, combien de temps marchez-vous chaque jour ?

|__|__| minutes **Ou** |__|__| heures

q66. Vous arrive-t-il de vous déplacer en vélo ?

❑ Oui ❑ Non

►**Si oui**, merci de préciser si c'est :

❑ Quotidiennement (pour me rendre à mes cours…)
❑ De temps en temps (pour sortir le soir, ou le week-end)
❑ Occasionnellement (panne de transports en commun, …)

q67. Pratiquez-vous une ou plusieurs activités sportives régulières?

❑ Oui ❑ Non

►**Si oui, combien de fois ?**

❑ Une fois par mois
❑ 2 à 3 fois par mois
❑ Une fois par semaine
❑ Plus d'une fois par semaine

►**Si oui, quelle est la durée moyenne de votre activité lorsque vous la pratiquez ?**

❑ Moins d'1/2 h
❑ Entre 1/2 h et 1 h
❑ 1 heure
❑ Entre 1 h et 2 h
❑ Plus de 2h

►**Si oui, quel type de sport ?**

❑ Individuel (tennis, athlétisme, natation etc..)
►**Si oui, en** ❑ Loisir ou en ❑ Compétition

❑ Collectif (football, rugby, handball etc..)
►**Si oui, en** ❑ Loisir ou en ❑ Compétition

❑ A sensations (sports de glisse, escalade, parachutisme etc...)
►**Si oui, en** ❑ Loisir ou en ❑ Compétition

q68. Par rapport à vos années lycée, diriez-vous que vous avez une activité physique et sportive

❑ Moins importante ❑ Plus importante ❑ Equivalente

►**Si moins importante**, parce que :

- ❑ mes études représentent un frein (manque de temps)
- ❑ il n'y a pas d'équipements près de mon domicile ou du lieu de mes études
- ❑ les horaires d'ouverture ne sont pas compatibles avec mon emploi du temps
- ❑ problème de financement
- ❑ pas envie (d'y aller seul)
- ❑ mes autres activités extra universitaires me prennent trop de temps

q69. Souhaiteriez-vous pratiquer plus de sport:

❑ Oui ❑ Non

q70. En dehors de vos cours, avez-vous des activités extra universitaires régulières ?

❑ Oui ❑ Non

►**Si oui,** merci de préciser la ou lesquelles :

- ❑ Investissement en associations (en lien avec les études ou non)
- ❑ Pratique de la musique
- ❑ Pratique du théâtre
- ❑ Sorties au cinéma, théâtre, concerts
- ❑ Autres

Mon bien-être moral

q71. Veuillez cocher le degré d’accord avec les items suivants …

	Pas du tout d’accord	Plutôt pas d’accord	Ni d’accord, ni pas d’accord	Plutôt d’accord	Tout à fait d’accord
En général, ma vie correspond de près à mes idéaux	❑	❑	❑	❑	
Mes conditions de vie sont excellentes	❑	❑	❑	❑	
Je suis satisfait(e) de ma vie	❑	❑	❑	❑	
Jusqu'à maintenant, j'ai obtenu les choses importantes que je voulais de la vie	❑	❑	❑	❑	
Si je pouvais recommencer ma vie, je n'y changerais presque rien	❑	❑	❑	❑	

q72. <u>Au cours du dernier mois</u>, diriez-vous que :

	Jamais	~~Presque jamais~~ Rarement	~~Parfois~~ Quelquefois	~~Assez souvent~~ Souvent	~~Souvent~~ Très souvent
Il vous a semblé difficile de maîtriser les choses importantes de votre vie	❑	❑	❑	❑	❑
Vous vous êtes senti(e) confiant(e) dans vos capacités à prendre en mains vos problèmes personnels	❑	❑	❑	❑	❑
Vous avez senti que les choses allaient comme vous le vouliez	❑	❑	❑	❑	❑
Vous avez trouvé que les difficultés s’accumulaient à tel point que vous ne pouviez les contrôler	❑	❑	❑	❑	❑

q73. Au cours des 6 derniers mois, à quelle fréquence vous est-il arrivé de

	Jamais	Rarement	Quelquefois	Souvent	Très souvent
Avoir du mal à finaliser un projet lorsque le plus stimulant est fait ?	❑	❑	❑	❑	❑
Avoir des difficultés à ordonner les choses lorsqu'une tâche demande de l'organisation ?	❑	❑	❑	❑	❑
Avoir des difficultés à vous rappeler vos rendez-vous ou vos obligations ?	❑	❑	❑	❑	❑
Eviter ou remettre à plus tard une tâche qui demande beaucoup de réflexion?	❑	❑	❑	❑	❑
Remuer ou tortiller les mains ou les pieds lorsque vous devez rester longtemps assis ?	❑	❑	❑	❑	❑
Vous sentir excessivement actif, comme si vous étiez monté sur un ressort ?	❑	❑	❑	❑	❑

q74. Au cours de votre vie, avez-vous déjà été confronté à un évènement grave tel que : accidents graves, agressions, mort inattendue d'un proche, catastrophes naturelles …

❒ Oui ❒ Non ❑ Ne souhaite pas répondre

q75. Si oui, cet évènement a-t-il suscité en vous une peur intense, un sentiment d'impuissance ou d'horreur ?

❒ Oui ❒ Non ❑ Ne souhaite pas répondre

q76. Au cours des 12 derniers mois, vous êtes-vous senti triste, vide, sans énergie ou sans intérêt pour les choses pendant plusieurs jours de suite ?

❒ Oui ❒ Non ❑ Ne souhaite pas répondre

q77. <u>Au cours des 12 derniers mois</u>, vous est-il arrivé de penser à vous suicider (d'avoir des idées suicidaires) ?

❒ Non jamais
❒ Oui ça m'est déjà arrivé
❒ Oui à de multiples reprises
<u>Si coché :</u>
En avez-vous parlé à vos proches (amis ou famille) ?
❒ oui ❒ non
En avez-vous parlé avec un médecin ou psychologue ?
❒ oui ❒ non
Avez-vous été pris(e) en charge (suivi régulier, prise de traitements …)
❒ oui ❒ non
❑ Ne souhaite pas répondre

q78. <u>Au cours de votre vie</u>, avez-vous déjà fait une tentative de suicide ?

❒ Oui ❒ Non ❑ Ne souhaite pas répondre

Mon sommeil :

q79. <u>Au cours des 3 derniers mois</u>, comment avez-vous dormi ?

❒ Bien
❒ Plutôt bien
❒ Ni bien ni mal
❒ Plutôt mal
❒ Mal

q80. <u>Au cours des 3 derniers mois</u>, avez-vous eu des difficultés d'endormissement et/ou de maintien de votre sommeil (réveils nocturnes) ?

❒ Jamais ou moins d'1 fois par mois
❒ Moins d'1 fois par semaine
❒ 1 à 2 jours par semaine
❒ 3 à 5 jours par semaine
❒ Tous les jours ou presque

q81. <u>Au cours des 3 derniers mois</u>, vous êtes-vous senti extrêmement somnolent durant la journée ?

❒ Jamais ou moins d'1 fois par mois
❒ Moins d'1 fois par semaine
❒ 1 à 2 jours par semaine
❒ 3 à 5 jours par semaine
❒ Tous les jours ou presque

q82. **<u>Habituellement</u>, estimez-vous manquer de sommeil (au moins 1 heure de moins que vos besoins) ?**

❐ Jamais
❐ Quelques fois par an
❐ Quelques fois par mois
❐ Plusieurs fois par semaine
❐ Toujours

Ma vie sexuelle

q83. **Avez-vous déjà eu un rapport sexuel ?**

❑ Oui ❑ Non ❑ Ne souhaite pas répondre

► *(Si non ou ne souhaite pas répondre, question suivante = q89 pour les filles et q94 pour les hommes))*

► **Si oui, :**

q84. **A quel âge avez-vous eu votre premier rapport sexuel ?**

|__|__| ans ou ❑ Ne souhaite pas répondre

q85. **<u>Au cours des 12 derniers mois</u>, combien de partenaires homme avez-vous eu ?**

|__|__| homme(s) ou ❑ Ne souhaite pas répondre

q86. **<u>Au cours des 12 derniers mois</u>, combien de partenaires femme avez-vous eu ?**

|__|__| femme(s) ou ❑ Ne souhaite pas répondre

q87. **Lors de ces rapports, est-ce que des préservatifs ont été utilisés ?**

❑ Oui, à chaque fois
❑ Non pas avec tous ou pas tout le temps
❑ Non jamais
❑ Ne souhaite pas répondre

q88. **<u>Au cours des 12 derniers mois</u>, un médecin a-t-il déjà diagnostiqué chez vous une infection sexuellement transmissible (chlamydia, gonocoques, condylome, syphilis, herpes) ?**

❒ Oui ❒ Non ❑ Ne souhaite pas répondre

<u>(Pour les filles uniquement)</u>

q89. **A quel âge environ avez-vous eu vos premières règles ?**

|__|__| ans

Ou

❑ Je n'ai jamais eu de règles

q90. **Utilisez-vous actuellement un contraceptif oral (pilule)?**

❑ Oui ❑ Non

►Si oui, précisez lequel : ....*<u>(Liste des contraceptifs oraux)</u>*..........

q91. **Utilisez-vous un autre moyen de contraception ?**

❑ Oui ❑ Non

►Si oui, précisez :
❑ Implant
❑ Préservatif
❑ Autre

q92. **Avez-vous déjà utilisé une contraception d'urgence (pilule du lendemain type Norvelo®, Levonorgestrel® ou Ellaone®) ?**

❑ Oui ❑ Non ❑ Ne souhaite pas répondre

► **<u>Si oui</u>, combien de fois cela s'est-il produit ?**

❑ 1 fois ❑ 2, 3 fois ❑ Plus de 3 fois ❑ Ne souhaite pas répondre

q93. Avez-vous déjà eu recours à une IVG ?

❑ Oui ❑ Non

❑ Ne souhaite pas répondre

Qu'est-ce que je consomme ?

Mes habitudes alimentaires

q94. Actuellement, suivez-vous un régime alimentaire particulier ?

❑ Oui ❑ Non

► **Si oui, merci de préciser**

❑ Pour raisons médicales
❑ Pour perdre ou ne pas prendre de poids
❑ Pour rester en forme
❑ Je suis végétarien ou végétalien
❑ Autre

q95. Habituellement, mangez-vous du pain, des biscottes ou des céréales du type « petit déjeuner » chaque jour ?

« Sous forme de bulle informative » :
Ce groupe comprend le pain, les biscottes sous toutes leurs formes et les céréales de petit déjeuner.

❑ Oui ❑ Non

► Si oui combien de fois en mangez-vous par jour ?
❑ 1 fois
❑ 2 fois
❑ 3 fois
❑ 4 fois et plus

► Si non :
combien de fois en mangez-vous ?
❑ 4 à 6 fois par semaine
❑ 2 à 3 fois par semaine
❑ Une fois par semaine ou moins
❑ Jamais

q96. Habituellement, mangez-vous du riz, des pâtes, des pommes de terre, de la semoule ou du maïs chaque jour ?

Ce groupe comprend aussi le blé, la purée lyophilisée, les raviolis, lasagnes, hachis-Parmentier, gratins dauphinois, etc. et tous les plats cuisinés à base de riz, pâtes, pomme de terre ou semoule.

❑ Oui ❑ Non

► Si oui combien de fois en mangez-vous par jour ?
❑ 1 fois
❑ 2 fois
❑ 3 fois
❑ 4 fois et plus

► Si non :
combien de fois en mangez-vous ?
❑ 4 à 6 fois par semaine
❑ 2 à 3 fois par semaine
❑ Une fois par semaine ou moins
❑ Jamais

q97. <u>Habituellement</u>, mangez-vous des produits laitiers <u>chaque jour</u> ?

Cette section comprend le lait (aromatisé ou nature), les fromages, les yaourts (nature ou aux fruits), le fromage blanc et les petits suisses.
Les desserts lactés tels que les crèmes dessert ou les flans, ainsi que les yaourts à boire, sont compris dans les produits sucrés à cause de leur teneur en sucre et en matière grasse et ne sont pas inclus dans cette question.

❑ Oui ❑ Non

► <u>Si oui</u> combien de fois en mangez-vous par jour ?
❑ 1 fois
❑ 2 fois
❑ 3 fois
❑ 4 fois et plus

► <u>Si non</u> :
combien de fois en mangez-vous ?
❑ 4 à 6 fois par semaine
❑ 2 à 3 fois par semaine
❑ Une fois par semaine ou moins
❑ Jamais

q98. <u>Habituellement</u>, consommez-vous des fruits (y compris des jus de fruits 100% pur jus) <u>chaque jour</u> ?

Cette section comprend les fruits sous toutes leurs formes (crus ou cuits, en compote, en conserve, surgelés, au sirop, soupes, etc.). Les jus de fruits 100% pur jus ou sans sucre ajouté, les tartes et les gâteaux à base de fruits sont compris dans cette catégorie. Ne sont pas compris les nectars, « boissons à base fruits », etc.

❑ Oui ❑ Non

► <u>Si oui</u> combien de fois en mangez-vous par jour ?
❑ 1 fois
❑ 2 fois
❑ 3 fois
❑ 4 fois et plus

► <u>Si non</u> :
combien de fois en mangez-vous ?
❑ 4 à 6 fois par semaine
❑ 2 à 3 fois par semaine
❑ Une fois par semaine ou moins
❑ Jamais

q99. <u>Habituellement</u>, mangez-vous des légumes (sans compter les pommes de terre et les légumes secs) <u>chaque jour</u> ?

Cette section comprend les légumes sous toutes leurs formes (purée, en conserve, surgelés, soupes, tartes, etc.), qu'ils soient crus ou cuits. La sauce tomate fait partie de cette catégorie, mais pas les pommes de terre.

❑ Oui ❑ Non

► <u>Si oui</u> combien de fois en mangez-vous par jour ?
❑ 1 fois
❑ 2 fois
❑ 3 fois
❑ 4 fois et plus

► <u>Si non</u> :
combien de fois en mangez-vous ?
❑ 4 à 6 fois par semaine
❑ 2 à 3 fois par semaine
❑ Une fois par semaine ou moins
❑ Jamais

q100. **<u>Habituellement</u>, mangez-vous de la viande, de la volaille, du jambon ou des œufs <u>chaque jour</u> ?**

Cette catégorie comprend toutes les viandes, les oeufs sous toutes leurs formes, les abats, etc. Pour le jambon, cette question ne concerne que le jambon blanc (jambon cuit). Le reste de la charcuterie (le jambon cru, les saucisses, rillettes, pâtés, saucisson etc.) n'est pas compris dans cette catégorie.

❑ Oui ❑ Non

► <u>Si oui</u> combien de fois en mangez-vous par jour ?
❑ 1 fois
❑ 2 fois
❑ 3 fois
❑ 4 fois et plus

► <u>Si non</u> :
combien de fois en mangez-vous ?
❑ 4 à 6 fois par semaine
❑ 2 à 3 fois par semaine
❑ Une fois par semaine ou moins
❑ Jamais

q101. **<u>Habituellement</u>, mangez-vous du poisson ou d'autres produits de la pêche <u>chaque jour</u> ?**

Cette catégorie comprend le poisson sous toutes ses formes (dont le poisson en conserve, le poisson pané) et les fruits de mer.
N'oubliez pas toutes les préparations à base de fruits de mer dans cette catégorie (tartes aux fruits de mer, bouchées à la reine aux fruits de mers, etc.)

❑ Oui ❑ Non

► <u>Si oui</u> combien de fois en mangez-vous par jour ?
❑ 1 fois
❑ 2 fois
❑ 3 fois
❑ 4 fois et plus

► <u>Si non</u> :
combien de fois en mangez-vous ?
❑ 4 à 6 fois par semaine
❑ 2 à 3 fois par semaine
❑ Une fois par semaine ou moins
❑ Jamais

q102. **<u>Habituellement</u>, mangez-vous des légumes secs (haricots secs, lentilles, pois chiches etc.) <u>chaque jour</u> ?**

Ce groupe comprend tous les légumes secs (pois chiches, pois cassés, flageolets, maïs, lentilles, haricots blancs, rouges, fèves) et les préparations à base de légumes secs (saucisses lentilles, couscous si il y a des pois chiches, etc.).

❑ Oui ❑ Non

► <u>Si oui</u> combien de fois en mangez-vous par jour ?
❑ 1 fois
❑ 2 fois
❑ 3 fois
❑ 4 fois et plus

► <u>Si non</u> :
combien de fois en mangez-vous ?
❑ 4 à 6 fois par semaine
❑ 2 à 3 fois par semaine
❑ Une fois par semaine ou moins
❑ Jamais

q103. **<u>Habituellement</u>, buvez-vous des boissons sucrées <u>chaque jour</u> ?**

Ce groupe comprend les sodas, les sirops, les nectars…
Les purs jus de fruits ne font pas partie de cette catégorie.

❑ Oui ❑ Non

► <u>Si oui</u> combien de fois en buvez-vous par jour ?
❑ 1 fois
❑ 2 fois
❑ 3 fois
❑ 4 fois et plus

► <u>Si non</u> :
combien de fois en buvez-vous ?
❑ 4 à 6 fois par semaine
❑ 2 à 3 fois par semaine
❑ Une fois par semaine ou moins
❑ Jamais

q104. **<u>Habituellement</u>, combien de fois mangez-vous des burgers, kebab, pizzas…<u>chaque jour</u> ?:**

❑ Oui ❑ Non

► <u>Si oui</u> combien de fois en mangez-vous par jour ?
❑ 1 fois
❑ 2 fois
❑ 3 fois
❑ 4 fois et plus

► <u>Si non</u> :
combien de fois en mangez-vous ?
❑ 4 à 6 fois par semaine
❑ 2 à 3 fois par semaine
❑ Une fois par semaine ou moins
❑ Jamais

q105. Habituellement, vous arrive-t-il de consommer des sucreries, viennoiseries, barres chocolatées, gâteaux entre les repas chaque jour ?

❑ Oui ❑ Non

► Si oui combien de fois en mangez-vous par jour ?
❑ 1 fois
❑ 2 fois
❑ 3 fois
❑ 4 fois et plus

► Si non :
combien de fois en mangez-vous ?
❑ 4 à 6 fois par semaine
❑ 2 à 3 fois par semaine
❑ Une fois par semaine ou moins
❑ Jamais

q106. Habituellement, vous arrive-t-il de consommer des produits gras et salés, tels que des chips, biscuits apéritifs, saucisson… chaque jour ?

❑ Oui ❑ Non

► Si oui combien de fois en mangez-vous par jour ?
❑ 1 fois
❑ 2 fois
❑ 3 fois
❑ 4 fois et plus

► Si non :
combien de fois en mangez-vous ?
❑ 4 à 6 fois par semaine
❑ 2 à 3 fois par semaine
❑ Une fois par semaine ou moins
❑ Jamais

q107. Avez-vous le sentiment d'avoir globalement une alimentation saine et équilibrée ?

❑ Oui ❑ Non

►Si non :
❑ je n'ai pas les moyens financiers
❑ je n'ai pas facilement accès aux produits (supermarché, supérette …)
❑ je ne sais pas définir une telle alimentation
❑ je n'ai pas le temps d'en préparer
❑ cela ne me préoccupe pas du tout
❑ par choix (je n'arrive pas à me passer de certains produits)
❑ pour une autre raison

q108. Vous êtes-vous déjà fait(e) vomir parce que vous ne vous sentiez pas bien "l'estomac plein"?

❑ Oui ❑ Non

q109. Craignez-vous d'avoir perdu le contrôle des quantités que vous mangez?

❑ Oui ❑ Non

q110. **Avez-vous récemment perdu plus de six kilogrammes en moins de trois mois?**

❑ Oui ❑ Non

q111. **Pensez-vous que vous êtes trop gros(se) alors que les autres vous trouvent trop mince?**

❑ Oui ❑ Non

q112. **Diriez-vous que la nourriture est quelque chose qui occupe une place dominante dans votre vie?**

❑ Oui ❑ Non

Ma consommation de tabac

q113. **Est-ce que vous fumez du tabac (cigarettes et/ou tabac à rouler) régulièrement ou occasionnellement ?**

❑ Oui ❑ Oui, mais en train d'arrêter ❑ Non

► **Si non**
Avez-vous déjà fumé ?

- ❑ Non, je n'ai jamais fumé
- ❑ Oui j'ai déjà fumé occasionnellement
- ❑ Oui j'ai déjà fumé quotidiennement pendant au moins 6 mois

► **Si « Oui » et « Oui, mais en train d'arrêter »**

►**A quel âge avez-vous commencé à fumer ? :** |__|__| ans

►**Combien de cigarettes fumez-vous en moyenne ?**

|__|__| par jour

Ou |__|__| par semaine

Ou |__|__| par mois

► Pour les « Oui » uniquement

►Avez-vous envie d'arrêter de fumer ?

❑ Oui dans l'année ❑ Oui mais plus tard ❑ Non ❑ Ne sais pas

q114. Au cours des 12 derniers mois, avez-vous déjà consommé à plusieurs reprises :

De la chicha : ❑ Oui ❑ Non

Du tabac à chiquer ❑ Oui ❑ Non

Ma consommation d'alcool

q115. Combien de fois vous arrive-t-il de consommer de l'alcool (boissons alcoolisées telles que la bière le vin, les alcools type whisky, vodka, tequila.. même mélangés)?

❑ Jamais *(Si jamais, question suivante = q125)*
❑ 1 fois par an
❑ Plusieurs fois par an
❑ 1 fois par mois
❑ 1 fois par semaine ou moins
❑ 2 à 3 fois par semaine
❑ 4 à 6 fois par semaine
❑ Tous les jours

q116. Au cours d'une même occasion (soirée) où vous buvez, combien de fois vous arrive-t-il de boire au moins 6 verres ?

❑ Jamais
❑ 1 fois par an
❑ Plusieurs fois par an
❑ 1 fois par mois
❑ 1 fois par semaine ou moins
❑ 2 à 3 fois par semaine
❑ 4 à 6 fois par semaine
❑ Tous les jours

q117. Dans l'année écoulée, combien de fois avez-vous observé que vous n'étiez plus capable de vous arrêter de boire après avoir commencé ?

❑ Jamais
❑ 1 fois par an

❑ Plusieurs fois par an
❑ 1 fois par mois
❑ 1 fois par semaine ou moins
❑ 2 à 3 fois par semaine
❑ 4 à 6 fois par semaine
❑ Tous les jours

q118. <u>Dans l'année écoulée</u>, combien de fois, après une période de forte consommation, avez-vous du boire de l'alcool dès le matin pour vous remettre en forme ?

❑ Jamais
❑ 1 fois par an
❑ Plusieurs fois par an
❑ 1 fois par mois
❑ 1 fois par semaine ou moins
❑ 2 à 3 fois par semaine
❑ 4 à 6 fois par semaine
❑ Tous les jours

q119. <u>Dans l'année écoulée</u>, combien de fois avez-vous eu un sentiment de culpabilité ou de regret après avoir bu ?

❑ Jamais
❑ 1 fois par an
❑ Plusieurs fois par an
❑ 1 fois par mois
❑ 1 fois par semaine ou moins
❑ 2 à 3 fois par semaine
❑ 4 à 6 fois par semaine
❑ Tous les jours

q120. <u>Dans l'année écoulée</u>, combien de fois vous est-il arrivé d'être ivre (avoir bu au point d'être « complètement saoul ») ?

❑ Jamais
❑ 1 fois par an
❑ Plusieurs fois par an
❑ 1 fois par mois
❑ 1 fois par semaine ou moins
❑ 2 à 3 fois par semaine
❑ 4 à 6 fois par semaine
❑ Tous les jours

q121. **<u>Dans l'année écoulée</u>, combien de fois avez-vous été incapable de vous souvenir de ce qui s'était passé la nuit précédente parce que vous aviez bu ?**

❑ Jamais
❑ 1 fois par an
❑ Plusieurs fois par an
❑ 1 fois par mois
❑ 1 fois par semaine ou moins
❑ 2 à 3 fois par semaine
❑ 4 à 6 fois par semaine
❑ Tous les jours

q122. **Vous êtes-vous blessé ou avez-vous blessé quelqu'un parce que vous aviez bu ?**

❑ Oui ❑ Non ❑ Ne souhaite pas répondre

q123. **Avez-vous déjà ressenti le besoin d'abaisser votre consommation d'alcool ?**

❑ Oui ❑ Non

q124. **Est-ce qu'un membre de votre entourage, un médecin ou un autre professionnel de santé s'est déjà préoccupé de votre consommation d'alcool et vous a conseillé de diminuer ?**

❑ Oui ❑ Non

<u>Ma consommation de substances psycho actives</u>

q125. **<u>Au cours de votre vie</u>, avez-vous déjà consommé du cannabis (shit, joint, haschich, herbe…)**

❑ Oui ❑ Non ❑ Ne souhaite pas répondre

<u>(Si non ou ne souhaite pas répondre, question suivante = 127)</u>

► **<u>Si oui :</u>**

q126. **<u>Au cours des 12 derniers mois</u> avez-vous consommé du cannabis**

❑ Oui ❑ Non ❑ Ne souhaite pas répondre

► **<u>Si oui</u>**, pouvez-vous préciser le nombre de fois :

❑ Tous les jours ❑ Plusieurs fois par semaine
❑ Une fois par semaine ❑ Plusieurs fois par mois
❑ Une fois par mois ou moins ❑ Une seule fois
❑ Juste en soirées

q127. <u>Au cours de votre vie</u>, avez-vous pris un des produits suivants ?

	Non jamais	Une seule fois pour essayer	Plus d'une fois	Ne souhaite pas répondre
**Ecstasy, MD, MDMA**	❑	❑	❑	❑
**<u>Si « plus d'une fois »</u> :**				
**Au cours des 12 derniers mois, combien de fois en avez-vous pris ?**	1 fois ❑	Entre 1 et 10 fois ❑	Plus de 10 fois ❑	
**Amphétamines (Speed)**	❑	❑	❑	❑
**<u>Si « plus d'une fois »</u> : *<u>idem précédent</u>***				
**Protoxyde d'azote (gaz hilarant)**	❑	❑	❑	❑
**<u>Si « plus d'une fois »</u> : *<u>idem précédent</u>***				
**Produit à inhaler (type poppers)**	❑	❑	❑	❑
**<u>Si « plus d'une fois »</u> : *<u>idem précédent</u>***				
**Cocaïne**	❑	❑	❑	❑
**<u>Si « plus d'une fois »</u> : *<u>idem précédent</u>***				

Ou
❑ Ne souhaite pas répondre (à l'ensemble des items de ce tableau)

q128. <u>Au cours de votre vie</u>, avez-vous pris d'autres substances psychoactives (drogues) ?

❑ Oui ❑ Non ❑ Ne souhaite pas répondre

►**<u>Si oui</u>**, merci de préciser dans la liste suivante :

	Oui	Non	Ne souhaite pas répondre
Champignons hallucinogènes (ou autres plantes hallucinogènes)	❑	❑	❑
Crack, free-base	❑	❑	❑
Héroïne	❑	❑	❑
LSD			
Kétamine	❑	❑	❑
GHB	❑	❑	❑
Autres drogues (précisez) : ………………………	❑	❑	❑

Ou

❑ Ne souhaite pas répondre (à l'ensemble des items de ce tableau)

q129. Avez-vous eu l'impression de prendre des substances psychoactives sans le savoir (à votre insu, dans votre verre …) ?

❑ Oui ❑ Non ❑ Ne sais pas ❑ Ne souhaite pas répondre

La question suivante ne s'affiche pas si q125, 127 et 128 = non

q130. Si vous avez déjà consommé des substances psychoactives, depuis votre entrée à l'université, votre consommation a-t-elle été modifiée ?

❑ Non, elle n'a pas été modifiée
❑ Oui, elle a augmenté
❑ Oui, elle a diminué
❑ Oui, j'ai essayé de nouveaux produits

Ma consommation de médicaments

q131. Actuellement, prenez-vous des médicaments régulièrement (pour un ou plusieurs problèmes de santé) ?

❒ Oui ❒ Non

►Si oui, s'agit-il (plusieurs réponses possibles) :

❒ De médicaments sur prescription
❒ De médicaments non prescrits (en vente libre)
❒ De médicaments empruntés à mon entourage
❒ Autre

q132. Au cours de 3 derniers mois, vous est-il arrivé de prendre des médicaments ou autre produit :

a) contre la douleur ?

❒ Oui ❒ Non

►**Si oui,** merci de préciser

- La fréquence d'utilisation :
  - ❒ Au moins 1 fois/jour
  - ❒ Plusieurs fois/semaine
  - ❒ Plusieurs fois/mois
  - ❒ 1 fois/mois ou moins
- Le type de médicaments pris (plusieurs réponses possibles) :

- ❒ Des médicaments sur prescription
- ❒ Des médicaments non prescrits (en vente libre)
- ❒ Des médicaments empruntés à mon entourage
- ❒ Autre

b) contre l'anxiété, l'angoisse ou le stress (dans la journée) ?

❒ Oui ❒ Non

►**Si oui,** merci de préciser

- La fréquence d'utilisation :
  - ❒ Au moins 1 fois/jour
  - ❒ Plusieurs fois/semaine
  - ❒ Plusieurs fois/mois
  - ❒ 1 fois/mois ou moins
- Le type de médicaments pris (plusieurs réponses possibles) :
  - ❒ Des médicaments sur prescription
  - ❒ Des médicaments non prescrits (en vente libre)
  - ❒ Des médicaments empruntés à mon entourage
  - ❒ Autre

c) pour dormir ?

❒ Oui ❒ Non

►**Si oui,** merci de préciser

- La fréquence d'utilisation :
  - ❒ Au moins 1 fois/jour
  - ❒ Plusieurs fois/semaine
  - ❒ Plusieurs fois/mois
  - ❒ 1 fois/mois ou moins
- Le type de médicaments pris (plusieurs réponses possibles) :
  - ❒ Des médicaments sur prescription
  - ❒ Des médicaments non prescrits (en vente libre)
  - ❒ Des médicaments empruntés à mon entourage
  - ❒ Autre

q133. En période d'examens, vous arrive-t-il de prendre des produits dans le but de vous aider à vous concentrer ?

❒ Oui ❒ Non

►**Si oui**, à quelle fréquence

❒ Régulièrement
❒ De temps en temps
❒ Occasionnellement

►**Si oui**, quel type de produit préférentiellement :

❒ Médicaments (prescrits, en achat libre, empruntés à votre entourage…)
❒ Homéopathie, Phytothérapie…
❒ Boissons énergisantes (contenant de la taurine, de la caféine…)
❒ Autres produits - Précisez : …………………………………..

q134. Pour la pratique d'activités sportives, vous arrive-t-il de prendre des produits dans le but d'être performant ?

❒ Oui ❒ Non

►**Si oui**, à quelle fréquence

❒ Régulièrement
❒ De temps en temps
❒ Occasionnellement

►**Si oui**, quel type de produit préférentiellement :

❒ Médicaments (prescrits, en achat libre, empruntés à votre entourage…)
❒ Homéopathie, Phytothérapie…
❒ Boissons énergisantes (contenant de la taurine, de la caféine…)
❒ Autres produits - Précisez : …………………………………..

q135. Au cours des 3 derniers mois, vous est-il arrivé de prendre des vitamines ou autres compléments alimentaires ?

❒ Oui ❒ Non

►**Si oui**, pouvez-vous préciser si vous avez pris:

- ❒ spécifiquement de la vitamine D
- ❒ spécifiquement de la vitamine E
- ❒ d'autres vitamines (A, C …)
- ❒ un mélange de vitamines
- ❒ des omégas 3
- ❒ autre
