# Supplementary material for: Prevalence, lived experiences and user profiles in e-cigarette use: A mixed methods study among French college students
Source: PLoS One. 2024 Feb 9;19(2):e0297156. doi: 10.1371/journal.pone.0297156 (PMC10857705; doi:10.1371/journal.pone.0297156)
Supplement: S4 File — (PDF) [file pone.0297156.s005.pdf]

i-Share

Internet-based **S**tudents **H**e**A**lth **R**esearch **E**nterprise

Baseline Questionnaire-

About you

Your studies

q1. High school diploma obtained in

- ❒ Science
- ❒ Humanities
- ❒ Economics
- ❒ Professional
- ❒ Technology
- ❒ Other - Please specify

q2. Year high school diploma obtained: |__|__|__|__|

q3. You are currently enrolled at (university indicated during registration), please specify your field of study:

(This is displayed depending on the university indicated at the time of registration)

Here we provide some examples for Bordeaux

❒ University Bordeaux 1 Science and technology

- Field of study:
  ❒ Biology ❒ Chemistry ❒ IT ❒ Mathematics
  ❒ Applied mathematics ❒ Chemical Physics ❒ Physics and engineering ❒ Environmental science
  ❒ Natural science ❒ Other

❒ University Bordeaux Segalen

- Field of study:
  ❒ Health (❒ Medicine ❒ Pharmacy ❒ Odontology ❒ Human sciences ❒ Life sciences ❒ Science and modelling)
  ❒ Social science ❒ Psychology ❒ Sociology
  ❒ Other

❒ University Michel de Montaigne Bordeaux III

- Field of study:
  ❒ Urban planning ❒ Archaeology ❒ Arts
  ❒ Anthropology ❒ Art history
  ❒ Geography ❒ History ❒ Information and communications
  ❒ Languages and civilizations ❒ Foreign languages
  ❒ Literature ❒ Philosophy ❒ Linguistics
  ❒ Other

❒ University Montesquieu Bordeaux IV

- Field of study:
  ❒ Law ❒ Political science
  ❒ Economics ❒ Management ❒ Education
  ❒ Other

q4. Current year of study:

❒ 1st

❒ 2nd

❒ 3rd

❒ Other

Specify: ……………………………….

q5. Have you previously undertaken any studies in a different field?

❑ Yes ❑ No

►**If yes: what field:**

❒ Law and political science ❒ Economics ❒ Social Sciences

❒ Sciences ❒ Literature ❒ Health

❒ Other Specify: ……………………………….

q6. What is the highest level of study you wish to obtain?

❒ Bachelor

❒ Master

❒ Doctorate

❒ Other

Your family and background

q7. Do you have siblings?

❐ Yes ❐ No ❐ Do not wish to respond

► **If yes**:

Number of brothers: |__|__|

Number of sisters: |__|__|

q8. Are your parents divorced/separated?

❐ Yes ❐ No ❐ Do not wish to respond

► **If yes**, what was your age at the time of divorce/separation:

|__|__| years (*indicate "0" if this occurred before you were born or during your 1st year of life)*

q9. During your childhood and adolescence, where did you primarily live:

- ❐ With your parent(s) or adoptive parent(s)
- ❐ With your grandparents
- ❐ With another family member (uncle, aunt, etc.)
- ❐ Foster family
- ❐ Children's home
- ❐ Other
- ❐ Do not wish to respond

q10. During your childhood and adolescence, did you feel supported and comforted by your family?

- ❐ Not at all
- ❐ A little
- ❐ Moderately
- ❐ A lot
- ❐ Very much
- ❐ Do not wish to respond

q11. What is the highest level of education of the person(s) who raised you?

(You can complete only one column if)	**Adult 1**	**Adult 2**
**Don't know**	❐	❐
**Elementary School**	❐	❐
**High school**	❐	❐
**Higher education**	❐	❐
**Professional diploma**	❐	❐

q12. What was the economic situation of the family who raised you during childhood and adolescence?

- ❑ Very comfortable
- ❑ Comfortable
- ❑ Adequate
- ❑ Difficult
- ❑ Very difficult

q13. During your childhood/adolescence, did you have any pets?

❑ Yes ❑ No

►<u>If yes</u>, which animal(s):

- ❑ Cat
- ❑ Dog
- ❑ Mouse
- ❑ Other

Your current environment

q14. Where do you currently live?

❒ With parent(s)

❒ In student accommodation

❒ In an apartment

Specify:

❒ With a partner

❒ Alone

❒ With one or more roommate(s)

❒ Other Specify: ……………………….

q15. Do you have children?

❑ Yes ❑ No ❑ Do not wish to respond

q16. How often do you visit your parents during the academic year?

❒ Once a week

❒ Once a month

❒ During the holidays

❒ Once or twice a year

❒ Never

❒ Other - please specify: …………………….

q17. What are your sources of income during the academic year?

❒ Family

❒ Welfare/state support

❒ Scholarship

❒ Paid employment (internships, part time jobs, summer jobs)

❒ Other Specify: ………….. …………………….

(Only complete if you indicated that you rely on paid employment)

q18. What is the frequency of your employment?

❑ Full-time

❑ Part-time

❑ Regular part-time

❑ Occasional

❑ Only during holidays

❑ Paid internships

q19. Is this employment:

❑ Your main source of income

❑ Additional income to supplement essential living costs (e.g. housing, transport)

❑ Income to supplement leisure activities

❑ Other

q20. How do these activities affect your studies?

❑ Positively ❑ Negatively

❑ No impact

q21. Please rate how you feel about the following aspects of your life:

	Very satisfied	Somewhat satisfied	Satisfied	Somewhat dissatisfied	Very dissatisfied
Your economic resources	❑	❑	❑	❑	❑
Your housing	❑	❑	❑	❑	❑
Your leisure activities	❑	❑	❑	❑	❑
Your social life	❑	❑	❑	❑	❑
Your relationship with your parents	❑	❑	❑	❑	❑

q22. Do you have a driving license:

- **Car? (driving license)**
- ❒ Yes ❒ No
    - If yes in which year did you obtain your license?
    - |__|__|__|__|

- **Motorbike? (motorcycle license)**
- ❒ Yes ❒ No
    - If yes in which year did you obtain your license?
    - |__|__|__|__|

Technology

q23. Do you have a computer?

❐ Yes ❐ No

q24. Do you have a tablet?

❐ Yes ❐ No

q25. Do you have a smartphone?

❐ Yes ❐ No

q26. During the week, how much time do you spend on average doing the following:

**Per weekday**	**Never**	**Less than 30 minutes**	**30 mins to 2 hrs**	**2-4 hrs**	**4-8 hrs**	**More than 8 hrs**
• In front of a computer/tablet for work/study	❑	❑	❑	❑	❑	❑
• In front of a computer/tablet for pleasure/fun	❑	❑	❑	❑	❑	❑
• In front of a computer/tablet for Internet (social networking, messaging)	❑	❑	❑	❑	❑	❑
• Watching TV (or TV via a computer)	❑	❑	❑	❑	❑	❑
• Using a smartphone (internet, social networking, games)	❑	❑	❑	❑	❑	❑

Left or right-handed?

q27. For the following activities, please indicate the hand you prefer to use:

	Always left	Usually left	No preference left / right	Usually right	Always right
Writing	❑	❑	❑	❑	❑
Drawing	❑	❑	❑	❑	❑
Throwing a ball	❑	❑	❑	❑	❑
Cutting with scissors	❑	❑	❑	❑	❑
Brush teeth	❑	❑	❑	❑	❑
Cut with a knife	❑	❑	❑	❑	❑
Use a spoon	❑	❑	❑	❑	❑
Light a match	❑	❑	❑	❑	❑
Use a computer mouse	❑	❑	❑	❑	❑

q28. Indicate the handedness of your parents

	Left-handed	Right-handed	Forced right-handed*	Don't know
Father	❑	❑	❑	❑
Mother	❑	❑	❑	❑

**naturally left-handed, but has been forced to use the right hand to write.*

q29. How many do you have:

- Left-handed brothers (do not include half-brothers): |__|__| or ❑ Don't know
- Left-handed sisters (do not include half-sisters): |__|__| or ❑ Don't know

Question dependent on number of siblings stated

How are you?

Healthcare

q30. Are you registered with a student social security fund?

❐ Yes ❐ No ❐ Don't know

►**If yes, please specify:**

❑ For a fee (age > 20 years)
❑ For free (age < 20 years)

►***If no, why not?***

❑ Covered by work
❑ Have not yet registered
❑ Due to lack of information
❑ Other, Specify

q31. Do you have health insurance?

❐ Yes ❐ No ❐ Don't know

►**If yes, is it:**

❑ Student health insurance
❑ Health insurance via parents
❑ Private insurance
❑ Other

►**If no, why not?**

❑ For financial reasons
❑ Have not yet registered
❑ Due to lack of information
❑ Other - Please specify…………

Your health

q32. How do you rate your current health?

- ❑ Very good
- ❑ Good
- ❑ Average
- ❑ Poor
- ❑ Very poor

q33. Are you vaccinated against:

Hepatitis B ❒ Yes ❒ No ❒ Don't know

Measles, mumps and rubella: ❒ Yes ❒ No ❒ Don't know

(Women only)

The Human Papillomavirus (HPV): ❒ Yes ❒ No ❒ Don't know

(Gardasil®, Ceravix®)

q34. Is your health record accessible?

❒ Yes ❒ No ❒ Don't know

q35. Has a doctor ever diagnosed you with:

	Yes	No
**Asthma**	❑	❑
**Allergies**	❑	❑
**Eczema**	❑	❑
**Multiple sclerosis**	❑	❑
**Type I diabetes**	❑	❑
**Migraine**	❑	❑
**Tinnitus**	❑	❑
**Colitis (Crohn's disease, ulcerative colitis ...)**	❑	❑
**Juvenile arthritis**	❑	❑

(For each cited pathology, if the answer is "Yes", the following three questions appear)

If yes

- What was your approximate age at first diagnosis?
- _____ years
- Have you received formal treatment for this illness (regular medical treatment, prescription of drugs or other)?

  ❑ Yes ❑ No

- Have you been hospitalized for this disease?

  ❑ Yes ❑ No

q36. Have you ever been diagnosed with:

	Yes	No
**A hyperactivity disorder during your childhood**	❑	❑
**Anxiety disorders, phobia**	❑	❑
**Anorexia or bulimia**	❑	❑
**Obsessive Compulsive Disorder (OCD)**	❑	❑
**Depression**	❑	❑

(For each cited pathology, If the answer is "Yes", the following three questions appear)

If yes

- What was your approximate age at first diagnosis?

  _____ years

- Have you received formal treatment for this illness (regular medical treatment, prescription of drugs or other)?

  ❑ Yes ❑ No

- Have you been hospitalized for this illness?

  ❑ Yes ❑ No

q37. Do you have one or more other illness(es) for which you are being treated?

❒ Yes ❒ No

►**If yes**:

**Please specify:**<br>***Multiple answers allowed***
…………………………………….

(For each cited disease, if the answer is "Yes" the following three questions appear)

If yes

- What was your approximate age at first diagnosis?

  ______ years

- Does this disease negatively affect your studies?

  ❑ Yes ❑ No

- Do the treatment, care and medical follow-up for this disease negatively affect your studies?

  ❑ Yes ❑ No

q38. Have you ever had headaches?

❒ Yes ❒ No ***(If no, following question = q42)***

►**If yes**:

q39. During the last 12 months, have you had headaches lasting several hours?

❒ Yes ❒ No ***(If no, following question = q42)***

►**If yes**

q40. During these attacks:

	Yes	No	Not sure
The headache limits your physical or mental activities	❒	❒	❒
Pain is worse on just one side	❒	❒	❒
Pain is pounding, pulsing or throbbing	❒	❒	❒
Pain is made worse by activities such as walking or climbing stairs	❒	❒	❒
You feel nauseated or sick to your stomach	❒	❒	❒
Light or sound bothers you (a lot more than when you do not have a headache)	❒	❒	❒

q41. Is the headache sometimes associated with visual disturbances or numbness in some parts of your body, typically at the beginning of a headache? Have you ever had these symptoms?

❒ Yes ❒ No

q42. Have you ever been diagnosed with dyslexia?

❒ Yes ❒ No

►**<u>If yes</u>:**

Have you ever attended speech therapy sessions?
❒ Yes ❒ No

Do you have special exam arrangements?
❒ Yes ❒ No

q43. Do you have a disability?

❒ Yes ❒ No

►**<u>If yes</u>:**

Specify:

❒ Visual impairment ❒ Hearing impairment

❒ Mental or physical disability ❒ Mobility disability

❒ Other handicap - Please specify: ……………………………..

Does it negatively affect your studies?
❒ Yes ❒ No

Do you have special exam arrangements?
❒ Yes ❒ No

Your recent health history

q44. During the last 12 months, have you suffered from any of the following on multiple occasions?

- Rhino-pharyngitis (cold) ❒ yes ❒ no
- Pharyngitis ❒ yes ❒ no
- Flu or influenza ❒ yes ❒ no
- Gastroenteritis ❒ yes ❒ no

q45. Have you been a patient at a hospital or clinic in the last 12 months?

❒ Yes ❒ No

►If yes, please specify in the following table:

(Lines will be added according to the number of events declared by the respondent)

Cause	Place	The total length of the event (for any type of service)
❒ Sports injury<br>❒ Road accident<br>❒ Accidental injury at home<br>❒ Leisure accident<br>❒ Violence<br>❒ Illness (infection, chronic disease, etc.)<br>❒ Other - please specify: …………………	❒ Emergencies<br>❒ Psychiatric Emergencies<br>❒ Medical center<br>❒ Surgical service<br>❒ Clinic	❒ Less than one day<br>❒ One day<br>❒ From one to three days<br>❒ From three days to a week<br>❒ More than a week

If the answer is « Car accident »:

- **Were you:**
  - ❒ Pedestrian
  - ❒ In a car Specify: ❒ Driver ❒ Passenger
  - ❒ On a motorcycle Specify: ❒ Driver ❒ Passenger
  - ❒ By bike Specify: ❒ Driver ❒ Passenger

Your consultations

q46. <u>During the last 12 months</u>, have you consulted a:

	No	Yes	<u>If yes</u>, how many times?
**General Practitioner**	❒	❒	|__|__|
**Dentist**	❒	❒	|__|__|
**Eye-specialist**	❒	❒	|__|__|

(Women only)

q47. <u>During the last 12 months</u>, have you consulted a:

	No	Yes	<u>If yes</u>, how many times?
**Gynecologist**	❒	❒	|__|__|

q48. <u>During the last 12 months</u>, have you consulted another specialist:

❒ Yes ❒ No

► **<u>If yes,</u> Specify in the following table:**

	No	Yes	<u>If yes</u>, how many times?
**Dermatologist**	❒	❒	|__|__|
**Neurologist**	❒	❒	|__|__|
**Urologist**	❒	❒	|__|__|
**Gastroenterologist**	❒	❒	|__|__|
**Otolaryngologist**	❒	❒	|__|__|
**Other specialist** **Specify: ………………..**	❒	❒	|__|__|

q49. <u>During the last 12 months</u>, have you consulted a psychiatrist, psychologist or psychotherapist?

❒ Yes ❒ No ❑ Do not wish to respond

► **<u>If yes,</u> please specify the number of consultations:** \|__\|__\|__\|

q50. <u>If you have not consulted a dentist during the last 12 months</u>, please specify the reason(s):

- ❒ Didn't think I needed to (no teeth problems)
- ❒ Financial reasons
- ❒ Too busy
- ❒ Was afraid of going to see a dentist
- ❒ Don't know any dentists
- ❒ Other reason(s)
- Specify: …………………………………………..

(Women only)

q51. If you have not consulted a gynecologist during the last 12 months, please specify the reason(s):

- ❒ My GP takes care of my gynecological health
- ❒ Go to a family planning center or other women's health service
- ❒ Financial reasons
- ❒ Too busy
- ❒ Afraid of going to see a gynecologist
- ❒ Didn't think it would be useful
- ❒ Don't know any gynecologists
- ❒ Couldn't get an appointment
- ❒ Other reason(s)
- Specify: …………………………………………..

q52. During the last 12 months, have you avoided seeking treatment (general practitioner, consultant, eye specialist, etc.) despite needing it?

❒ Yes ❒ No

►**If yes please specify the reason(s):**

- ❒ Waiting times too long
- ❒ Financial reasons
- ❒ Too busy
- ❒ Afraid of going to see a doctor, having an examination or seeking treatment
- ❒ Preferred to wait until things got better by themselves
- ❒ Don't know any doctors
- ❒ Went to an emergency service
- ❒ Other reason(s)
- Specify: …………………………………………..

q53. During the last 12 months, have you avoided seeking dental treatment, despite needing it?

❒ Yes ❒ No

►**If yes please specify the reason(s):**

- ❒ Waiting times too long
- ❒ Financial reasons
- ❒ Too busy
- ❒ Afraid of going to see a dentist, having an examination or seeking treatment
- ❒ Preferred to wait until things got better by themselves
- ❒ Don't know any dentists
- ❒ Went to an emergency service
- ❒ Other reason(s)
- Specify: …………………………………………..

q54. <u>During the last 12 months</u>, have you avoided visiting a pharmacy for drugs prescribed by your doctor?

❒ Yes ❒ No

- ►**<u>If yes</u> please specify the reason(s):**
- ❒ Didn't have time
- ❒ I did not have the financial means
  - ❒ to pay up front
  - ❒ for non-reimbursed medicines
- ❒ Too busy
- ❒ Already had some
- ❒ Afraid of taking the medication
- ❒ Preferred to wait until things got better by themselves
- ❒ Didn't think it would help
- ❒ Other reasons - please specify: ………………………………………..

q55. <u>During the last 12 months</u>, have you avoided complementary health exams prescribed by a doctor (blood sample, scans, etc.)?

❒ Yes ❒ No

- ►**<u>If yes</u> please specify the reason(s):**
- ❒ Insufficient funds to make payment
- ❒ Didn't know how much it could cost (afraid it was too expensive)
- ❒ Too busy
- ❒ Afraid of medical exams
- ❒ Preferred to wait until things got better by themselves
- ❒ Didn't think it would help
- ❒ Other reasons - please specify: ………………………………………..

<u>Your sight</u>

q56. Do you need to wear glasses or contact lenses?

❒ Yes ❒ No

q57. <u>If yes,</u> during the last 12 months, have you avoided buying new glasses for financial reasons?

❒ Yes ❒ No

Your measurements

q58. Which are:

Your weight:

|__|__|__| kg

❑ Don't know

Your height:

|__|__|__| cm

❑ Don't know

q59. Please indicate which body shape most closely represents you at the moment:

► ***Women***

1 2 3 4 5 6 7 8 9

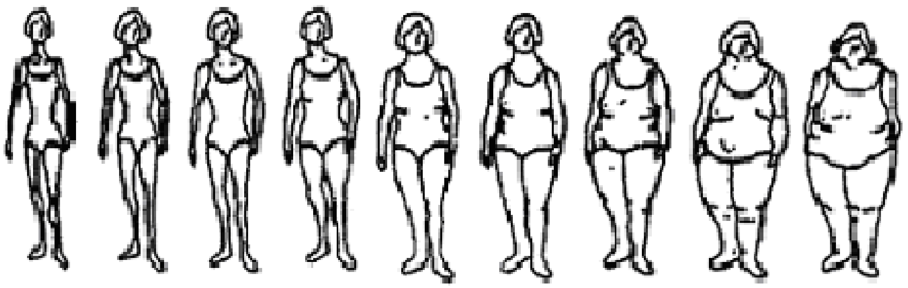

❑ ❑ ❑ ❑ ❑ ❑ ❑ ❑ ❑

► **Men**

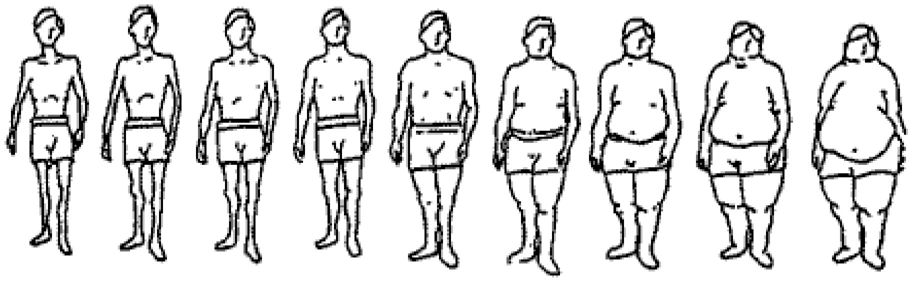

1 2 3 4 5 6 7 8 9

❑ ❑ ❑ ❑ ❑ ❑ ❑ ❑ ❑

q60. Please indicate which body shape you would most like to resemble:

(Using the same silhouettes)

Your family health

(For those with at least one brother or one sister)

q61. Are one or more of your brothers or sisters deceased?

❒ Yes ❒ No ❑ Do not wish to respond

►**If yes,** please specify the cause of death:

❒ Accident ❒ Cancer ❒ Other Disease ❒ Other ❒ Suicide

❒ Don't know ❑ Do not wish to respond

(This question is adapted according to the number of brothers and sisters stated at the start of the questionnaire)

q62. Is your father alive?

❑ Yes

►**If yes**, please specify his age: |__|__| years ❒ Don't know

❑ No

►**If no**, what was the cause of his death?

❒ Accident ❒ Cancer ❒ Other Disease ❒ Other ❒ Suicide

❒ Don't know ❑ Do not wish to respond

►**If no**, please specify his age at death: |__|__| years

❑ Don't know

❑ Do not wish to respond

q63. Is your mother alive?

❑ Yes

►**If yes**, please specify her age: |__|__| years ❒ Don't know

❑ No

►**If no**, what was the cause of her death?

❒ Accident ❒ Cancer ❒ Other Disease ❒ Other ❒ Suicide

❒ Don't know ❑ Do not wish to respond

►**If no**, please specify her age at death: |__|__| years

❑ Don't know

❑ Do not wish to respond

q64. To your knowledge, have your parents had or do they currently have:

	**Father**	**Mother**
**A cardiovascular disease (myocardial infarction, angina pectoris, etc.)**	❑ Yes ❑ No<br>❑ Don't know	❑ Yes ❑ No<br>❑ Don't know
**A stroke**	❑ Yes ❑ No<br>❑ Don't know	❑ Yes ❑ No<br>❑ Don't know
**A cancer**	❑ Yes ❑ No<br>❑ Don't know	❑ Yes ❑ No<br>❑ Don't know
**Depression or anxiety problems**	❑ Yes ❑ No<br>❑ Don't know	❑ Yes ❑ No<br>❑ Don't know
**Regular severe headaches**	❑ Yes ❑ No<br>❑ Don't know	❑ Yes ❑ No<br>❑ Don't know
**Alcohol problems**	❑ Yes ❑ No<br>❑ Don't know	❑ Yes ❑ No<br>❑ Don't know

Or

❑ Do not wish to respond (to all items)

How are you?

Your physical and cultural activities

q65. On average, how long do you walk each day?

|__|__| minutes **Or** |__|__| hours

q66. Do you ever cycle?

❑ Yes ❑ No

►**If yes**, please specify the frequency**:**

❑ Daily (to go to university, etc.)
❑ Sometimes (to go out at night, or during the weekend)
❑ Occasionally (when no public transport available)

q67. Do you regularly take part in one or more sports?

❑ Yes ❑ No

►**If yes, how often?**

❑ Once a month
❑ 2 to 3 times a month
❑ Once a week
❑ More than once a week

►**If yes, what is the average duration of the practice of your activity?**

❑ Less than half an hour
❑ Between half an hour and 1 hour
❑ 1 hour
❑ Between 1 and 2 hours
❑ More than 2 hours

►**If yes, which type of sport?**

❑ Individual (tennis, athletics, swimming, etc.)
►**If yes, for** ❑ Recreation or ❑ Competition

❑ Team (football, rugby, handball, etc.)
►**If yes, for** ❑ Recreation or ❑ Competition

❑ Extreme (snow sports, climbing, sky diving, etc.)
►**If yes, for** ❑ Recreation or ❑ Competition

q68. Compared to high school, is your physical and sport activity…

❑ Less frequent ❑ More frequent ❑ The same

►**<u>If less frequent,</u>** why?

- ❑ Too busy with my studies
- ❑ No sport facilities near my house or university
- ❑ Opening hours not compatible with my schedule
- ❑ Financial reasons
- ❑ Lack of motivation
- ❑ My other extracurricular activities take up too much time

q69. Would you like to practice more sport?

❑ Yes ❑ No

q70. Aside from your university courses, do you regularly take part in extracurricular activities?

❑ Yes ❑ No

►**<u>If yes</u>,** please specify:

- ❑ Involved in groups, organizations (related to your studies or otherwise)
- ❑ Music practice
- ❑ Theater practice
- ❑ Going to the cinema, theatre, concerts
- ❑ Other

Your mental health

q71. Please indicate how much you agree with the following:

	Strongly disagree	Slightly disagree	Neither agree nor disagree	Slightly agree	Strongly agree
In most ways my life is ideal	❑	❑	❑	❑	❑
My life is great	❑	❑	❑	❑	❑
I am satisfied with my life	❑	❑	❑	❑	❑
So far I've got all the important things I've wanted in life	❑	❑	❑	❑	❑
If I could start my life over, I would change almost nothing	❑	❑	❑	❑	❑

q72. In the last month:

	Never	Almost never	Sometimes	Fairly Often	Very often
You were unable to control the important things in your life	❑	❑	❑	❑	❑
You felt confident about your ability to handle your personal problems	❑	❑	❑	❑	❑
You felt that things were going your way	❑	❑	❑	❑	❑
You found that problems piled up so much that you couldn't overcome them	❑	❑	❑	❑	❑

q73. <u>During the last 6 months</u>, how often have you:

	Never	Rarely	Sometimes	Usually	Very usually
**Had difficulty finishing a project when the most interesting part is done?**	❑	❑	❑	❑	❑
**Had difficulty getting things in order when you have to do a task that requires organization?**	❑	❑	❑	❑	❑
**Had trouble remembering appointments or obligations?**	❑	❑	❑	❑	❑
**Avoided or put off a task which required a lot of thought?**	❑	❑	❑	❑	❑
**Fidgeted or twiddled your hands or feet when you needed to stay sitting for a long time?**	❑	❑	❑	❑	❑
**Felt excessively active, as if you were on a spring?**	❑	❑	❑	❑	❑

q74. Have you <u>ever</u> been exposed to a traumatic event such as a serious accident, violence, sudden death of a loved one, natural disaster, etc.

❒ Yes ❒ No ❑ Do not wish to respond

q75. <u>If yes</u>, has this event caused a sense of horror, helplessness or intense fear?

❒ Yes ❒ No ❑ Do not wish to respond

q76. <u>During the last 12 months</u>, have you felt sad, void, without energy or interest in things for several consecutive days?

❒ Yes ❒ No ❑ Do not wish to respond

q77. <u>During the last 12 months</u>, have you thought of killing yourself (have you had suicidal thoughts)?

❒ No never
❒ Yes, once

❒ Yes several times

If yes:

Have you talked about it with your family or friends?

❒ Yes ❒ No

Have you talked about it to a doctor or a psychologist?

❒ Yes ❒ No

Have you received formal treatment for this illness (regular medical treatment, prescription of drugs or other)?

❒ Yes ❒ No

❑ Do not wish to respond

q78. Have you ever attempted suicide?

❒ Yes ❒ No ❑ Do not wish to respond

Your sleep:

q79. During the last 3 months, how have you slept?

❒ Good
❒ Mostly good
❒ Neither good nor bad
❒ Mostly bad
❒ Bad

q80. During the last 3 months, have you had any difficulty falling asleep and/or staying sleep (waking up during the night)?

❒ Never or less than once a month
❒ Less than once a week
❒ 1 to 2 days a week
❒ 3 to 5 days a week
❒ Every day or almost every day

q81. During the last 3 months, have you felt extremely sleepy during the day?

❒ Never or less than once a month
❒ Less than once a week
❒ 1 to 2 days a week
❒ 3 to 5 days a week
❒ Every day or almost every day

q82. Do you think you normally lack sleep (at least 1 hour less than what you need)?

❒ Never

❒ Several times a year
❒ Several times a month
❒ Several times a week
❒ Always

Your sex life

q83. Have you ever had sexual intercourse?

❑ Yes ❑ No ❑ Do not wish to respond

► *(If "No" or "Do not wish to respond", following question = q89 for women and q94 for men)*

► **If yes:**

q84. How old were you when you first had sexual intercourse?

|__|__| years or ❑ Do not wish to respond

q85. During the last 12 months, how many men have you had sex with?

|__|__| man/men or ❑ Do not wish to respond

q86. During the last 12 months, how many women have you had sex with?

|__|__| woman/women or ❑ Do not wish to respond

q87. Did you use a condom on these occasions?

❑ Yes, every time
❑ No, not with all partners or not every time
❑ No never
❑ Do not wish to respond

q88. During the last 12 months, have you been diagnosed with a sexually transmitted infection (chlamydia, gonorrhea, warts, syphilis, herpes)?

❒ Yes ❒ No ❑ Do not wish to respond

(Women only)

q89. When did you get your first period?

|__|__| years
Or

❑ I haven’t had a period yet.

q90. Are you currently taking a birth control pill (oral contraceptive)?

❑ Yes ❑ No

►If yes, Specify which of the following: ……… ***(List of pills)*** ………..

q91. Do you use another method of contraception?

❑ Yes ❑ No

►If yes, please specify:

❑ Implant

❑ Condom

❑ Other

q92. Have you ever used emergency contraception (morning-after pill such as Norvelo®, Levonorgestrel® or Ellaone®) ?

❑ Yes ❑ No ❑ Do not wish to respond

► **If yes, how many times?**

❑ Once ❑ 2 - 3 times ❑ More than 3 times ❑ Do not wish to respond

q93. Have you ever had an abortion?

❑ Yes ❑ No ❑ Do not wish to respond

What do you eat?

Your eating habits

q94. Are you currently on a special diet?

❑ Yes ❑ No

► **If yes, please specify**

❑ For medical reasons
❑ To lose or maintain weight
❑ To be in shape
❑ I am vegetarian or vegan
❑ Other

q95. Do you usually eat bread, grains or cereals (such as cornflakes) every day?

Note: This group includes bread, all types of grains and breakfast cereals.

❑ Yes ❑ No

► If yes, how many times a day do you eat them?
❑ 1
❑ 2
❑ 3
❑ 4 or more

► If no, how many times do you eat them?
❑ 4 to 6 times a week
❑ 2 to 3 times a week
❑ Once a week or less
❑ Never

q96. Do you usually eat rice, pasta, potatoes, semolina or corn every day?

This group includes also wheat, dried mashed potatoes, ravioli, lasagna, etc. and all cooked dishes with rice, pasta, potatoes or semolina.

❑ Yes ❑ No

► If yes, how many times a day do you eat them?
❑ 1
❑ 2
❑ 3
❑ 4 or more

► If no, how many times do you eat them?
❑ 4 to 6 times a week
❑ 2 to 3 times a week
❑ Once a week or less
❑ Never

q97. Do you usually eat dairy products every day?

This group includes milk (flavored or plain), cheese, yogurts (fruit or plain) etc.
Dairy desserts such as creams, custards or yogurt drinks are included with sugary products given their sugar and fats composition and they are not included in this question.

❑ Yes ❑ No

► If yes, how many times a day do you eat them?
❑ 1
❑ 2
❑ 3
❑ 4 or more

► If no, how many times do you eat them?
❑ 4 to 6 times a week
❑ 2 to 3 times a week
❑ Once a week or less
❑ Never

q98. Do you usually eat fruit (including 100% fruit juice) every day?

This group includes fruits in any form (raw or cooked, stewed, canned, frozen, in syrup, fruit salad, etc.). 100% fruit juice or no added sugar juice, pies and fruit cakes are included in this group. Nectars and fruit-based drinks are not included.

❑ Yes ❑ No

► If yes, how many times a day do you eat them?
❑ 1
❑ 2
❑ 3
❑ 4 or more

► If no, how many times do you eat them?
❑ 4 to 6 times a week
❑ 2 to 3 times a week
❑ Once a week or less
❑ Never

q99. Do you usually eat vegetables (excluding potatoes and legumes) every day?

This group includes vegetables in all their forms (smashed, canned, frozen, soups, cakes, etc.), either cooked or raw. Tomato sauce is included in this group. Potatoes are excluded.

❑ Yes ❑ No

► If yes, how many times a day do you eat them?
❑ 1
❑ 2
❑ 3
❑ 4 or more

► If no, how many times do you eat them?
❑ 4 to 6 times a week
❑ 2 to 3 times a week
❑ Once a week or less
❑ Never

q100. Do you usually eat meat, poultry, ham or eggs every day?

This group includes all types of meat, eggs in all forms, offal, etc. As for ham, this question only concerns white ham. Other cooked/prepared meats (delicatessen meats such as raw ham, sausages, meat pies, etc.) are not included in this group.

❑ Yes ❑ No

► If yes, how many times a day do you eat them?
❑ 1
❑ 2
❑ 3
❑ 4 or more

► If no, how many times do you eat them?
❑ 4 to 6 times a week
❑ 2 to 3 times a week
❑ Once a week or less
❑ Never

q101. Do you usually eat fish or other fish products every day?

This group includes all types of fish (including canned fish or breaded fish) and sea food. All dishes prepared with sea food are included as well (fishcakes, etc.)

❑ Yes ❑ No

► If yes, how many times a day do you eat them?
❑ 1
❑ 2
❑ 3
❑ 4 or more

► If no, how many times do you eat them?
❑ 4 to 6 times a week
❑ 2 to 3 times a week
❑ Once a week or less
❑ Never

q102. Do you usually eat legumes (beans, lentils, chickpeas, etc.) every day?

This group includes all legumes (chickpeas, peas, beans, corn, lentils, white, beans, red beans, broad beans) and the dishes prepared with legumes (couscous with chickpeas, etc.).

❑ Yes ❑ No

► If yes, how many times a day do you eat them?
❑ 1
❑ 2
❑ 3
❑ 4 or more

► If no, how many times do you eat them?
❑ 4 to 6 times a week
❑ 2 to 3 times a week
❑ Once a week or less
❑ Never

q103. Do you usually drink sugary drinks every day?

This group includes sodas, syrups, nectars… 100% fruit juices are not included in this group.

❑ Yes ❑ No

► If yes, how many times a day do you drink them?

► If no, how many times do you drink them?
❑ 4 to 6 times a week

❑ 1
❑ 2
❑ 3
❑ 4 or more

❑ 2 to 3 times a week
❑ Once a week or less
❑ Never

q104. Do you usually eat burgers, kebab, pizzas…every day?

❑ Yes ❑ No

► If yes, how many times a day do you eat them?
❑ 1
❑ 2
❑ 3
❑ 4 or more

► If no, how many times do you eat them?
❑ 4 to 6 times a week
❑ 2 to 3 times a week
❑ Once a week or less
❑ Never

q105. Do you usually eat sweets, pastries, chocolate bars, or cakes between meals every day?

❑ Yes ❑ No

► If yes, how many times a day do you eat them?
❑ 1
❑ 2
❑ 3
❑ 4 or more

► If no, how many times do you eat them?
❑ 4 to 6 times a week
❑ 2 to 3 times a week
❑ Once a week or less
❑ Never

q106. Do you usually eat fatty and salty products like chips, crackers, sausages, etc. every day?

❑ Yes ❑ No

► If yes, how many times a day do you eat them?
❑ 1
❑ 2
❑ 3
❑ 4 or more

► If no, how many times do you eat them?
❑ 4 to 6 times a week
❑ 2 to 3 times a week
❑ Once a week or less
❑ Never

q107. Do you feel that your diet is healthy and balanced overall?

❑ Yes ❑ No

►If no, please state why:
❑ Financial problems
❑ Hard to access good products (supermarket, minimarket)
❑ I don’t know what a healthy and balanced diet is
❑ I don’t have time to cook
❑ I’m not worried about it
❑ By choice (I can’t stop eating certain items)

- [ ] Another reason

q108. Have you ever made yourself vomit because you didn’t like the feeling of being full?

- [ ] Yes - [ ] No

q109. Do you worry about how much you eat?

- [ ] Yes - [ ] No

q110. Have you recently lost more than 6 kilos in less than 3 months?

- [ ] Yes - [ ] No

q111. Do you think you are overweight even if others think you are thin?

- [ ] Yes - [ ] No

q112. Would you say that food plays a central role in your life?

- [ ] Yes - [ ] No

Your tobacco consumption

q113. Do you smoke tobacco (cigarettes and/or rolling tobacco) regularly or occasionally?

❑ Yes ❑ Yes, but trying to stop ❑ No

► **If no**
Have you ever smoked?

❑ No, I've never smoked
❑ Yes, I've smoked occasionally
❑ Yes, I've smoked occasionally for at least 6 months

► **If "Yes" and "Yes, but trying to stop"**

►**When did you start smoking?:** |__|__| years

►**How many cigarettes do you smoke on average?**

|__|__| a day

Or |__|__| per week

Or |__|__| per month

► **"Yes" only**

►**Do you want to stop smoking?**

❑ Yes by the end of this year ❑ Yes, but later in life ❑ No

❑ Don't know

q114. During the last 12 months, have you used the following on multiple occasions:

Shisha	❑ Yes	❑ No
Chewing tobacco	❑ Yes	❑ No

Your alcohol consumption

q115. How often do you drink alcohol (alcoholic drinks such as beer, wine, whiskey, vodka, tequila, cocktails)?

❑ Never *(If never, following question = q125)*
❑ Once a year
❑ Several times a year
❑ Once a month
❑ Once a week or less
❑ 2 to 3 times a week
❑ 4 to 6 times a week
❑ Every day

q116. How often do you have at least 6 drinks on a single occasion (e.g. party)?

❑ Never
❑ Once a year
❑ Several times a year
❑ Once a month
❑ Once a week or less
❑ 2 to 3 times a week
❑ 4 to 6 times a week
❑ Every day

q117. How often during the last year have you found that you were not able to stop drinking once you had started?

❑ Never
❑ Once a year
❑ Several times a year
❑ Once a month
❑ Once a week or less
❑ 2 to 3 times a week
❑ 4 to 6 times a week
❑ Every day

q118. How often during the last year have you needed a first drink in the morning to get yourself going after a heavy drinking session?

❑ Never
❑ Once a year
❑ Several times a year
❑ Once a month
❑ Once a week or less
❑ 2 to 3 times a week
❑ 4 to 6 times a week
❑ Every day

q119. How often <u>during the last year</u> have you felt guilt or shame after drinking?

- ❑ Never
- ❑ Once a year
- ❑ Several times a year
- ❑ Once a month
- ❑ Once a week or less
- ❑ 2 to 3 times a week
- ❑ 4 to 6 times a week
- ❑ Every day

q120. How often have you been drunk <u>in the last year</u>?

- ❑ Never
- ❑ Once a year
- ❑ Several times a year
- ❑ Once a month
- ❑ Once a week or less
- ❑ 2 to 3 times a week
- ❑ 4 to 6 times a week
- ❑ Every day

q121. How often <u>during the last year</u> have you been unable to remember what happened the night before because you had been drinking?

- ❑ Never
- ❑ Once a year
- ❑ Several times a year
- ❑ Once a month
- ❑ Once a week or less
- ❑ 2 to 3 times a week
- ❑ 4 to 6 times a week
- ❑ Every day

q122. Have you or someone else been injured as a result of your drinking?

❑ Yes ❑ No ❑ Do not wish to respond

q123. Have you ever felt the need to reduce your alcohol consumption?

❑ Yes ❑ No

q124. Has a relative, friend, or doctor ever been concerned about your drinking or suggested that you cut down?

❑ Yes ❑ No

Your consumption of psychoactive substances

q125. Have you ever smoked cannabis (joint, hashish, weed, etc.)

❑ Yes ❑ No ❑ Do not wish to respond

(If "No" or "Do not wish to respond", next question = 127)

►**If yes:**

q126. Have you smoked cannabis during the last 12 months?

❑ Yes ❑ No ❑ Do not wish to respond

►**If yes**, please specify the frequency:

❑ Everyday ❑ Several times a week

❑ Once a week ❑ Several times a month

❑ At least once a month ❑ Only once

❑ Occasionally (e.g. parties with friends)

q127. Have you ever consumed any of the following:

	No never	Only once to try	More than once	Do not wish to respond
**Ecstasy, MD, MDMA**	❑	❑	❑	❑
**If "more than once":**				
**During the last 12 months, how many times have you taken…**	**Once** ❑	**Between 1 and 10 times** ❑	**More than 10 times** ❑	
**Amphetamines (Speed)**	❑	❑	❑	❑
**If "more than once":** *as previously*				
**Nitrous oxide (laughing gas)**	❑	❑	❑	❑
**If "more than once":** *as previously*				
**Inhalants (e.g. poppers)**	❑	❑	❑	❑
**If "more than once":** *as previously*				
**Cocaine**	❑	❑	❑	❑
**If "more than once":** *as previously*				

Or

❑ Do not wish to respond (to all items in this table)

q128. Have you ever taken other psychoactive substances (drugs)?

❑ Yes ❑ No ❑ Do not wish to respond

►**If yes**, please specify from the following list:

	Yes	No	Do not wish to respond
Hallucinogenic mushrooms (or other hallucinogenic plants)	❑	❑	❑

Crack cocaine, freebase cocaine	❑	❑	❑
Heroin	❑	❑	❑
LSD			
Ketamine	❑	❑	❑
Gamma-Hydroxybutyric acid (GHB)	❑	❑	❑
Other drugs - please specify: ………………………	❑	❑	❑

Or

❑ Do not wish to respond (to all items in this table)

q129. Do you think you may have taken psychoactive substances without your knowledge?

❑ Yes ❑ No ❑ Don't know ❑ Do not wish to respond

The following question is not shown if q125, 127 and 128 = No

q130. If you had already consumed drugs, has your drug use changed since you arrived at university?

❑ No, it has not changed

❑ Yes, it has increased

❑ Yes, it has decreased

❑ Yes, I've tried new ones

Your use of medicines

q131. Are you currently regularly taking any medicine (for one or more health problems)?

❒ Yes ❒ No

►<u>If yes</u>, is it (multiple options possible):

❒ Prescription medicine

❒ Over the counter medicine

❒ Medication borrowed from others

❒ Other

q132. <u>During the last 3 months</u>, have you taken any of the following medicines or products:

<u>a) Painkillers</u>

❒ Yes ❒ No

►**<u>If yes,</u>** please specify

- The frequency of use:
  - ❒ At least once a day
  - ❒ Several times a week
  - ❒ Several times a month
  - ❒ Once or less than once a month
- The type of medication (multiple answers possible):
  - ❒ Prescription medicines
  - ❒ Over the counter medicines
  - ❒ Medication borrowed from others
  - ❒ Other

<u>b) Medicines for anxiety, anguish or stress (during the day)?</u>

❒ Yes ❒ No

►**<u>If yes,</u>** please specify

- The frequency of use:
  - ❒ At least once a day
  - ❒ Several times a week
  - ❒ Several times a month
  - ❒ Once or less than once a month
- The type of medicine (multiple answers possible):
  - ❒ Prescription medicines
  - ❒ Over the counter medicines
  - ❒ Medication borrowed from others
  - ❒ Other

<u>c) Sleeping pills</u>

❒ Yes ❒ No

►**<u>If yes,</u>** please specify

- The frequency of use:
  - ❒ At least once a day
  - ❒ Several times a week
  - ❒ Several times a month
  - ❒ Once or less than once a month
- The type of medicine (multiple answers possible):
  - ❒ Prescription medicines
  - ❒ Over the counter medicines
  - ❒ Medication borrowed from others
  - ❒ Other

q133. During exams, have you ever taken any products to improve concentration?

❒ Yes ❒ No

►**<u>If yes,</u>** how often?

❒ Regularly
❒ Sometimes
❒ Occasionally

►**<u>If yes</u>**, which type of product:

❒ Medicines (prescription drugs, over-the-counter drugs, borrowed medication)
❒ Homeopathy, herbal medicine, etc.
❒ Energy drinks (containing taurine, caffeine, etc.)
❒ Other products - please specify: ……………………………………..

q134. Have you ever taken any products to improve your performance in sports?

❒ Yes ❒ No

►**<u>If yes,</u>** how frequently?

❒ Regularly
❒ Sometimes
❒ Occasionally

►**<u>If yes</u>**, preferably which type of product:

❒ Medicines (prescription drugs, over-the-counter drugs, borrowed medication)
❒ Homeopathy, herbal medicine
❒ Energy drinks (containing taurine, caffeine, etc.)
❒ Other products - please specify: ……………………………………..

q135. Have you taken vitamins or other food supplements over the last 3 months?

❒ Yes ❒ No

►**<u>If yes</u>**, can you please specify if you have taken:

- ❒ Vitamin D
- ❒ Vitamin E
- ❒ Other vitamins (A, C, etc.)
- ❒ A mix of vitamins
- ❒ Omega-3
- ❒ Other
