# Supplementary material for: Prevalence, lived experiences and user profiles in e-cigarette use: A mixed methods study among French college students
Source: PLoS One. 2024 Feb 9;19(2):e0297156. doi: 10.1371/journal.pone.0297156 (PMC10857705; doi:10.1371/journal.pone.0297156)
Supplement: Attachment — Submitted filename: PONE-D-21-33747.pdf [file pone.0297156.s007.pdf]

PLOS ONE

Prevalence, lived experiences and user profiles in e-cigarette use: A mixed methods study among French college students.

--Manuscript Draft--

**Manuscript Number:**	PONE-D-21-33747
**Article Type:**	Research Article
**Full Title:**	Prevalence, lived experiences and user profiles in e-cigarette use: A mixed methods study among French college students.
**Short Title:**	E-cigarette use among French college students
**Corresponding Author:**	Shérazade KINOUANI, Ph.D., M.D.<br>Université de Bordeaux: Universite de Bordeaux<br>Bordeaux, --- Select One --- FRANCE
**Keywords:**	e-cigarettes, smoking, students, young adults, mixed methods
**Abstract:**	Background: Little is known about e-cigarette use in French students. Our aims were to estimate the prevalence of e-cigarette experimentation and current e-cigarette use; describe the reasons for using e-cigarettes; explore the vaping experience and identify the profiles of e-cigarette users.<br>Methods: We used a sequential, explanatory mixed methods design in a sample of French university students. Quantitative data was collected online for a cross-sectional analysis among 1698 students. An analysis based on the method of Grounded Theory was then performed in 20 semi-structured interviews of former and current smokers.<br>Results: The prevalence of e-cigarette experimentation was 39.3% (95% CI: 35.2 – 44.0) and 5.1% (95% CI: 3.2 – 8.0) of students were current e-cigarette users. Reasons for using e-cigarettes were manifold, and not just limited to quitting smoking. Among former and current smokers, becoming an e-cigarette user was determined by the perceived properties of vaping products, social interactions, personal ways of learning to practice and self-image. In this context, three distinct groups of e-cigarette users were identified, based on tobacco and e-cigarette use, the functions assigned to e-cigarettes, and intentions with regards to vaping in the future.<br>Conclusion: While many smokers and former smokers have tried e-cigarettes in this student population, few have continued to use them continuously. Longitudinal studies are needed in young adult smokers for a better understanding of how their tobacco and e-cigarette use affect each other over time.
**Order of Authors:**	Shérazade KINOUANI, MD, PhD
	Héléna Da Cruz
	Emmanuel Langlois, PhD
	Christophe Tzourio, MD, PhD
**Additional Information:**	
**Question**	**Response**
**Financial Disclosure**<br><br>Enter a financial disclosure statement that describes the sources of funding for the work included in this submission. Review the submission guidelines for detailed requirements. View published research articles from *PLOS ONE* for specific examples.<br><br>This statement is required for submission and **will appear in the published article** if	This work was supported by the French National Cancer Institute [grant number INCa_11502]. The i-Share team is currently supported by an unrestricted grant of the Nouvelle-Aquitaine Regional Council (Conseil Régional Nouvelle-Aquitaine) [grant number: 4370420] and by the Bordeaux "Initiatives d'excellence" (IdEx) program of the University of Bordeaux [ANR-10-IDEX-03-02]. It has also received grants from the Nouvelle-Aquitaine Regional Health Agency (Agence Régionale de Santé Nouvelle-Aquitaine) and Public Health France (Santé Publique France). The funding bodies had no role in study design, data collection, analysis and interpretation, decision to publish or preparation of the manuscript.

Powered by Editorial Manager® and ProduXion Manager® from Aries Systems Corporation

the submission is accepted. Please make sure it is accurate.<br><br>**Unfunded studies**<br>Enter: *The author(s) received no specific funding for this work.*<br><br>**Funded studies**<br>Enter a statement with the following details:<br>• Initials of the authors who received each award<br>• Grant numbers awarded to each author<br>• The full name of each funder<br>• URL of each funder website<br>• Did the sponsors or funders play any role in the study design, data collection and analysis, decision to publish, or preparation of the manuscript?<br>• **NO** - Include this sentence at the end of your statement: *The funders had no role in study design, data collection and analysis, decision to publish, or preparation of the manuscript.*<br>• **YES** - Specify the role(s) played.<br><br>* typeset	
**Competing Interests**<br><br>Use the instructions below to enter a competing interest statement for this submission. On behalf of all authors, disclose any competing interests that could be perceived to bias this work—acknowledging all financial support and any other relevant financial or non-financial competing interests.<br><br>This statement is required for submission and **will appear in the published article** if the submission is accepted. Please make sure it is accurate and that any funding sources listed in your Funding Information later in the submission form are also declared in your Financial Disclosure statement.<br><br>View published research articles from *PLOS ONE* for specific examples.	The authors have declared that no competing interests exist.

**NO authors have competing interests**<br><br>Enter: *The authors have declared that no competing interests exist.*<br><br>**Authors with competing interests**<br><br>Enter competing interest details beginning with this statement:<br><br>*I have read the journal's policy and the authors of this manuscript have the following competing interests: [insert competing interests here]*<br><br>* typeset	
**Ethics Statement**<br><br>Enter an ethics statement for this submission. This statement is required if the study involved:<br><br>• Human participants<br>• Human specimens or tissue<br>• Vertebrate animals or cephalopods<br>• Vertebrate embryos or tissues<br>• Field research<br><br>Write "N/A" if the submission does not require an ethics statement.<br><br>General guidance is provided below. Consult the submission guidelines for detailed instructions. **Make sure that all information entered here is included in the Methods section of the manuscript.**	This research was conducted in accordance with the Declaration of Helsinki. All subjects in the quantitative phase gave their informed consent before participating in the i-Share project and ancillary studies. The i-Share project protocol was approved by the Commission Informatique et Libertés, the national authority that ensures that data collection in research does not violate freedoms, rights, and human privacy (number: DR-2013-019). Students participating in the online study on e-cigarettes received points that can be exchanged for cinema tickets or fruit and vegetable hampers. The qualitative phase received ethical approval from the ethics committee of Bordeaux University Hospital, France (number: GP-CE 2018/17). All subjects gave their oral consent just before audio-recording.

Powered by Editorial Manager® and ProduXion Manager® from Aries Systems Corporation

**Format for specific study types**<br><br>**Human Subject Research (involving human participants and/or tissue)**<br>• Give the name of the institutional review board or ethics committee that approved the study<br>• Include the approval number and/or a statement indicating approval of this research<br>• Indicate the form of consent obtained (written/oral) or the reason that consent was not obtained (e.g. the data were analyzed anonymously)<br><br>**Animal Research (involving vertebrate animals, embryos or tissues)**<br>• Provide the name of the Institutional Animal Care and Use Committee (IACUC) or other relevant ethics board that reviewed the study protocol, and indicate whether they approved this research or granted a formal waiver of ethical approval<br>• Include an approval number if one was obtained<br>• If the study involved *non-human primates*, add *additional details* about animal welfare and steps taken to ameliorate suffering<br>• If anesthesia, euthanasia, or any kind of animal sacrifice is part of the study, include briefly which substances and/or methods were applied<br><br>**Field Research**<br><br>Include the following details if this study involves the collection of plant, animal, or other materials from a natural setting:<br>• Field permit number<br>• Name of the institution or relevant body that granted permission	
**Data Availability**<br><br>Authors are required to make all data underlying the findings described fully available, without restriction, and from the time of publication. PLOS allows rare exceptions to address legal and ethical concerns. See the PLOS Data Policy and FAQ for detailed information.	No - some restrictions will apply

A Data Availability Statement describing where the data can be found is required at submission. Your answers to this question constitute the Data Availability Statement and **will be published in the article**, if accepted.<br><br>**Important:** Stating 'data available on request from the author' is not sufficient. If your data are only available upon request, select 'No' for the first question and explain your exceptional situation in the text box.<br><br>Do the authors confirm that all data underlying the findings described in their manuscript are fully available without restriction?	
**Describe where the data may be found in full sentences. If you are copying our sample text, replace any instances of XXX with the appropriate details.**<br><br>• If the data are **held or will be held in a public repository**, include URLs, accession numbers or DOIs. If this information will only be available after acceptance, indicate this by ticking the box below. For example: *All XXX files are available from the XXX database (accession number(s) XXX, XXX.*).<br>• If the data are all contained **within the manuscript and/or Supporting Information files**, enter the following: *All relevant data are within the manuscript and its Supporting Information files.*<br>• If neither of these applies but you are able to provide **details of access elsewhere**, with or without limitations, please do so. For example:<br><br>*Data cannot be shared publicly because of [XXX]. Data are available from the XXX Institutional Data Access / Ethics Committee (contact via XXX) for researchers who meet the criteria for access to confidential data.*<br><br>*The data underlying the results presented in the study are available from (include the name of the third party*	The quantitative data belong to I-share investigators. The qualitative date are in French Language and are available on request from the corresponding author

and contact information or URL).<br>• This text is appropriate if the data are owned by a third party and authors do not have permission to share the data.<br><br>* typeset	
Additional data availability information:	

Manuscript

Click here to access/download;Manuscript;Manuscript-PLOS_ONE_V1.docx

Prevalence, lived experiences and user profiles in e-cigarette use: A mixed methods study among French college students.

Shérazade Kinouani[1,2]*, Héléna Da Cruz[1], Emmanuel Langlois[3], Christophe Tzourio[1]

[1] University of Bordeaux, Inserm, Bordeaux Population Health Research Center, team HEALTHY, UMR 1219, Bordeaux, France

[2] Department of general practice, University of Bordeaux, Bordeaux, France

[3] University of Bordeaux, CNRS, Emile Durkheim Center, UMR 5116, Bordeaux, France

Short Title: E-cigarette use among French college students

*Corresponding Author:

E-mail: sherazade.kinouani@u-bordeaux.fr (SK)

Abstract

Background: Little is known about e-cigarette use in French students. Our aims were to estimate the prevalence of e-cigarette experimentation and current e-cigarette use; describe the reasons for using e-cigarettes; explore the vaping experience and identify the profiles of e-cigarette users.

Methods: We used a sequential, explanatory mixed methods design in a sample of French university students. Quantitative data was collected online for a cross-sectional analysis among 1698 students. An analysis based on the method of Grounded Theory was then performed in 20 semi-structured interviews of former and current smokers.

Results: The prevalence of e-cigarette experimentation was 39.3% (95% CI: 35.2 – 44.0) and 5.1% (95% CI: 3.2 – 8.0) of students were current e-cigarette users. Reasons for using e-cigarettes were manifold, and not just limited to quitting smoking. Among former and current smokers, becoming an e-cigarette user was determined by the perceived properties of vaping products, social interactions, personal ways of learning to practice and self-image. In this context, three distinct groups of e-cigarette users were identified, based on tobacco and e-cigarette use, the functions assigned to e-cigarettes, and intentions with regards to vaping in the future.

Conclusion: While many smokers and former smokers have tried e-cigarettes in this student population, few have continued to use them continuously. Longitudinal studies are needed in young adult smokers for a better understanding of how their tobacco and e-cigarette use affect each other over time.

Keywords: e-cigarettes, smoking, students, young adults, mixed methods.

Introduction

E-cigarette use (or vaping) has increased worldwide, particularly among young adults [1, 2]. Although toxicological studies suggest it is less harmful than smoking, the risks of long-term on human health are as yet unknown [3]. Several observational studies have suggested that vaping could be associated with a later cigarette smoking in non-smokers [4, 5] or a later risk of relapse in former smokers [6, 7]. It is probable that the risk-benefit balance of chronic vaping will always be disadvantageous in non-smokers who vape, as well as in current smokers who do not quit smoking completely, engaging in dual use of tobacco and vaping [8 9]. Despite these uncertainties, e-cigarettes are reported to be the most popular tool among young Europeans aged 15-24 when trying to quit smoking [10].

give facts and figures of vaping and cigarette smoking

? proportion of willing to quit smoking

In France, e-cigarettes are not medical devices but consumer products whose use, sale and advertising remain strongly regulated. Vaping products (e-liquids and electronic devices) on the French market comply with European regulations setting maximum nicotine content in e-liquids at 20 mg/ml [11]. Vaping is authorized everywhere, except in places frequented by minors, closed spaces or places where use is prohibited by the internal rules of the establishment. Selling and advertising to people under 18 is also prohibited, even online. Compared to other countries, the French regulatory context can be seen as “moderate” regarding e-cigarettes. Unlike the United Kingdom, neither the legislative framework nor the health agencies promote e-cigarettes as a smoking cessation tool. However, they are not considered tobacco products, as in Mexico or Turkey. We wondered how e-cigarettes were perceived and used by young adults in this particular context. Our general aim was to describe e-cigarette use in a French student population. The specific objectives were: i) estimating prevalence of e-cigarette experimentation and current e-cigarette use; ii) describing reasons for using e-cigarettes; iii) exploring vaping experiences and identifying e-cigarette user profiles in current and former smokers.

Materials and methods

only mention reference citation

We conducted a sequential explanatory mixed methods study: QUANT → qual [12, 13]. This article follows STROBE recommendations for reporting observational studies [14] and COREQ for reporting qualitative research [15].

Data sources and participants in the quantitative phase

We carried out an online quantitative study among students who had already participated in the i-Share research project (Internet-based Students Health Research Enterprise), an ongoing e-cohort of French-speaking students that started in February 2013 (www.i-share.fr). To be included, the volunteers had to be at least 18, declare themselves enrolled in a higher education institution, and know how to read and understand French. Students included in the i-Share project for at least one month were contacted by e-mail in February 2016 to participate in an ancillary study on vaping. This analysis includes volunteers from one French university (University of Bordeaux) participating in this ancillary study between February and April 2016.

Data collection and participants in the qualitative phase

Students at University of Bordeaux were invited by an advertisement via university social networks or e-mails. Those who answered the quantitative phase were also contacted by e-mail if they had reported trying e-cigarettes. Finally, we asked interviewed students if they knew other users who might be interested in participating (snowball strategy). To be included, volunteers had to be current e-cigarette users or have used them frequently for at least two continuous months, be studying at Bordeaux University, and be current or former smokers. We obtained a purposive sample based on three criteria: gender, field of study and smoking status. Smoking status included recent former smokers (had quit less than one year

before), older former smokers (had quit for at least one year or more), occasional smokers (smoked less than one cigarette per day) and daily smokers.

Semi-structured individual interviews took place between April 2016 and June 2017. They were audio-recorded and then transcribed. The guide was drafted by the authors based on the literature and the first results of the quantitative phase. It was then modified as interviews were conducted and new hypotheses emerged. This final version is available on request from the first author. At the end of the eighteenth interview, we seemed to have reached data saturation. Two other interviews were added, without any new ideas emerging.

Analyses

Quantitative component

sample size calculation?

The main outcomes were experimentation with e-cigarettes (defined as trying at least once in a lifetime) and current use of e-cigarettes (defined as daily or occasional use of e-cigarettes). We analyzed sociodemographic, academic and medical characteristics from the i-Share baseline questionnaire. We also analyzed ancillary study data on smoking status and e-cigarette use (age of first try, reasons for trying e-cigarettes, current use). In current e-cigarette users, we explored reasons for using e-cigarettes, use frequency, nicotine use in e-liquids, vaping places and times. We described continuous variables using median and interquartile range (IQR) and categorical variables using numbers and proportions. Bivariate comparison was performed by chi-square test. First, we described the sociodemographic, academic and medical characteristics in the whole sample. Secondly, we estimated the prevalence of e-cigarette experimentation and current e-cigarette use with 95% confidence interval (95% CI). We calculated them before and after weighting by calibration on the known margins of the student population at the University for the 2015-2016 academic year. This calibration was carried out with a program developed by the French National Institute for Statistics and Economic Studies: the macroSAS CALMAR® [16]. Calibration variables were

gender, age and study fields. All p-values were two-tailed and we considered $p < 0.05$ to be statistically significant. All statistical analyses were performed with R® (version 4.0.2). Thirdly, we described e-cigarette use in those students who have experimented with it and associations between e-cigarette use and smoking status were analyzed in this subsample.

Qualitative component

A pseudonym was assigned to each participant during interview transcription and used when attributing quotes. We carried out a thematic analysis of reasons for using e-cigarettes, concurrently with the end of data collection in the quantitative phase. An analysis based on Grounded Theory was then performed to understand the lived experiences and user profiles [17]. Analyses were carried out by five trained researchers, either manually or using Nvivo 10 ®. Each interview was coded individually by at least two of the five researchers with iterative pooling times, until data saturation. The themes obtained as the analysis progressed and final theorizing about user profiles were discussed by all co-authors. A synthesis of the study, its results and their interpretation were e-mailed to all interviewed students in April 2018 for comment.

Integrative mixed methods analysis

We adopted a building approach to the results [13], beginning by collecting and analyzing quantitative data and then using the findings to guide data collection in the qualitative phase. The quantitative phase described the prevalence and reasons for using e-cigarettes in our student population. The qualitative phase focused on lived experiences of vaping in dual users, former smokers and relapsing smokers.

Ethics

This research was conducted in accordance with the Declaration of Helsinki. All subjects in the quantitative phase gave their informed consent before participating in the i-Share project and ancillary studies. The i-Share project protocol was approved by the *Commission*

Informatique et Libertés, the national authority that ensures that data collection in research does not violate freedoms, rights, and human privacy (number: DR-2013-019). Students participating in the online study on e-cigarettes received points that can be exchanged for cinema tickets or fruit and vegetable hampers. The qualitative phase received ethical approval from the ethics committee of Bordeaux University Hospital, France (number: GP-CE 2018/17). All subjects gave their oral consent just before audio-recording.

how took care of privacy/confidentiality issues

Results

Sample

The quantitative component comprised 5214 students invited to participate in the ancillary study on e-cigarette use; 1815 subjects answered (response rate: 34.8%) and 1698 students were finally kept for the analyses (Fig 1). More than 3/4 of these students were female and 90% were 24 or under (median age: 21; IQR: 19.0 – 23.0). Just under half (46%) were in the healthcare field and the parents of 53% of them had had higher education. Table 1 summarizes their characteristics.

Fig 1. i-Share project students participating in the ancillary quantitative study on e-cigarette use at the University of Bordeaux (France) in 2016.

Table 1. Characteristics of college students who participated in the quantitative study on e-cigarette use at the University of Bordeaux in 2016, N=1698.

**Characteristics**	**n**	**%**
Duration between the inclusion in i-Share project and those in the ancillary study, in weeks (median, IQR*)	60	25 - 102
Gender $^{\alpha}$		
Men	375	22.1
Women	1323	77.9
Age, in years $^{\alpha}$		
18 – 20	775	45.7
21 – 24	768	45.2
25 and over	155	9.1
Academic study fields $^{\alpha}$		
Healthcare	774	45.6
Literature arts, Humanities and social sciences	391	23.0
Sciences	214	12.6
Economics, management and law	133	7.8
Other	186	11.0
Academic year of study $^{\alpha}$		
1st year	754	44.4

2nd year	340	20.0
3rd year	234	13.8
Beyond 3rd year	342	20.2
Other	28	1.6
Parents' educational level α		
Higher education, university	897	52.8
High school or vocational study	762	44.9
Primary education	10	0.6
I don't know	29	1.7
Diagnosis previously made by a physician α		
Headaches	355	20.9
Asthma	334	19.7
Anxiety and phobic disorders	236	13.9
Depression	174	10.2
Smoking status β		
Current smokers	833	49.3
Former smokers	164	9.7
Never smokers	694	41.0

[*] IQR: interquartile range; [α]Data collected at baseline in the i-Share project between February 2013 and January 2016; [β]Data collected in the online quantitative study on e-cigarette use.

As shown in Table 2, 20 students were interviewed in the qualitative component, 11 men and 9 women (median age: 26; IQR: 23.7 – 28.0). Fourteen students were former smokers and six were dual users, combining vaping with smoking. One of these dual users was a relapsed tobacco user.

Table 2. Characteristics of college students participating in the qualitative study on e-cigarette use at the University of Bordeaux in 2016-2017, N=20.

**Characteristics**	**n (%)**
**Age, in years**	
19 - 25	10 (50)
26 - 29	10 (50)
**Gender**	
Men	11 (55)
Women	9 (45)
**Participants in i-Share project** $^{\alpha}$	7 (35)
**Academic study fields**	
Healthcare	11<br>*Medicine: 9; Public health: 1; Pharmacy: 1*
Humanities and social sciences	2
Sciences	3<br>*Life sciences: 2; IT *: 1*
Economics, management and law	2
Literature and arts	2
**Smoking status**	
Former smokers, > 1 year	8 (40)
Former smokers, ≤ 1 year	6 (30)
Daily smokers (≥ 1 cigarette per day)	3 (15)
Occasional smokers (< 1 cigarette per day)	3 (15)
**E-cigarette use**	

Former users	4 (20)
Daily users (≥ 1 inhalation per day)	14 (70)
Occasional users (<1 inhalation per day)	2 (10)

*$^{\alpha}$ Seven college students participating in i-Share project answered the online ancillary study about e-cigarette use and also accepted to be interviewed in the qualitative phase; * IT: information technology*

Experimentation

The weighted prevalence of e-cigarette experimentation in the student population in the quantitative phase was 39.3%, 95% CI: 35.2 – 44.0 (Table 3). The median age of first trying e-cigarettes was 20, IQR: 18.0 – 21.0. There was more experimentation in former and current smokers than in never smokers (weighted proportion respectively: 55.5%; 52.6%; 17.4%; p<0.0001).

Table 3. Prevalence of e-cigarette use among college students at the University of Bordeaux in 2016, N=1694*

E-cigarette use	n	Crude %	95% CI $^{\beta}$	Weighted % $^{\alpha}$	95% CI $^{\beta}$
No experiment $^{\sigma}$	991	58.5	56.1 – 60.9	55.6	51.3 – 60.0
Experiment $^{\gamma}$	645	38.1	35.8 – 40.4	39.3	35.2 – 44.0
Current use $^{\omega}$	58	3.4	2.6 – 4.4	5.1	3.2 – 8.0
Occasional use	28	1.6	1.1 – 2.4	2.4	1.2 – 5.0
Daily use	30	1.6	1.2 – 2.5	2.7	1.5 – 5.0

**1698 subjects included in analysis but only data concerning 1694 subjects were available on e-cigarette use and calibration variables. $^{\alpha}$ Weighting by calibration on margins, using the MacroSAS Calmar® program (raking ratio method). The calibration variables were: gender, age and the study fields; $^{\beta}$95% confidence interval; $^{\sigma}$ Never tried to use e-cigarettes; $^{\gamma}$ Having tried at least once to use e-cigarettes; $^{\omega}$ Occasional (<1 time per day) or daily (≥ 1 time per day) use of e-cigarettes.*

Reasons for trying e-cigarettes were often unrelated to smoking cessation, even in those who had quit smoking (S1 Table). Students reported curiosity, diverse flavors and the playful aspect as the main factors influencing their first try. Few students had tried e-cigarettes with a view to quitting or cutting down smoking. The cost, lesser perceived harmfulness, similar gesture to smoking or possibility of vaping in places where smoking is forbidden were factors influencing their vaping initiation. Friends, family or a partner who already used e-cigarettes contributed to their initiation.

What were the main themes driven out here

Mention the topic guide for interviews

Current use

Few students who had tried e-cigarettes were still using them currently. The weighted prevalence of current use in the student population was 5.1%, (95% CI: 3.2 – 8.0) (Table 3). Current use was most frequent in former smokers, followed by current smokers, but was rare in never smokers (weighted proportions respectively: 14.6%; 6.7%; 0.2%; $p<0.0001$). The majority of the 58 current users in the quantitative study vaped e-liquids containing nicotine, but five did not know if their e-liquids contained nicotine (S1 Table).

There was rarely a single reason for those vaping currently (S1 Table). Whether dual users or former smokers, e-cigarette use was explained mainly by comparing to tobacco. It was a way for some students to maintain their smoking habits, even when no longer smoking tobacco. Some said it helped break the day up. Others said it allowed them to continue enjoying the same gestures and sensations. It also produces the psychotropic effects of nicotine and helps users focus while working, but also relax. Many downsides of smoking were circumvented by e-cigarette use. Some saw e-cigarettes as less troublesome for those around them, owing to the lack of smell, or as a way around the ban on smoking in public places. Because they provide nicotine, e-cigarettes were also seen as a good substitute allowing a gradual reduction in tobacco or nicotine use, sometimes until cessation. Other reasons were unrelated to smoking, such as the possibility to customize use or the fact that vaping becomes a pleasure or leisure behavior.

What about thematic/content analysis

Lived experiences: vaping but not necessarily being a vaper

Becoming a current e-cigarette user

Students sometimes used a friend’s or relative's device during experimentation, but had to have their own device for regular use. They made their first purchase in stores or on Internet; their first personal e-cigarette was rarely a gift. Over time, they changed devices and opted for technically more efficient models, offering better rendering of flavors, aerosol density, throat hit or battery life.

Current e-cigarette users often showed little interest in knowing more about vaping. When they did inquire, they mainly wanted information about the health effects, variety of flavors and ways to improve device performances or reduce the cost of use. This lack of interest in staying informed was claimed by some students as a way to keep a distance from their own usage and other users (the vaping community).

Three properties mainly characterized e-cigarette use: discretion, availability, and above all, technicality. Discretion is ensured by less smoke production, no smell on clothing, or the small size of devices. It is easy to go to a store for devices, spare parts or e-liquid refills, or to buy them online.

"*I find that either ordering on Internet or going to a real store where you can try several tastes and talk to the seller ... well, it's more in the spirit of electronic cigarettes than going to buy an electronic cigarette and e-liquid from the tobacconist* " (Tao: man, former smoker and e-cigarette user for 4 months).

The technicality of devices was both a strength and weakness. Users needed to learn how to inhale the aerosol correctly for the expected effects, as with tobacco cigarettes, and also how to set and maintain their electronic device. This generated recurring malfunctions,

repeated maintenance and a personal effort to acquire technical skills, which discouraged some. Levels of personal interest in knowing more about vaping and electronic devices resulted in distinct attitudes. Either partial or total smoking substitution by vaping, conserving smoking habits (frequency, times and places) as much as possible; or adopting vaping as a new behavior, different from smoking and perceived as enjoyable.

Self-image and social interactions

Vaping allowed some former smokers to manage their tobacco addiction. They had succeeded in replacing cigarettes with e-cigarettes and felt in control again. Other former smokers saw a lack of freedom, however, as e-cigarettes fed their addiction.

"*I think in a way yes, they (e-cigarettes) allowed me this first stop, and that made me say: well you can stop that (tobacco)"* (Anna: woman, former smoker who also quit e-cigarettes).

"*Well, you don't stress out about your pack of cigarettes anymore but you stress out ... if your thing (her e-cigarette) is loaded. Well, it's ... it's the same as it is ... we're just so addicted to something all the time. And then it remains the same substance: nicotine* " (Bea: woman, former smoker and e-cigarette user for 2 years).

Users felt that vaping drew attention to themselves and this was variably received. While some saw vaping as a way of giving a trendy image of themselves, others thought it made them look ridiculous, weak (vaping instead of smoking a real cigarette) or naive (vaping a dangerous product, regardless of health risk).

Non-users often showed surprise and curiosity during social interactions. They were tolerant and even wanted to know more about e-cigarette use: vaping was perceived overall as less harmful than smoking. Vaping favored social interactions between e-cigarette users. They shared knowledge, helped each other solve technical problems, talked about experiences of quitting smoking, etc. Some comments suggested membership of a subculture, notably via use of specific technical vocabulary to describe their device and practice, in particular among

former smokers with a very personalized use of e-cigarettes. They were also those who described themselves as “vapers”.

User profiles

Three groups of users were distinguished (Fig 2). Firstly, some users had succeeded in stopping smoking through e-cigarettes and saw them primarily as a technological innovation. Accumulating vaping knowledge and technical skills contributed to constructing their vaper identity. Vaping was a whole new experience, both personally and socially. They knowingly continued to use e-cigarettes after quitting smoking. Secondly, other former smokers rejected the vaper label. E-cigarettes were only a tool to stop smoking. They planned to quit smoking and achieved it through e-cigarettes or nicotine replacement therapies (if vaping was unsuccessful). These former smokers did not want vaping to participate in the construction of their identity, seeing vaping as a temporary stage. Many stopped using e-cigarettes after quitting smoking. Others continued to use e-cigarettes mainly for fear of relapse. Unlike the first two groups, common characteristics of the third group were their smoker identity and dual use. Based on the function assigned to e-cigarettes, three kinds of dual users were observed. Some perceived e-cigarettes as a tool to stop smoking and hoped that dual use was a step towards smoking cessation. Other dual users replaced tobacco by vaping except in circumstances where they felt that smoking could not be replaced, such as stressful events or evenings. The last dual users were not at all quitting or switching. E-cigarettes were an additional way of diversifying their nicotine use, particularly because of its practicality, the possibility of testing flavors, and so on.

Fig 2. Relationship between user identity, the function assigned to e-cigarettes and the change in tobacco and e-cigarette use, among current and former smokers of the University of Bordeaux N=20.

* Concomitant use of tobacco and e-cigarettes

Discussion

The quantitative study showed that two in five students have tried e-cigarettes, but occasional or daily use was reported by only 5% of students. The reasons mentioned for starting or continuing to use e-cigarettes were manifold and not just limited to quitting smoking. E-cigarettes were used by former smokers as a means either to switch to a new, lasting and pleasant behavior, or as a transitory step towards stopping smoking. Dual users, on the other hand, formed a heterogeneous group. They all partly replaced tobacco use by vaping but with various perspectives, not necessarily to quit smoking.

Our prevalence estimates of e-cigarette use are close to those described in adult populations in other high-income countries with moderately restrictive e-cigarette policies [18]. A recent study suggests that binding regulations on e-cigarettes could influence use in the adult population [19]. However, it has not been established whether they also impact current use of e-cigarettes among young adults [20]. Interpretation of e-cigarette use levels should also take tobacco control policies into account. According to the 2014 Eurobarometer survey, e-cigarette experimentation and current use among subjects aged 15 or over were higher in European countries that have increased tobacco taxes or promoted aid for smoking cessation [21]. The effectiveness of tobacco control policies is also not similar in all high-income countries. While they have succeeded in reducing the smoking rates in Canada or Australia, it remains at a high level in others countries such as France or Romania. For policymakers, e-cigarettes could be seen as detrimental to efforts that have helped reduce smoking rates, except in countries where those remain high: they may be more tolerated there as harm reduction tools [22]. A combination of high smoking rate despite strong, sustainable tobacco control policies, and moderately or slightly restrictive regulations on e-cigarettes probably constitutes in France the favorable context for vaping in the adult population.

Maintaining occasional or daily use of e-cigarettes seemed multifactorial. It was only partly linked to the addictive power of nicotine in e-liquids. Smoking, e-cigarette use and nicotine addiction were measured for four years in a longitudinal study among US adults aged 19-23 [23]. Among non-smokers, e-cigarette use was not significantly associated with subsequent tobacco use, either directly or mediated by nicotine addiction. In contrast, tobacco use was associated with subsequent e-cigarette use in smokers, both directly and through nicotine addiction. The transition from smoking to e-cigarette use was therefore only partially mediated by nicotine addiction. Our results suggest that this transition may also be related to vaping product properties and the experience during use (sensations, social interactions, self-image as a user).

Our results showed three e-cigarette user profiles among students. Previous qualitative studies have provided similar descriptions [24-27] but few specifically in young adult populations. One was a qualitative study of 20 Americans aged 21 to 27 and reported similar profiles [27].

Our analysis had some limitations. It was conducted in just one French university, limiting extrapolation to all French students or to young adults from countries with different e-cigarette regulations. Tobacco and e-cigarette use were self-reported in the quantitative study, with a risk of underestimating prevalence due to memorization bias or social desirability. Likewise, self-selection bias is possible through the voluntary participation of students. In the qualitative study, more than half of participants were healthcare students. Being future health professionals might have influenced their discourse in favor of smoking cessation benefits. Finally, the profiles of e-cigarette users in our analysis might appear frozen, but some prospective observational quantitative studies suggest that vaping is a more dynamic process in smokers, even in young adults [28-30]. Where follow-up was long enough (≥ 12 months), multiple trajectories were observed with transitions from smoker to former smoker, from smoker to vaper, and so on. In these studies, dual use was also a factor in maintenance or relapse in smoking, except perhaps when the concomitant use of e-

cigarettes was daily. Despite these weaknesses, our analysis seems to be the first to explore the relationship between e-cigarette and tobacco use in depth in a French student population, with a moderately restrictive regulation regarding e-cigarettes.

Conclusion

E-cigarette experimentation was frequent in this French student population, especially among smokers and former smokers. Current use was reported by 5% of students and was rare in non-smokers. Becoming a user was driven by the perceived properties of vaping products, social interactions, personal ways of learning to practice and self-image as a user. Three distinct groups of users were identified: smoker, former smoker and vaper. Among them, dual users formed a heterogeneous group. The functions assigned by dual users to e-cigarettes should be clarified systematically by clinicians seeking a posture that is conducive to behavior change to stop smoking. Longitudinal quantitative studies over more than one year in young adult smokers also appear necessary to understand the dynamics of their tobacco and e-cigarette use, in particular changes in identity. The regulatory effect could be investigated by carrying out studies simultaneously in countries with different e-cigarette regulations.

Acknowledgements

The authors would like to thank Ms Jacqueline Pedley and Hancock Hutton Enterprise for copyediting the manuscript. They thank the "*GROupe Universitaire de recherche qualitative Médicale Francophone*" (GROUM.F) for training the first author in the concepts and techniques of qualitative research. They thank Chloé Leflot, Louis Bétolaud du Colombier, Lucile Roussel-Dupré and Cécile Landou who participated in the data collection and analysis in the qualitative research study. They acknowledge the research team of the i-Share project for their logistical support. Finally, they thank university services that have helped them with the research and are grateful to all the participants who volunteered to take part in the study.

References

1 Laverty AA, Filippidis FT, Vardavas CI. Patterns, trends and determinants of e-cigarette use in 28 European Union Member States 2014–2017. Prev Med. 2018;116:13–8.

2 Spears CA, Jones DM, Weaver SR, Huang J, Yang B, Pechacek TF, et al. Sociodemographic correlates of Electronic Nicotine Delivery Systems (ENDS) use in the United States, 2016-2017. Am J Public Health. 2019;109(9):1224–32.

3 Helen GS, Eaton DL. Public Health Consequences of e-Cigarette Use. JAMA Intern Med. 2018;178(7):984–6.

4 Pierce JP, Chen R, Leas EC, White MM, Kealey S, Stone MD, et al. Use of e-cigarettes and other tobacco products and progression to daily cigarette smoking. Pediatrics. 2021;147(2):e2020025122.

5 Khouja JN, Suddell SF, Peters SE, Taylor AE, Munafò MR. Is e-cigarette use in non-smoking young adults associated with later smoking? A systematic review and meta-analysis. Tob Control. 2021;30(1):8–15.

6 Everard CD, Silveira ML, Kimmel HL, Marshall D, Blanco C, Compton WM. Association of Electronic Nicotine Delivery System use with cigarette smoking relapse among former smokers in the United States. JAMA Netw Open. 2020;3(6):e204813–e204813.

7 Gomajee R, El-Khoury F, Goldberg M, Zins M, Lemogne C, Wiernik E, et al. Association between electronic cigarette use and smoking reduction in France. JAMA Intern Med.2019;179(9):1193-1200.

8 Bhatta DN, Glantz SA. Association of e-cigarette use with respiratory disease among adults: a longitudinal analysis. Am J Prev Med. 2020;58(2):182–90.

9 Hair EC, Barton AA, Perks SN, Kreslake J, Xiao H, Pitzer L, et al. Association between e-cigarette use and future combustible cigarette use: evidence from a prospective cohort of youth and young adults, 2017-2019. Addict Behav. 2021;112:106593.

10 Filippidis FT, Laverty AA, Mons U, Jimenez-Ruiz C, Vardavas CI. Changes in smoking cessation assistance in the European Union between 2012 and 2017: pharmacotherapy versus counselling versus e-cigarettes. Tob Control. 2019;28(1):95–100.

11 European Commission. Tobacco - Product regulation. Available from: https://ec.europa.eu/health/tobacco/products_en. Accessed 27 November 2020.

12 Creswell JW, Clark VLP. Designing and conducting mixed methods research. Thousand Oaks, CA, US: Sage Publications, Inc; 2007.

13 Fetters MD, Curry LA, Creswell JW. Achieving Integration in Mixed Methods Designs—Principles and Practices. Health Serv Res. 2013;48(6pt2):2134–56.

14 von Elm E, Altman DG, Egger M, Pocock SJ, Gøtzsche PC, Vandenbroucke JP, et al. Strengthening the Reporting of Observational Studies in Epidemiology (STROBE) statement: guidelines for reporting observational studies. BMJ. 2007;335(7624):806–8.

15 Tong A, Sainsbury P, Craig J. Consolidated criteria for reporting qualitative research (COREQ): a 32-item checklist for interviews and focus groups. Int J Qual Health Care. 2007;19(6):349–57.

16 INSEE. La macro SAS CALMAR. Available from: https://www.insee.fr/fr/information/2021902. Accessed 28 november 2020.

17 Glaser BG, Strauss AL. The discovery of grounded theory: strategies for qualitative research. 5th edition. New Brunswick, New Jersey: Aldine Transaction; 2010.

18 Gravely S, Driezen P, Ouimet J, Quah ACK, Cummings KM, Thompson ME, et al. Prevalence of awareness, ever-use and current use of nicotine vaping products (NVPs) among adult current smokers and ex-smokers in 14 countries with differing regulations on sales and marketing of NVPs: cross-sectional findings from the ITC Project. Addiction. 2019;114(6):1060–73.

19 Du Y, Liu B, Xu G, Rong S, Sun Y, Wu Y, et al. Association of electronic cigarette regulations with electronic cigarette use among adults in the United States. JAMA Netw Open. 2020;3(1):e1920255.

20 Lee C, Yong HH, Borland R, McNeill A, Hitchman SC. Acceptance and patterns of personal vaporizer use in Australia and the United Kingdom: Results from the International Tobacco Control survey. Drug Alcohol Depend. 2018;185:142–8.

21 La Torre G, Mipatrini D. Country-level correlates of e-cigarette use in the European Union. Int J Public Health. 2016;61(2):269–75.

22 Vardavas CI, Agaku IT. Electronic cigarettes: the Issues behind the moral quandary. In: Loddenkemper R, Kreuter M, editors. Progress in Respiratory Research. Basel: Karger; 2015. Vol. 42; p. 258–67.

23 Selya AS, Rose JS, Dierker L, Hedeker D, Mermelstein RJ. Evaluating the mutual pathways among electronic cigarette use, conventional smoking and nicotine dependence. Addiction. 2018;113(2):325–33.

24 Hoek J, Thrul J, Ling P. Qualitative analysis of young adult ENDS users' expectations and experiences. BMJ Open. 2017;7(3):e014990.

25 Notley C, Ward E, Dawkins L, Holland R. User pathways of e-cigarette use to support long term tobacco smoking relapse prevention: a qualitative analysis. Addiction. 2021;116(3):596–605.

26 Tokle R, Pedersen W. 'Cloud chasers' and 'substitutes': e-cigarettes, vaping subcultures and vaper identities. Sociol Health Illn. 2019;41(5):917–32.

27 Yule JA, Tinson JS. Youth and the sociability of 'Vaping'. J Consum Behav. 2017;16(1):3–14.

28 Baig SA, Giovenco DP. Behavioral heterogeneity among cigarette and e-cigarette dual-users and associations with future tobacco use: Findings from the Population Assessment of Tobacco and Health Study. Addict Behav. 2020;104:106263.

29 Niaura R, Rich I, Johnson AL, Villanti AC, Romberg AR, Hair EC, et al. Young adult tobacco and e-cigarette use transitions: examining stability using multistate modeling. Nicotine Tob Res. 2020;22(5):647–54.

30 Sweet L, Brasky TM, Cooper S, Doogan N, Hinton A, Klein EG, et al. Quitting behaviors among dual cigarette and e-cigarette users and cigarette smokers enrolled in the tobacco user adult cohort. Nicotine Tob Res. 2019;21(3):278–84.

Supporting information

S1 Table. Vaping among college students who had tried e-cigarettes in a quantitative phase$^{\alpha}$ (N=704) and qualitative phase$^{\beta}$ (N=20) from a mixed methods study carried out at the University of Bordeaux (France), 2016-2017.

Figure

Click here to access/download;Figure;Figure 1.tiff

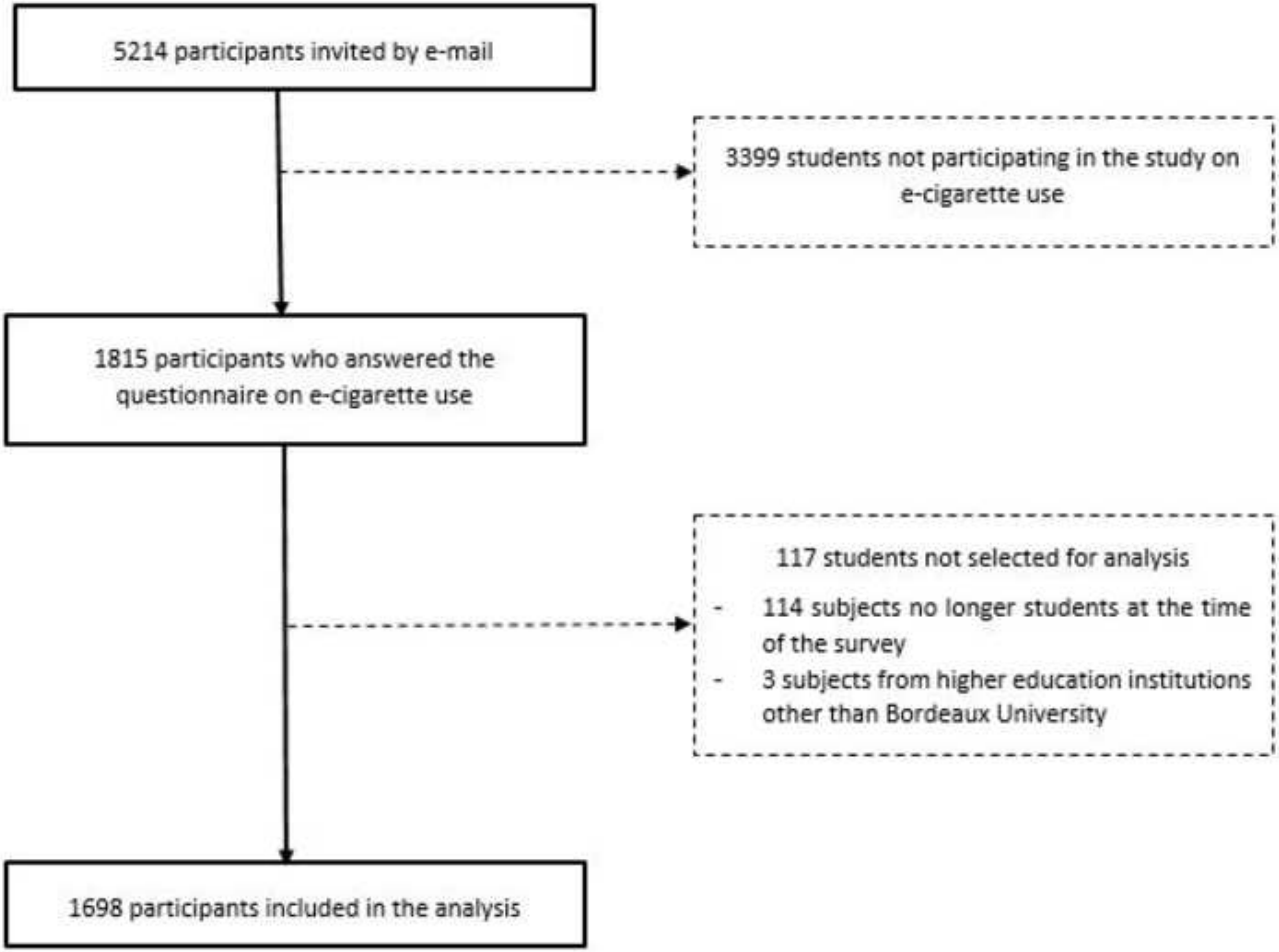

Figure

Click here to access/download;Figure;Figure 2.tiff

Identity	Vaper	Former smoker	Smoker
**Tobacco and e-cigarette use**	Exclusive use of e-cigarettes	Exclusive use of e-cigarettes	Dual use*
**Functions assigned to e-cigarettes**	Technological innovation<br>Smoking cessation tool	Smoking cessation tool	Smoking cessation tool<br>Substitution tool<br>Nicotine delivery diversification tool
**Intention about future e-cigarette use**	Pursuit	Stop	Variable

Click here to access/download
Supporting Information
S1 Table_PLOS_ONE_V1.docx
