# Supplementary material for: Prevalence, lived experiences and user profiles in e-cigarette use: A mixed methods study among French college students
Source: PLoS One. 2024 Feb 9;19(2):e0297156. doi: 10.1371/journal.pone.0297156 (PMC10857705; doi:10.1371/journal.pone.0297156)
Supplement: Attachment — Submitted filename: REVIEWERs comments 21-33747.odt [file pone.0297156.s006.odt]

REVIEWER’S COMMENTS: 
Title: “Prevalence, lived experiences and user profiles in e-cigarette use: A mixed methods study among French college students.”
The manuscript has addressed an important public health issue of e-cigarette use. Authors have used mixed methods to investigate this problem among university students. Introduction of the problem is nicely narrated though the facts and figures were found to be missing under section of “Introduction of problem”. Similarly, rationale was to be made clear before mentioning objectives of study.
Relevant statistical methods were not applied though mentioned in the methodology section, like chi square test and measures of risk for association of risk factors. Weighted prevalence according to smoking status is to be shown in the table as well. Qualitative study sample is said to be from quantitative one but differ a lot in its composition according to age and gender; that might affect interpretation of findings. Component of thematic/ content analysis should have been described in the results to display it in the form of themes rather than giving individual reason/s. Duration of data collection appear too long for 20 interviews. Strengths and limitations have to be described.
Conclusion is to be re- stated by emphasizing triangulation of findings of both quantitative and qualitative study and also separately.
References are validated and cited correctly in the text.
Overall quality of work is acceptable with major revision in analysis and presentation of results.
Thanks,
