# Supplementary material for: Prevalence, lived experiences and user profiles in e-cigarette use: A mixed methods study among French college students
Source: PLoS One. 2024 Feb 9;19(2):e0297156. doi: 10.1371/journal.pone.0297156 (PMC10857705; doi:10.1371/journal.pone.0297156)
Supplement: Attachment — Submitted filename: Responses to reviewers_PLOS_ONE_V5.docx [file pone.0297156.s008.docx]

To:

Pr Eman Sobh
Academic Editor

PLOS ONE	Bordeaux, June 26^th^, 2022

Object : Response to reviewers’

Pr Eman Sobh,

We are pleased to submit our revised manuscript entitled “**Prevalence, lived experiences and user profiles in e-cigarette use: a mixed methods study among French college students**” for consideration as publication of a research article. Here are our point-by-point responses to the reviewers whom we thank for their comments.

A. **Journal Requirements**

When submitting your revision, we need you to address these additional requirements.

1. Please ensure that your manuscript meets PLOS ONE's style requirements, including those for file naming. The PLOS ONE style templates can be found at 
https://journals.plos.org/plosone/s/file?id=wjVg/PLOSOne_formatting_sample_main_body.pdf and 
https://journals.plos.org/plosone/s/file?id=ba62/PLOSOne_formatting_sample_title_authors_affiliations.pdf

We revised the manuscript following the PLOS ONE's style requirements, including those for file naming.

2. Please include additional information regarding all the survey, questionnaires or interview guides used in the study and ensure that you have provided sufficient details that others could replicate the analyses. For instance, if you developed a questionnaire as part of this study and it is not under a copyright more restrictive than CC-BY, please include a copy, in both the original language and English, as Supporting Information.

We have attached the baseline questionnaire of the i-Share project in French (S1 file) and in English language (S2 file). All questions about the tobacco and e-cigarette use asked as part of the ancillary study to the i-Share project are directly described in the manuscript (in the "quantitative component" section of Materials and methods).

We also added the final guide of our semi-structured interviews as supporting information, in French language (S3 file) and English language (S4 file).

3. We note that the grant information you provided in the ‘Funding Information’ and ‘Financial Disclosure’ sections do not match.

When you resubmit, please ensure that you provide the correct grant numbers for the awards you received for your study in the ‘Funding Information’ section.

We checked and corrected the "Funding information" section. We have also matched the two sections "Funding information" and "Financial disclosure".

4. We note that you have indicated that data from this study are available upon request. PLOS only allows data to be available upon request if there are legal or ethical restrictions on sharing data publicly. For more information on unacceptable data access restrictions, please see http://journals.plos.org/plosone/s/data-availability#loc-unacceptable-data-access-restrictions.

In your revised cover letter, please address the following prompts:

a) If there are ethical or legal restrictions on sharing a de-identified data set, please explain them in detail (e.g., data contain potentially sensitive information, data are owned by a third-party organization, etc.) and who has imposed them (e.g., an ethics committee). Please also provide contact information for a data access committee, ethics committee, or other institutional body to which data requests may be sent.

b) If there are no restrictions, please upload the minimal anonymized data set necessary to replicate your study findings as either Supporting Information files or to a stable, public repository and provide us with the relevant URLs, DOIs, or accession numbers. For a list of acceptable repositories, please see http://journals.plos.org/plosone/s/data-availability#loc-recommended-repositories.

We will update your Data Availability statement on your behalf to reflect the information you provide.

We apologize for the misunderstanding, but we only said that the interview guide was available on request, not all the collected data. We have made the final guide available to readers as additional file 1 (in French language) and 2 (in English language). We have also provided the i-Share inclusion questionnaire as additional files 3 (in French language) and 4 (in English language).

Data cannot be shared freely as we have to comply to French regulations (the *Commission Informatique et Libertés*, which is the French authority that ensures that the collection of data in the context of research does not infringe on the freedoms, rights and privacy of individuals/number: DR-2013-019), and European regulations (General Data Protection Regulation). Thus, the qualitative component (interviews) cannot be shared but data of the quantitative component of the study are available upon reasonable request. Our statement is therefore: The i-Share project data used in the quantitative component can be requested for scientific collaboration, following a procedure described on the study website (<https://research.i-share.fr/how-to-collaborate/>). All request must be sent to the i-Share Scientific Collaborations Coordinator: Ilaria Montagni ([ilaria.montagni@u-bordeaux.fr](mailto:ilaria.montagni@u-bordeaux.fr)).

5. Thank you for stating the following financial disclosure: "This work was supported by the French National Cancer Institute [grant number INCa_11502]. The i-Share team is currently supported by an unrestricted grant of the Nouvelle-Aquitaine Regional Council (Conseil Régional Nouvelle-Aquitaine) [grant number: 4370420] and by the Bordeaux “Initiatives d’excellence” (IdEx) program of the University of Bordeaux [ANR-10-IDEX-03-02]. It has also received grants from the Nouvelle-Aquitaine Regional Health Agency (Agence Régionale de Santé Nouvelle-Aquitaine) and Public Health France (Santé Publique France). The funding bodies had no role in study design, data collection, analysis and interpretation, decision to publish or preparation of the manuscript."

We note that one or more of the authors is affiliated with the funding organization, indicating the funder may have had some role in the design, data collection, analysis or preparation of your manuscript for publication; in other words, the funder played an indirect role through the participation of the co-authors. If the funding organization did not play a role in the study design, data collection and analysis, decision to publish, or preparation of the manuscript and only provided financial support in the form of authors' salaries and/or research materials, please do the following:

a. Review your statements relating to the author contributions, and ensure you have specifically and accurately indicated the role(s) that these authors had in your study. These amendments should be made in the online form.

We checked our statements relating to the author contributions, and indicated the roles that each author had in our study.

b. Confirm in your cover letter that you agree with the following statement, and we will change the online submission form on your behalf: 
“The funder provided support in the form of salaries for authors [insert relevant initials], but did not have any additional role in the study design, data collection and analysis, decision to publish, or preparation of the manuscript. The specific roles of these authors are articulated in the ‘author contributions’ section.

We added these sentences in our cover letter.

6. Please upload a new copy of Figure 1 as the detail is not clear. Please follow the link for more information: https://blogs.plos.org/plos/2019/06/looking-good-tips-for-creating-your-plos-figures-graphics/" <https://blogs.plos.org/plos/2019/06/looking-good-tips-for-creating-your-plos-figures-graphics/>

While revising your submission, please upload your figure files to the Preflight Analysis and Conversion Engine (PACE) digital diagnostic tool, https://pacev2.apexcovantage.com/. PACE helps ensure that figures meet PLOS requirements. To use PACE, you must first register as a user. Registration is free. Then, login and navigate to the UPLOAD tab, where you will find detailed instructions on how to use the tool. If you encounter any issues or have any questions when using PACE, please email PLOS at figures@plos.org. Please note that Supporting Information files do not need this step.

We used the Preflight Analysis and Conversion Engine (PACE) to improve our figures.

-------------------------------------------------------------------------

B. **Reviewer's Responses to Questions**

**Comments to the Author**

1. Is the manuscript technically sound, and do the data support the conclusions?

The manuscript must describe a technically sound piece of scientific research with data that supports the conclusions. Experiments must have been conducted rigorously, with appropriate controls, replication, and sample sizes. The conclusions must be drawn appropriately based on the data presented.

Reviewer #1: Partly

Reviewer #2: Yes

Reviewer #3: Yes

2. Has the statistical analysis been performed appropriately and rigorously?

Reviewer #1: Yes

Reviewer #2: Yes

Reviewer #3: No

3. Have the authors made all data underlying the findings in their manuscript fully available?

The [PLOS Data policy](http://www.plosone.org/static/policies.action#sharing) requires authors to make all data underlying the findings described in their manuscript fully available without restriction, with rare exception (please refer to the Data Availability Statement in the manuscript PDF file). The data should be provided as part of the manuscript or its supporting information, or deposited to a public repository. For example, in addition to summary statistics, the data points behind means, medians and variance measures should be available. If there are restrictions on publicly sharing data—e.g. participant privacy or use of data from a third party—those must be specified.

Reviewer #1: No

Reviewer #2: Yes

Reviewer #3: Yes

4. Is the manuscript presented in an intelligible fashion and written in standard English?

PLOS ONE does not copyedit accepted manuscripts, so the language in submitted articles must be clear, correct, and unambiguous. Any typographical or grammatical errors should be corrected at revision, so please note any specific errors here.

Reviewer #1: Yes

Reviewer #2: Yes

Reviewer #3: Yes

5. Review Comments to the Author

Please use the space provided to explain your answers to the questions above. You may also include additional comments for the author, including concerns about dual publication, research ethics, or publication ethics. (Please upload your review as an attachment if it exceeds 20,000 characters)

Reviewer #1: The original sent for my review is a cross- sectional study aiming to describe the prevalence of e- cigarette users among French College students. This is an important topic because the use of these devices has led to significant debate on whether they should be considered as tobacco products or not and even if those devices are a safe way for smoking cessation.

However, due to study design, I cannot be sure that the authors have enrolled a representative population of French college students. If the authors selected those inscribed on a web page, this could have biased the population and therefore a true prevalence cannot be claimed by the authors.

We thank the reviewer for this comment. The quantitative component is indeed a cross-sectional study led in 2016 among French college students already participating in another study: the i-Share project. The i-Share project is an online study in which French-speaking students participate on a voluntary basis. This may result in a participation bias due to the self-selection of respondents. For this reason, we applied a method of calibration on margins to our data. This method allows the weight of each individual to be modified in our sample by taking into account the real composition of the target population on auxiliary data (the calibration variables). Information on the real composition of the student population is provided by the French Ministry of Higher Education and Research. It is provided sparingly upon request by researchers.

In our analysis, the calibration variables were: gender, field of study and age in classes. This method allows for in-sample estimates after adjusting the margins of a contingency table to the margins of the target population on auxiliary data; these must be available for both the sample and the target population. The sample obtained after this weighting of individuals is then representative of the target population for the calibration variables.

We have added the reference of this calibration method to our revised manuscript : Deville JC, Särndal CE, Sautory O. Generalized raking procedures in survey sampling. J Am Stat Assoc. 1993;88(423):1013-20.

To apply this statistic method, we used the MacroSAS Calmar® program. This program is described in detail in the "calibration methods" section by INSEE: https://www.insee.fr/en/information/5398341

The weighted estimators presented in Table 3 are therefore the closest estimators we can obtain of the true prevalence of e-cigarette use in the college student population of Bordeaux area, France.

Reviewer #2: The study is well-conceived, well-designed and well-performed. The conclusions are supported by results which in turn are supported by sound statistical methods. I have no major reservations or objections with the study.

We thank the reviewer for these comments.

Reviewer #3: An important topic had been studied. However, Topic guide if included would give a better idea of qualitative component. Thematic analysis is to be shown by reducing factors into major themes, as plan mentioned in methodology section but not seen in the results section. The socio- demographic factors can be analysed for their association with vaping.

We thank the reviewer for this comment. We added our final guide as supporting information (S1 and S2 files). We have presented the categories related to the experience of vaping that emerged from the grounded theory analysis: becoming a current e-cigarette user; self-image; and social interactions.

We modified the first part of the results to better highlight the main themes related to reasons for using e-cigarettes. We have added 2 figures to the manuscript to illustrate the organization into main themes on reasons for experimenting with e-cigarettes (figure 2) and reasons for current use (figure 3).

We could have described the profiles of regular e-cigarette users (those who have vaped for at least 2 months) by observing which socio-economic characteristics are associated with e-cigarette use. We did not choose this way of describing the profiles because it would not have allowed us to understand the diversity of students' experiences who don't give up after the first use. This is really what interested us because we think that this is both little studied and this diversity of experiences better explains the gap found between a strong experimentation but a weak current use in student population.

---------------------------------------------------------------

C. **Reviewer’s comments**

Title: “Prevalence, lived experiences and user profiles in e-cigarette use: A mixed methods study among French college students.”

The manuscript has addressed an important public health issue of e-cigarette use. Authors have used mixed methods to investigate this problem among university students. Introduction of the problem is nicely narrated though the facts and figures were found to be missing under section of “Introduction of problem”. Similarly, rationale was to be made clear before mentioning objectives of study.

Introduction:

We added some examples about smoking and vaping in the Introduction section. We also added information about the European young adults willing to quit smoking according to the Eurobarometer survey.

We finally added sentences before our objectives to explain why it seemed important in the context of French regulation of vaping to estimate the prevalence for using e-cigarettes and to study the reasons and practices of young adults to use them.

Relevant statistical methods were not applied though mentioned in the methodology section, like chi square test and measures of risk for association of risk factors. Weighted prevalence according to smoking status is to be shown in the table as well.

Materials and methods:

We used the chi square test to measure the association between smoking status and e-cigarette use (experimentation and current use). For this reason, we announced the application of this test in the method. We presented these measures of association in the text only so as not to overload Table 3. In addition, due to missing data on smoking status, the total sample for this stratified analysis is slightly different from that presented in Table 3 (N=1691 instead of 1694). Thus, we added a Table 4 in this revised manuscript with weighted prevalence of different use of e-cigarettes according to smoking status.

Qualitative study sample is said to be from quantitative one but differ a lot in its composition according to age and gender; that might affect interpretation of findings.

Materials and methods:

We apologize for this misunderstanding, but we never said that the sample for the qualitative study would be identical to that for the quantitative study. Our aim was to have a close-to-representative sample of the target population for the quantitative study and we applied a method of calibration on margins. However, for the qualitative study, what is sought is diversity of opinion, not representativeness. In grounded theory, it is recommended to vary the sample according to the hypotheses that emerge from the analysis of the interviews. This is why our sample was varied regarding the gender of the participants, their study fields and their smoking status. We thus followed the usual practice of “purposive sampling”. It did not emerge during our analysis that the age of the participants was a major factor explaining their vaping experience. We did not take this factor into account when diversifying our sample.

The quantitative study was led among students regardless of their smoking status and vaping status. The aim of this study was to estimate the prevalence of e-cigarette use and it would not have been relevant to exclude non-vapers. The qualitative study was led among regular e-cigarette users only (using at least 2 consecutive months). This choice can be explained by the desire to go deeper in the understanding of behaviors by describing the students' experience of vaping and the relationship they make between this use and their current or past use of tobacco. For this reason, we interviewed 7 students who had participated in the quantitative study, but we allowed ourselves to go beyond the framework of this participation. We increased our recruitment capacity by snowball strategy and by inviting students to participate also through social networks.

Component of thematic/ content analysis should have been described in the results to display it in the form of themes rather than giving individual reason/s.

Materials and methods:

We thank the reviewer for this comment. We modified the first part of the results to better highlight the main themes related to reasons for using e-cigarettes. We also added 2 figures to the manuscript to illustrate the organization into main themes on reasons for experimenting with e-cigarettes (figure 2) and reasons for current use (figure 3).

Duration of data collection appear too long for 20 interviews.

The interviews were rich: we wanted the students to discuss the reasons for using e-cigarettes but also their experience of vaping, from the first attempt to current use. The interviews lasted on average 55.25 minutes (minimum: 15 minutes; maximum: 120 minutes). We have added the average length of the interviews to the revised manuscript and the final guide in Supporting information.

Strengths and limitations have to be described.

We thank the reviewer for this comment. We had already described the limitations of our work; we added a few sentences on the strengths.

Conclusion is to be re- stated by emphasizing triangulation of findings of both quantitative and qualitative study and also separately.

Conclusion:

We first summarized the results of the quantitative and qualitative studies separately, and we added sentences to explain the importance or the mixed approach to understand why, among so many experimenters, few students remain in regular use.

References are validated and cited correctly in the text.

Overall quality of work is acceptable with major revision in analysis and presentation of results.

We thank the reviewers and the editorial board for their comments and the opportunity to resubmit this manuscript after revision.
