# Supplementary material for: Prevalence, lived experiences and user profiles in e-cigarette use: A mixed methods study among French college students
Source: PLoS One. 2024 Feb 9;19(2):e0297156. doi: 10.1371/journal.pone.0297156 (PMC10857705; doi:10.1371/journal.pone.0297156)
Supplement: Attachment — Submitted filename: Response to Reviewers_PLOS ONE_V7.docx [file pone.0297156.s009.docx]

To:

Lambros Lazuras
Academic Editor
PLOS ONE

Bordeaux, October 26^th^, 2022

Object: Response to reviewers’

Dear Pr Lambros Lazuras,

We are very grateful that you considered our article “**Prevalence, lived experiences and user profiles in e-cigarette use: a mixed methods study among French college students**” for revision despite the overall negative comment of reviewer #3.

We would have loved to respond point by point to reviewer #3 but this is made difficult because of the very short commentary provided by this reviewer.

Reviewer #3 answered “no” to question 3 about the appropriateness of the statistical analysis but the only comment given by the reviewer was that “*the methodology and analysis for qualitative component of the study did not satisfy the scientific standards as such*.” We will therefore concentrate on that, and we provide below explanations on the **qualitative component** and **the final integrative phase**.

**1. We conducted a sequential explanatory mixed methods study (QUANT → qual).** This study design described by Creswell JW et al (1,2) consists of collecting and analyzing quantitative and qualitative data separately and then interpreting them together. During this integrative phase, the qualitative results deepen one or more results obtained in the quantitative phase. As written by Ivankova NV et al. "*the qualitative data are collected and analyzed second in the sequence and help explain, or elaborate on, the quantitative results obtained in the first phase*" (2).

This study design allows to deepen some results of the quantitative stage but its main weakness is the possible duration of the qualitative stage (1,2) as it is designed to answer questions emerging from the quantitative stage. This explains why the qualitative phase could take some time (which was noted by reviewer #3 during the initial evaluation).

At the end of our quantitative stage, one question remained: as many student smokers try e-cigarettes, why few of them engage in its regular use? The quantitative phase, although taking into account various factors (socio-demographic, academic, use of psychoactive products, etc.) could not allow us to understand in depth why and how some students engage in regular vaping. We therefore clearly prioritize this question in our qualitative phase. As noted by Ivankova NV et al. : "*depending on the study goals, the scope of quantitative and qualitative research questions, and the particular design of each phase, a researcher may give the priority to the qualitative data collection and analysis*” (2).

There are several interpretative approaches described in mixed-method studies for integrative phase (3). The connecting approach would impose a complete recruitment of the qualitative component participants among the participants of the quantitative component. The building approach that we chose requires that results from one data collection supports the next data collection. A partial connection of the two samples is possible if it makes it possible to deepen the results obtained during the first step.

**2. Regarding the qualitative phase itself**. We conducted two separate analyses of the twenty interviews. The first and main analysis was inspired by Grounded theory (4,5). Its objective was to answer to the question emerging at the end of the quantitative phase. We applied its main principles: openness, analyzing immediately, coding and the constant comparative method, theoretical sampling, theoretical saturation, and production of a substantive theory (5).

We also conducted in parallel an inductive thematic analysis of the same interviews from a constructionist perspective. This supplementary analysis made by a group of three medical students (cited in the acknowledgments) supervised by the first author, allowed us to explore a secondary objective: to describe the reasons for experimenting with or continuing to use e-cigarettes. This supplementary analysis was possible because “*thematic analysis is not wedded to any pre-existing theoretical framework, and therefore it can be used within different theoretical frameworks*” said Braun V and Clarke V (6). The constructionist perspective assumes that “*meaning and experience are socially produced and reproduced, rather than inhering within individuals*” (6). As it was a secondary analysis, we have not detailed the data analysis procedure, so as not to lengthen the article. It was nevertheless carried out according to the six steps described by Braun and Clarke and resulted in figures 2 and 3. This double analysis of the interviews also explains why it was sometimes necessary to be 5 coders, as well as the duration perceived as unusually long for the qualitative component.

**3. Participants selection.** Seven volunteer students from the quantitative phase agreed to participate in the qualitative phase. However, it appeared during the analysis of these interviews that they alone did not make it possible to develop a sufficiently stable formal theory on how and why certain students commit themselves to vaping in France. As Ivankova NV et al. said "*In the sequential explanatory design, a researcher typically connects the two phases while selecting the participants for the qualitative follow-up analysis based on the quantitative results from the first phase*" (2). The selection of participants for the qualitative component must therefore consider the results of the quantitative component but it is not required that all participants in the qualitative study have participated in the other phase. What matters is that they are recruited according to characteristics that allow to answer the questions posed by the first stage. The differences between our two samples surprised the third reviewer in her first critical reading who pointed “*qualitative study sample is said to be from quantitative one but differ a lot in its composition according to age and gender; that might affect interpretation of findings*”. Our qualitative sample varied regarding the gender of the participants, their study fields, and their smoking status, following the usual practice of theoretical sampling. It did not emerge during our analysis that the age of the participants was a major factor explaining their vaping experience. We did not take this factor into account when diversifying our sample. Theorical sampling made possible to embed the two phases of our mixed-methods study by selecting participants based on the question posed by the first part and the ideas emerging during the analysis of the qualitative data.

**4. Guidelines.** We followed the COREQ guidelines for describing the qualitative component in the manuscript (7).

**5. Previous experience with qualitative studies.** Our research team has already published few articles of qualitative research, in particular with mixed method research:

- Chamon Q Govindin Ramassamy K, Rahis AC, Guignot L, Tzourio C, Montagni I. Persistence of Vaccine Hesitancy and Acceptance of the EU Covid Certificate Among French Students. J Community Health 2022;47(4):666-73. doi: 10.1007/s10900-022-01092-6.
- Montagni I, Abraham M, Tzourio C, Luquiens A, Nguyen-Thanh V, Quatremere G. Mixed-methods evaluation of a prevention campaign on binge drinking and cannabis use addressed to young people. Journal of Substance Use 2022. doi: 10.1080/14659891.2021.2022223.
- Montagni I, Vialemaringe M, Tzourio C. Sport practice and perceptions in university students: a mixed-methods study. International Sport Science Student Studies 2020;2(1):1-15.
- Montagni I, Langlois E, Koman J, Petropoulos M, Tzourio C. Avoidance and Delay of Medical Care in the Young: An Interdisciplinary Mixed-methods Study. YOUNG 2017; 26(5):1-20. doi: 10.1177/1103308817734474.

One of our co-authors - Emmanuel Langlois - is a sociologist working on addictions and drug abuse in the young. He is a lecturer at the University of Bordeaux and his personal references can be found here: <https://orcid.org/0000-0001-9733-5808>

We hope that these explanations will assure you that we have conducted this study in the most scientifically rigorous manner possible. We also maintain that our conclusions are supported by the data. We believe that the misunderstanding of reviewer #3 on our qualitative analysis may be due to the lack of precision in the article about the two separate analyses led on the same qualitative data and we have added this precision in our revised version. The changes only affect the main text. Tables, figures, additional files, funding sources, author contributions and affiliations, acknowledgments, conflict of interest statement remain unaffected.

The work has not been published elsewhere, nor is it currently under consideration for publication elsewhere. All authors have sufficiently participated in the paper to fully support it and have approved this revised version. The authors declare that they have no conflicts of interest.

Thank you for considering our revised manuscript.

On behalf of the authors,

Yours sincerely,

Shérazade KINOUANI, Corresponding author

Université de Bordeaux, INSERM U1219, BPH, équipe HEALTHY

146, rue Léo Saignat - CS 61292 - 33076 Bordeaux Cedex France

sherazade.kinouani@u-bordeaux.fr

Phone: + (33)6.10.29.68.47

**References**

1.	Creswell JW, Clark VLP. Designing and conducting mixed methods research. Thousand Oaks, CA, US: Sage Publications, Inc; 2007.

2.	Ivankova NV, Creswell JW, Stick SL. Using Mixed-Methods Sequential Explanatory Design: From Theory to Practice. Field Methods. 2006;18(1):3–20.

3.	Fetters MD, Curry LA, Creswell JW. Achieving Integration in Mixed Methods Designs—Principles and Practices. Health Serv Res. 2013;48(6pt2):2134–56.

4.	Glaser BG, Strauss AL. The discovery of grounded theory: strategies for qualitative research. 5th edition. New Brunswick, New Jersey: Aldine Transaction; 2010.

5.	Sbaraini A, Carter SM, Evans RW, Blinkhorn A. How to do a grounded theory study: a worked example of a study of dental practices. BMC Med Res Methodol. 2011;11(1):128.

6.	Braun V, Clarke V. Using thematic analysis in psychology. Qual Res Psychol. 2006;3(2):77–101.

7.	Tong A, Sainsbury P, Craig J. Consolidated criteria for reporting qualitative research (COREQ): a 32-item checklist for interviews and focus groups. Int J Qual Health Care. 2007;19(6):349–57.
