# Supplementary material for: Prevalence, lived experiences and user profiles in e-cigarette use: A mixed methods study among French college students
Source: PLoS One. 2024 Feb 9;19(2):e0297156. doi: 10.1371/journal.pone.0297156 (PMC10857705; doi:10.1371/journal.pone.0297156)
Supplement: Attachment — Submitted filename: REVIEWERs comments 21-33747 R2.odt [file pone.0297156.s010.odt]

REVIEWER’S COMMENTS: 
Title: “Prevalence, lived experiences and user profiles in e-cigarette use: A mixed methods study among French college students.” [Revision 2]
I am thankful to the editor to keep me in loop as a reviewer on this manuscript. The authors have made conscious effort to improve the manuscript. I like to guide my dear authors that the comments are basically raised to improve the quality of scientific material and never meant to be negative or discouraging towards researchers.
But one thing is to be ensured that qualitative studies are carried out in order to bring truth and a reality check in your findings. So, selection of participants very crucial as all the more you adopted a grounded theory approach that is very sensitive to type and number of participants
The methodological clarity has been explained a bit in this version. 
Type of analysis mentioned by the authors is thematic analysis that is not adequately shown in results.
In fact, by reading the results, analysis fits more in narrative analysis rather content analysis or thematic analysis.
Results section still not incorporated the findings of qualitative component of study.
Conclusion also inappropriate as the objectives of qualitative component were not included in it.

DECSISION: MAJOR REVISION
Thanks,
